# Supplementary material for: Direct asymmetric synthesis of β-branched aromatic α-amino acids using engineered phenylalanine ammonia lyases
Source: Nat Commun. 2024 Sep 26;15:8264. doi: 10.1038/s41467-024-52613-x (PMC11427684; doi:10.1038/s41467-024-52613-x)
Supplement: Supplementary file 1 — Supplementary Information [file 41467_2024_52613_MOESM1_ESM.pdf]

Supporting Information

Direct Asymmetric Synthesis of β-Branched Aromatic α-Amino Acids using Engineered Phenylalanine Ammonia Lyases

Chenghai Sun,*[a] Gen Lu,[b] Baoming Chen,[b] Guangjun Li,[b] Ya Wu,[b] Yannik Brack,[a] Dong Yi,[c] Yufei Ao,[a] Shuke Wu,[d] Ren Wei,[a] Yuhui Sun,[b] Guifa Zhai,*[b] and Uwe T. Bornscheuer*[a]

[a] Department of Biotechnology and Enzyme Catalysis, Institute of Biochemistry, University of Greifswald, Greifswald, Germany.

[b] School of Pharmacy, Tongji Medical College of Huazhong University of Science and Technology, Hubei Key Laboratory of Natural Medicinal Chemistry and Resource Evaluation, Wuhan, China.

[c] Research Center for Systems Biosynthesis, China State Institute of Pharmaceutical Industry, National Key Laboratory of Lead Druggability Research, Shanghai, China.

[d] College of Life Science and Technology, Huazhong Agriculture University, Wuhan, China.

Corresponding authors:

Chenghai Sun (chenghai.sun23@outlook.com)

Guifa Zhai (gfzhai@hust.edu.cn)

Uwe T. Bornscheuer (uwe.bornscheuer@uni-greifswald.de).

Table of Contents

1. Experimental Procedures

1.1. General materials and methods

1.2. Synthesis of β-branched aromatic unsaturated ethylenic acid

1.3. Optimization of the *in vivo* amination reactions

1.4. Determination of optical purity of the amination products

1.5. Data used to characterize the β-branched aromatic α-amino acid produced

2. Supplementary Tables

Table S1. Strains, plasmids and primers used in this study

Table S2. X–ray crystallographic data of **4a**

Table S3. X–ray crystallographic data of **5a**

Table S4. X–ray crystallographic data of **8a**

Table S5. X–ray crystallographic data of **9a**

Table S6. Angle and distance statistics of key atoms after QM/MM optimized structures

3. Supplementary Figures

Figure S1. Proposed mechanism for the amination reaction mediated by PcPAL.

Figure S2. Docking results and the potentials of mean force results.

Figure S3. Computational results of cMD.

Figure S4. Statistical distances in the umbrella sampling (US) simulations.

Figure S5. Standard curve of (2*S*,3*R*)-2-amino-3-phenyl-butyric acid and *in vitro* amination assays of different variants against β-methylcinnamic acid.

Figure S6. kinetic contents result.

Figure S7. LC-HRMS data of Marfey's reagent derivatization products of 2-amino-3-phenyl-butyric acid isomers.

Figure S8. Optimization of the *in vivo* amination reaction conditions.

Figure S9. Overview of the chemically synthesized substrates for this study.

Figure S10. HPLC analysis of PcPAL mutants against different substrates.

Figure S11. 12% SDS-PAGE analysis of the purified PcPALs

Figure S12. NMR spectra of **2**

Figure S13. NMR spectra of **3**

Figure S14. NMR spectra of **4**

Figure S15. NMR spectra of **5**

Figure S16. NMR spectra of **6**

Figure S17. NMR spectra of **7**
Figure S18. NMR spectra of **8**
Figure S19. NMR spectra of **9**
Figure S20. NMR spectra of **10**
Figure S21. NMR spectra of **11**
Figure S22. NMR spectra of **12**
Figure S23. NMR spectra of **13**
Figure S24. NMR spectra of **14**
Figure S25. NMR spectra of **15**
Figure S26. NMR spectra of **16**
Figure S27. HRMS and NMR spectra of **1a**
Figure S28. HRMS and NMR spectra of **2a**
Figure S29. HRMS and NMR spectra of **3a**
Figure S30. HRMS and NMR spectra of **4a**
Figure S31. HRMS and NMR spectra of **5a**
Figure S32. HRMS and NMR spectra of **6a**
Figure S33. HRMS and NMR spectra of **7a**
Figure S34. HRMS and NMR spectra of **8a**
Figure S35. HRMS and NMR spectra of **9a**
Figure S36. HRMS and NMR spectra of **10a**
Figure S37. Statistical distances in the umbrella sampling (US) simulations
Figure S38. Overlap check of specific windows with histograms
Figure S39. Molecular dynamics analysis of hydrogen bonds in protein-substrate complexes

4. Supplementary References

1. Experimental Procedures

1.1. General materials and methods.

Escherichia coli strains were cultivated and manipulated according to standard methods[1]. Primer synthesis and DNA sequencing were performed at Eurofins Genomics (Germany). All the strains, plasmids and primers used in this study are listed in **Table S1**. All chemicals and reagents were purchased from Sigma (Germany). HPLC analysis was carried out on a SHIMADZU LC-30A UPLC system. NMR data were collected on Bruker DRX-400 or DRX-600 NMR spectrometers (Bruker Co. Ltd., Germany). PyMOL (version 1.8.0.2) was used to create mutants, visualize 3D structure analysis and comparison. Docking experiments were carried out by using AutoDock Tools (version 1.5.6). Conventional molecular dynamics (cMD) and quantum mechanics/molecular mechanics (QM/MM) simulations were performed using the software AMBER 22 and ORCA 5.04, respectively. Umbrella sampling simulations were performed via XTB 6.6.1 software.

1.2. Synthesis of β-branched aromatic unsaturated ethylenic acid.

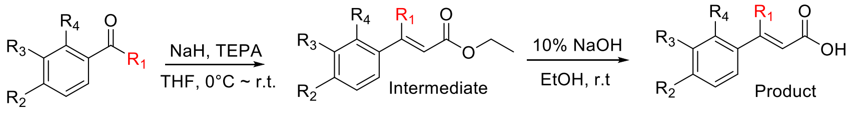

R1: Me, CF_3, CH_2CH_3, $CH_2CH_2CH_3$, $CH(CH_3)_2$; **R2, R3, R4**: Br, F, I, Me, OMe, NO_2, Naphthalene

The target products were synthesized following established literature procedures[2] with minor modifications as described below. Sodium hydride (NaH) (1.5 equiv) was added dropwise to a solution of triethylphosphonoacetate (TEPA) (1.5 equiv) in dry tetrahydrofuran (THF) (20 mL) at 0 °C under a nitrogen atmosphere. The mixture was stirred at room temperature for 1 h. Subsequently, the corresponding ketone (100 mg, 1.0 equiv) was added and the reaction was stirred for an additional 10 h. At this point, the reaction was quenched by the addition of 1N hydrochloric acid (HCl), and the aqueous layer was extracted with ethyl acetate (EA) (3 x). The organic layers were combined, dried over anhydrous sodium sulfate (Na_2SO_4), filtered, and concentrated under vacuum. The resulting crude product was used for column chromatography to separate and purify the desired E-type unsaturated ethylenic acid ester (intermediate). The purified intermediate was then dissolved in 10 mL ethanol (EtOH). Subsequently, 5 mL 10% sodium hydroxide (NaOH) was added dropwise, and the solution was left at room temperature for overnight. The pH of the mixture was then adjusted to 3 using 1 M HCl, and the resulting mixture was extracted with ethyl acetate (EA) (3 x). The organic phase was dried over anhydrous sodium sulfate

(Na_2SO_4), filtered, and concentrated under vacuum. Finally, the β-branched aromatic unsaturated ethylenic acid was recrystallized from EA using petroleum ether (PE) as a solvent.

Synthesis of **2**:

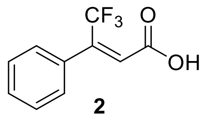

2

2: white solid (110 mg, 89% yield). ^{1}H-NMR (300 MHz, MeOD-d4): δ 7.33 (dd, J = 6.6, 2.9 Hz, 2H), 7.24 (dd, J = 6.2, 2.7 Hz, 3H), 6.68 (q, J = 1.5 Hz, 1H); ^{13}C-NMR (75 MHz, MeOD-d4): δ 171.6, 132.62, 132.57, 132.4, 130.1, 129.7, 128.9, 128.2, 127.7, 125.8, 122.2.

Synthesis of **3**:

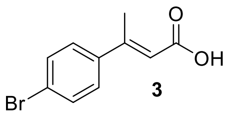

3

3: white solid (101 mg, 82% yield). ^{1}H-NMR (300 MHz, $CDCl_3$): 7.63 (t, J = 1.8 Hz, 1H), 7.51 (ddd, J = 7.9, 1.9, 1.0 Hz, 1H), 7.42 (ddd, J = 7.8, 1.7, 1.1 Hz, 1H), 7.30 – 7.26 (m, 1H), 6.15 (q, J = 1.2 Hz, 1H), 2.57 (d, J = 1.3 Hz, 3H); ^{13}C-NMR (75 MHz, $CDCl_3$): 171.18, 156.83, 144.08, 132.24, 130.13, 129.53, 125.04, 122.77, 117.27, 18.28.

Synthesis of **4**:

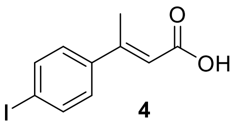

4

4: white solid (105.3 mg, 90% yield). ^{1}H-NMR (400 MHz, MeOH-d4): 7.65 (d, J = 8.6 Hz, 2H), 7.20 (d, J = 8.6 Hz, 2H), 6.04 (q, J = 1.3 Hz, 1H), 2.41 (d, J = 1.3 Hz, 3H); ^{13}C-NMR (101 MHz, MeOH-d4): 170.03, 155.66, 143.23, 139.06, 129.38, 118.91, 95.73, 17.95.

Synthesis of **5**:

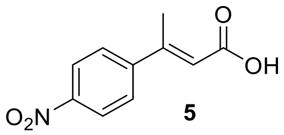

5

5: white solid (107.5 mg, 86% yield). ^{1}H-NMR (400 MHz, MeOH-d4): 8.27 – 8.25 (m, 2H), 7.74 (d, 2H), 6.24 (s, 1H), 2.55 (d, J = 1.4 Hz, 3H); ^{13}C-NMR (101 MHz, MeOH-d4): 169.8, 153.3, 150.0, 128.6, 124.7, 121.8, 17.9.

Synthesis of **6**:

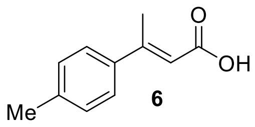

6

6: white solid (113.9 mg, 87% yield). ^{1}H-NMR (400 MHz, MeOH-d4): 7.30 (d, J = 8.2 Hz, 2H), 7.09 (d, J = 8.0 Hz, 2H), 6.01 (d, J = 1.2 Hz, 1H), 2.42 (d, J = 1.2 Hz, 3H), 2.24 (s, 3H); ^{13}C-NMR (101 MHz, MeOH-d4): 170.43, 157.10, 140.68, 140.57, 130.39, 127.39, 117.41, 21.33, 18.12.

Synthesis of **7**:

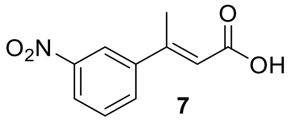

7

7: white solid (110.5 mg, 85% yield). ^{1}H-NMR (600 MHz, MeOH-d4): 8.29 (s, 1H), 8.23 (d, J = 8.1 Hz, 1H), 8.02 (d, J = 7.7 Hz, 1H), 7.70 (t, J = 8.0 Hz, 1H), 6.24 (s, 1H), 2.52 (s, 3H); ^{13}C-NMR (151 MHz, MeOH-d4): 167.33, 151.07, 148.12, 143.12, 132.85, 130.31, 123.67, 120.93, 119.93, 40.05, 39.93, 39.79, 39.65, 39.51, 39.37, 39.23, 39.09, 17.24.

Synthesis of **8**:

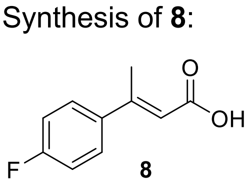

8

8: white solid (106.6 mg, 82% yield). ^{1}H-NMR (400 MHz, MeOH-d4): 7.44 (dd, J = 8.0, 1.1 Hz, 2H), 7.01 (t, J = 7.5, 1.2 Hz, 2H), 6.00 (d, J = 1.4 Hz, 1H), 2.42 (d, J = 2.5 Hz, 3H); ^{13}C-NMR (101 MHz, MeOH-d4): 170.12, 166.04, 163.58, 155.80, 139.88, 139.85, 129.62, 129.53, 118.39, 116.60, 116.39, 18.24.

Synthesis of **9**:

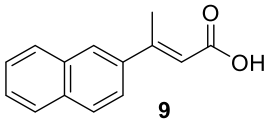

9

9: white solid (110.4 mg, 89% yield). ^{1}H-NMR (400 MHz, MeOH-d4): 7.92 (s, 1H), 7.83 (m, 1H), 7.78– 7.76 (m, 2H), 7.55 (dd, J = 8.7, 1.9 Hz, 1H), 7.41 (dd, J = 6.3, 3.2 Hz, 2H), 6.20 (s, 1H), 2.56 (s, 3H); ^{13}C-NMR (101 MHz, MeOH-d4): 170.30, 156.79, 140.79, 135.19, 134.85, 129.75, 129.41, 128.72, 127.94, 127.70, 127.11, 125.05, 118.78, 18.21.

Synthesis of **10**:

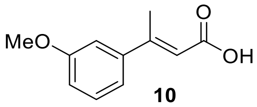

10

10: white solid (110.1 mg, 86% yield). ^{1}H-NMR (400 MHz, MeOH-d4): 7.19 (t, J = 8.0 Hz, 1H), 6.97 (ddd, J = 7.8, 1.7, 0.9 Hz, 1H), 6.92 – 6.90 (m, 1H), 6.83 (ddd, J = 8.3, 2.5, 0.8 Hz, 1H), 6.01 (s, 1H), 3.71 (s, 3H), 2.42 (d, J = 1.3 Hz, 3H); ^{13}C-NMR (101 MHz, MeOH-d4): 170.23, 161.42, 157.05, 145.24, 130.80, 119.85, 118.50, 115.60, 113.20, 55.91, 18.38.

Synthesis of **11**:

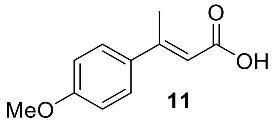

11

11: white solid (112.6 mg, 88% yield). ^{1}H-NMR (600 MHz, MeOH-d4): 7.52 – 7.47 (m, 1H), 6.97 – 6.90 (m, 1H), 6.10 (q, J = 1.1 Hz, 1H), 3.82 (s, 1H), 2.52 (d, J = 1.2 Hz, 1H); ^{13}C-NMR (151 MHz, MeOH-d4): 170.55, 162.28, 156.69, 135.61, 128.88, 116.33, 115.08, 55.93, 17.95.

Synthesis of **12**:

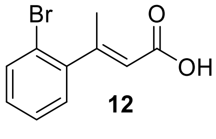

12

12: white solid (106.5 mg, 88% yield). ^{1}H-NMR (400 MHz, MeOH-d4): 7.61 (d, J = 8.4 Hz, 1H), 7.37 (t, J = 7.5 Hz, 1H), 7.22 (t, J = 3.7 Hz, 2H), 5.76 (s, 1H), 2.44 (d, J = 1.4 Hz, 3H); ^{13}C-NMR (101 MHz, MeOH-d4): 169.58, 158.39, 146.19, 134.22, 130.60, 130.13, 128.90, 121.97, 121.54, 20.81.

Synthesis of **13**:

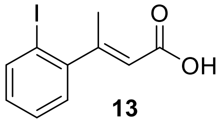

13

13: white solid (101.8 mg, 87% yield). ^{1}H-NMR (400 MHz, MeOH-d4): 7.88 (dd, J = 8.0, 1.1 Hz, 1H), 7.40 (td, J = 7.5, 1.2 Hz, 1H), 7.19 (dd, J = 7.6, 1.7 Hz, 1H), 7.07 – 7.00 (m, 1H), 5.70 (q, J = 1.4 Hz, 1H), 2.41 (t, J = 2.5 Hz, 3H); ^{13}C-NMR (101 MHz, MeOH-d4): 169.58, 160.91, 150.17, 140.75, 130.41, 129.58, 128.96, 122.04, 95.88, 21.16.

Synthesis of **14**:

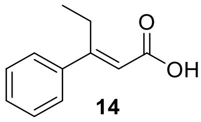

14

14: white solid (104 mg, 79.4% yield). ^{1}H-NMR (300 MHz, MeOD-d4): 7.40 (d, J = 7.5 Hz, 2H), 7.35 – 7.23 (m, 2H), 6.04 (s, 1H), 2.98 (q, J = 7.5 Hz, 2H), 0.96 (t, J = 7.5 Hz, 3H); ^{13}C-NMR (75 MHz, MeOD-d4): δ 174.93, 150.17, 142.01, 127.92, 127.16, 126.28, 124.24, 23.23, 12.50.

Synthesis of **15**:

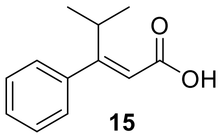

15

15: white solid (110.9mg, 87% yield). 1H-NMR (300 MHz, MeOD-d4): δ 7.19 – 7.08 (m, 5H), 5.59 (s, 1H), 3.75 (dt, J = 14.0, 7.0 Hz, 1H), 0.95 (s, 3H), 0.93 (s, 3H); 13C-NMR (75 MHz, MeOD-d4): δ 174.72, 153.77, 141.76, 128.04, 127.17, 126.32, 125.73, 29.54, 20.72.

Synthesis of **16**:

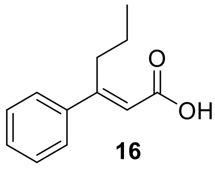

16

16: white solid (114.8 mg, 90% yield). ^{1}H-NMR (300 MHz, $CDCl_3$): δ 7.44 (dd, J = 4.2, 1.8 Hz, 2H), 7.37 (dd, J = 5.0, 1.8 Hz, 3H), 6.07 (s, 1H), 3.18 – 3.02 (m, 2H),

1.46 (dd, J = 15.2, 7.5 Hz, 2H), 0.93 (t, J = 7.4 Hz, 3H); ^{13}C-NMR (75 MHz, $CDCl_3$): δ 163.02, 141.33, 129.06, 128.55, 126.77, 117.03, 32.92, 22.30, 14.03.

1.3. Optimization of the *in vivo* amination reactions

Initially, we conducted screenings involving different ammonia sources and diverse reaction formats. Following the expression of PcPAL variants in the *E. coli* BL21 (DE3)_ΔtyrB strain[3], whole cells were collected and reconstituted in either a solution of NH_4OH (5.0 M, pH 10.0) or $NH_2CO_3NH_4$ (4 M, pH 10), achieving an OD_{600} = 30. From these suspensions, 10 mL of each was reserved for subsequent reactions, while the remaining portions were subjected to sonication to facilitate lysis, followed by centrifugation (4 °C, 15 min, 8,000× *g*) to pellet cellular debris. The resulting supernatant was directly employed in subsequent reactions. To either the whole cell suspension or the clarified lysate (5 mL), β-methyl cinnamic acid (1 mM) was added. These reactions were placed at 250 rpm and 30 °C for 24 h. Then 100 μL samples were extracted and supplemented with 100 μL aq. H_2SO_4 (10% v/v) to stop the reaction. Subsequent centrifugation (12,000 × *g*, 4 °C, 2 min) separated the supernatant, which was then subjected to filtration and directly injected into an HPLC apparatus for analysis.

Optimization continued with the selection of the superior ammonia source (NH_4OH solution) and the utilization of the whole-cell reaction biotransformation. Substrate concentrations and reaction duration were further optimized. Four different substrate concentrations (0.5 g/L, 1.0 g/L, 1.5 g/L, and 2.0 g/L) were studied, and the whole-cell biotransformation reactions were conducted at 30 °C, with samples retrieved at various time intervals. By analyzing the time curves, it was determined that the optimal substrate concentration was 1.0 g/L, and the highest yield was obtained after 18 h.

1.4. Determination of optical purity of the amination products

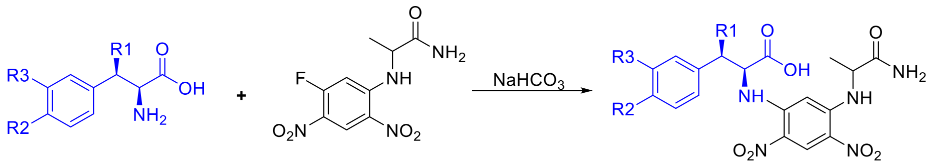

β-substituted amino acids FDNP-D/L-alaninamide (Marfey reagant) Derivatization product

According to a previous report[4], a solution of FDNP-alaninamide (33 mM) in acetone was first prepared. In a 2 mL vial, the corresponding amino acid (0.50 μmol) was dissolved in a 1 M aqueous solution of $NaHCO_3$ (100 μL). Subsequently, FDNP-alaninamide solution (10 μL, 0.33 μmol) was introduced to the vial. The vial was

incubated at 37°C and 500 rpm. After 5 h, the reaction mixture was allowed to cool down to room temperature and the reaction was stopped by adding 200 μL MeOH. The resulting solution underwent direct analysis by LC-HRMS (**Fig. S3**). Both racemic and enantiopure FDNP-alaninamide were utilized for the derivatization of each amino acid[5]. The derivatization reactions were analyzed using a Welch Ultimate XB-C18 column (5 μm, 250 × 4.6 mm) at a flow rate of 1 mL min-1 and detected at 340 nm over a 27 min gradient program with water containing 0.1% formic acid (eluent A) and MeOH (eluent B): T = 0 min, 30% B; T = 0 – 20 min, 95% B; T = 22 min, 95% B; T = 23 - 27 min, 30% B.

1.5. Data used for the characterization of the produced β-branched aromatic α-amino acid

Synthesis of **1a**:

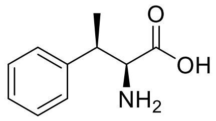

1a

Compound **1a**: white powder (23.4 mg, 71.4% yield, dr > 20:1, >99.5% ee). The absolute configuration of **1a** was determined by comparison with previously reported NMR and rotation data[4, 6]. The absolute configurations of the other amino acid products were either assigned by analogy to **1a** or by determining their X-ray crystallographic structures.

^{1}H NMR (400 MHz, D_2O): δ 7.48 – 7.45 (m, 2H), 7.42 – 7.38 (m, 3H), 4.25 (d, J = 5.7 Hz, 1H), 3.64 – 3.50 (m, 1H), 1.47 (d, J = 7.3 Hz, 3H).

^{13}C NMR (101 MHz, D_2O): δ 171.3, 139.2, 129.2, 128.1, 127.8, 58.8, 39.7, 14.7.

HRMS (ESI): calcd $C_{10}H_{14}NO_2^+$ ([M +H$^+$]) 180.1019, found 180.1018.

$[\alpha]_{25}$ = +16.9 (c = 1.0, MeOH).

HPLC traces of **1a** derivatized with FDNP-L-alaninamide and FDNP-D,L-alaninamide:

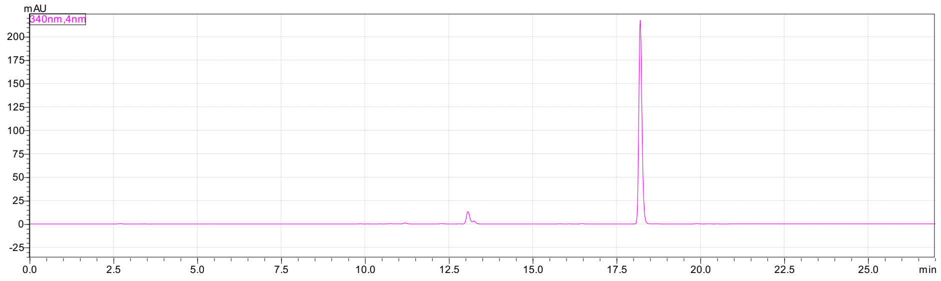

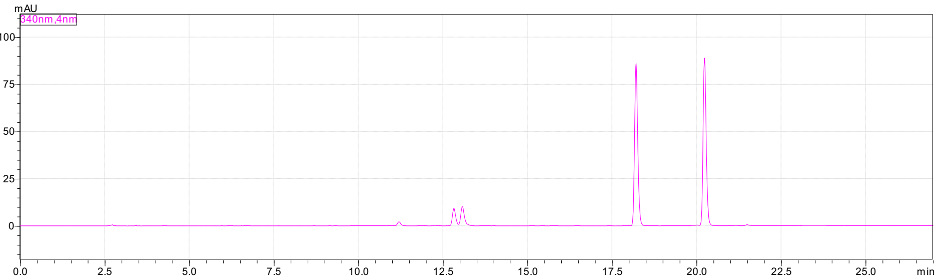

Synthesis of **2a**:

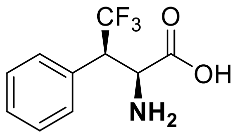

2a

Compound **2a**: white powder (13.2 mg, 41% yield, dr > 20:1, >99.5% ee).

^{1}H NMR (600 MHz, D_2O): δ 7.43 (m, 5H), 4.42 (d, J = 8.2 Hz, 1H), 4.20 (s, 1H).

^{13}C NMR (151 MHz, D_2O): δ 170.54, 131.11, 130.86, 130.62, 130.60, 130.43, 129.94, 128.60, 126.73, 55.28, 51.88, 51.69, 51.51, 51.33.

HRMS (ESI): calcd $C_{10}H_{11}F_3NO_2^+$ ([M +H$^+$]) 234.0736, found 234.0734.

[α]$_{25}$ = -21.3 (c = 1.0, MeOH).

HPLC traces of **2a** derivatized with FDNP-L-alaninamide and FDNP-D,L-alaninamide:

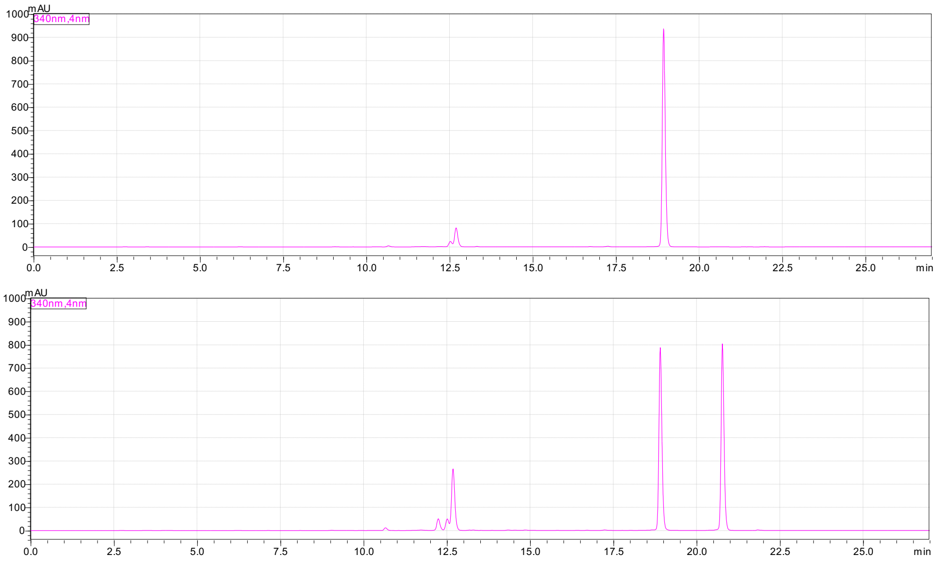

Synthesis of **3a**:

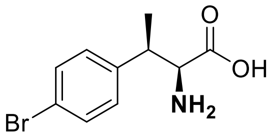

3a

Compound **3a**: white powder (23.4 mg, 70.3% yield, dr > 20:1, >99.5% ee).

^{1}H NMR (600 MHz, D$_2$O): δ 7.54 (s, 1H), 7.43 (d, J = 7.4 Hz, 1H), 7.33 (s, 1H), 7.29 (d, J = 6.9 Hz, 1H), 3.77 (s, 1H), 3.54 (s, 1H), 1.39 (s, 3H).

^{13}C NMR (151 MHz, D$_2$O): δ 173.2, 145.21, 132.15, 131.80, 131.64, 127.97, 123.99, 61.89, 40.97, 14.81.

HRMS (ESI): calcd $C_{10}H_{13}BrNO_2^+$ ([M +H$^+$]) 258.0124, found 258.0123 and 260.0101.

[α]$_{25}$ = -1.5 (c = 1.0, MeOH).

HPLC traces of **3a** derivatized with FDNP-L-alaninamide and FDNP-D,L-alaninamide:

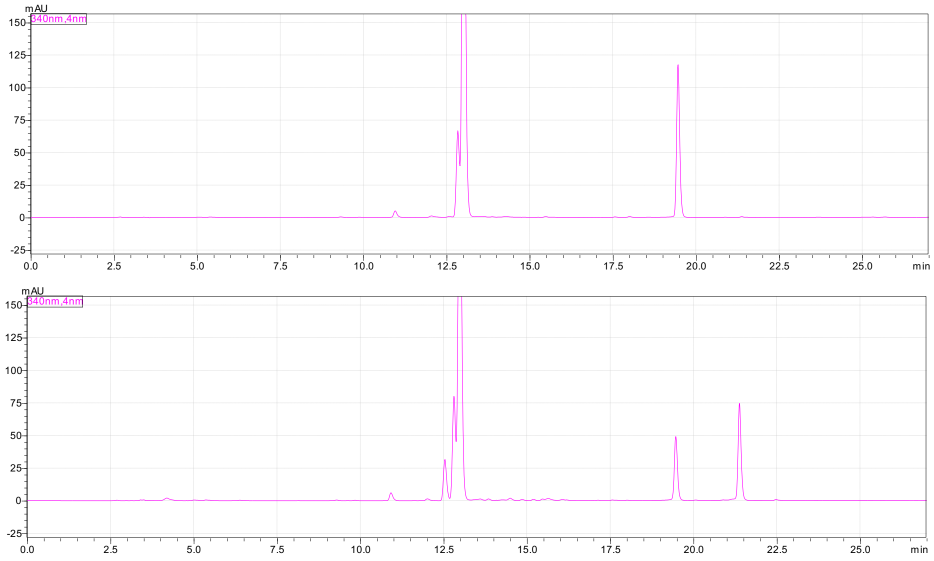

Synthesis of **4a**:

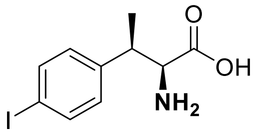

4a

Compound **4a**: white powder (20.8 mg, 65.4% yield, dr > 20:1, >99.5% ee).

^{1}H NMR (600 MHz, D$_2$O): δ 7.67 (d, J = 8.2 Hz, 2H), 7.10 (d, J = 8.3 Hz, 2H), 3.73 (dd, J = 42.5, 13.9 Hz, 1H), 3.54 – 3.43 (m, 1H), 1.34 (d, J = 7.3 Hz, 3H).

^{13}C NMR (151 MHz, D$_2$O): δ 173.03, 142.30, 139.20, 131.19, 93.51, 61.87, 40.83, 14.63.

HRMS (ESI): calcd $C_{10}H_{13}INO_2^+$ ([M +H$^+$]) 305.9985, found 305.9980.

[α]25 = -1.2 (c = 1.0, MeOH).

HPLC traces of **4a** derivatized with FDNP-L-alaninamide and FDNP-D,L-alaninamide:

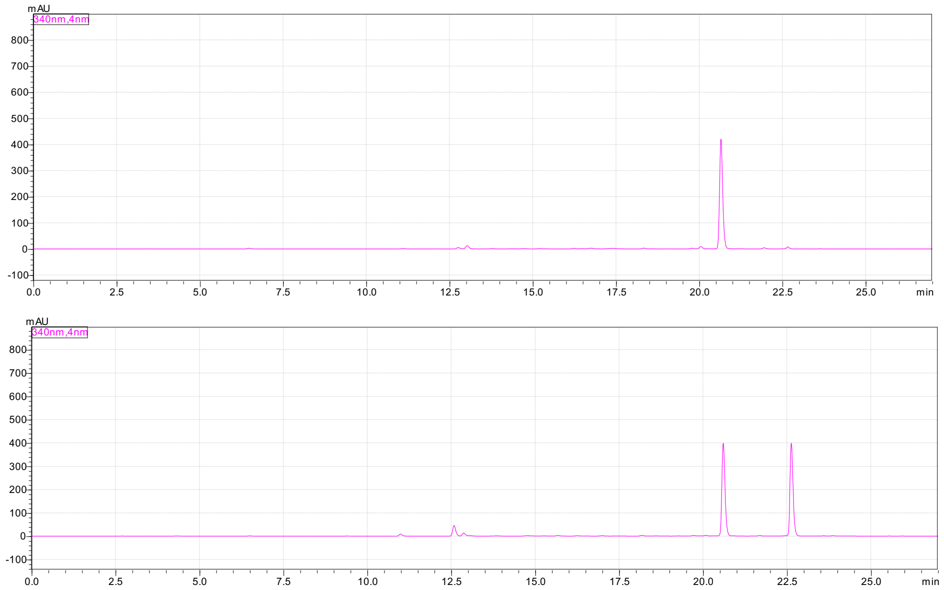

Synthesis of **5a**:

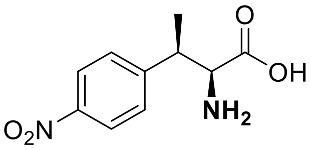

5a

Compound **5a**: white powder (21.0 mg, 64.7% yield, dr > 20:1, >99.5% ee).

^{1}H NMR (600 MHz, D_2O): δ 8.28 (d, J = 8.8 Hz, 2H), 7.60 (d, J = 8.7 Hz, 2H), 3.99 (d, J = 5.7 Hz, 1H), 3.70 – 3.62 (m, 1H), 1.47 (d, J = 7.2 Hz, 3H).

^{13}C NMR (151 MHz, D_2O): δ 172.71, 148.37, 146.96, 128.84, 124.00, 60.23, 39.73, 14.16.

HRMS (ESI): calcd $C_{10}H_{13}N_2O_4^+$ ([M +H$^+$]) 225.0869, found 225.0868.

[α]25 = +9.7 (c = 1.0, MeOH).

HPLC traces of **5a** derivatized with FDNP-L-alaninamide and FDNP-D,L-alaninamide:

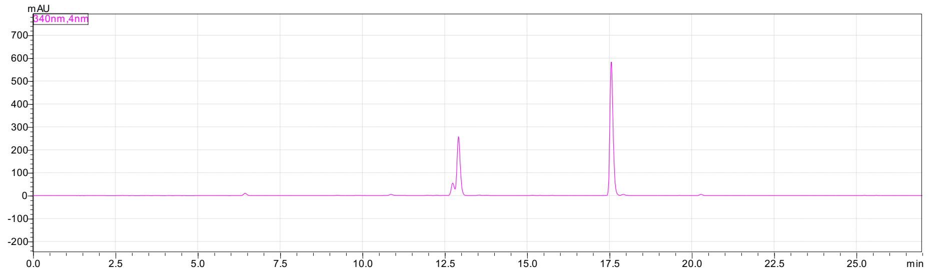

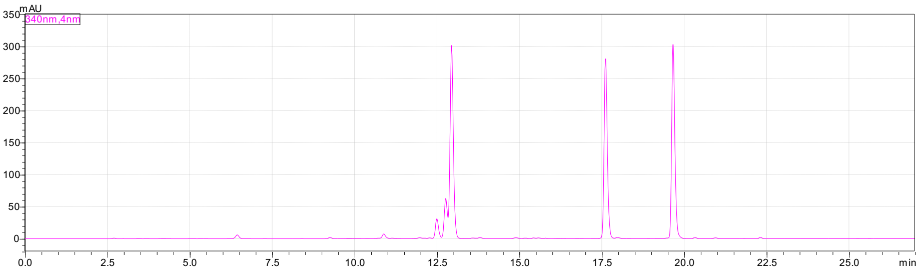

Synthesis of **6a**:

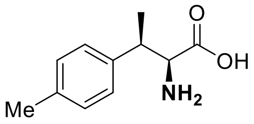

6a

Compound **6a**: white powder (16.9 mg, 51.4% yield, dr > 20:1, >99.5% ee).

^{1}H NMR (600 MHz, D_2O): δ7.23 (d, J = 7.7 Hz, 1H), 7.18 (d, J = 7.6 Hz, 1H), 3.75 (s, 1H), 3.54 (s, 1H), 2.32 (s, 1H), 1.37 (d, J = 7.1 Hz, 1H).

^{13}C NMR (151 MHz, D_2O): δ 139.27, 138.26, 130.70, 128.82, 62.19, 40.58, 21.20, 14.38.

HRMS (ESI): calcd $C_{11}H_{15}NO_2^+$ ([M +H$^+$]) 194.1175, found 194.1174.

$[\alpha]_{25}$ = -6.9 (c = 1.0, MeOH).

HPLC traces of **6a** derivatized with FDNP-L-alaninamide and FDNP-D,L-alaninamide:

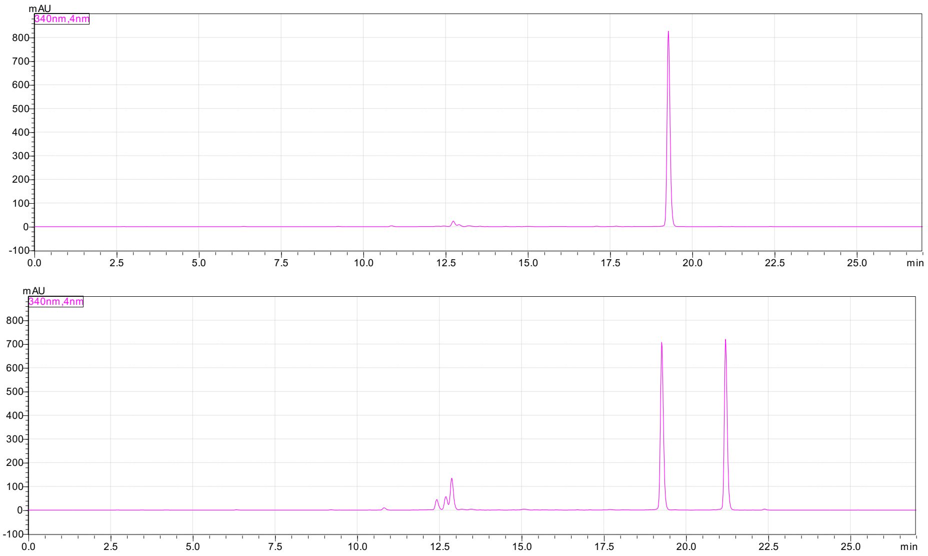

Synthesis of **7a**:

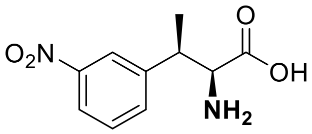

7a

Compound **7a**: white powder (17.6 mg, 54.1% yield, dr > 20:1, >99.5% ee).

^{1}H NMR (600 MHz, D_2O): δ 7.83 – 7.77 (m, 2H), 7.36 (d, J = 7.6 Hz, 1H), 7.23 (t, J = 7.9 Hz, 1H), 3.94 (d, J = 6.2 Hz, 1H), 3.29 – 3.23 (m, 1H), 1.11 (d, J = 7.2 Hz, 3H).

^{13}C NMR (151 MHz, D_2O): δ 170.23, 147.94, 140.84, 134.53, 130.03, 122.92, 122.52, 57.88, 39.34, 14.96.

HRMS (ESI): calcd $C_{10}H_{13}N_2O_4^+$ ([M +H$^+$]) 225.0869, found 225.0868.

[α]$_{25}$ = -10.3 (c = 1.0, MeOH).

HPLC traces of **7a** derivatized with FDNP-L-alaninamide and FDNP-D,L-alaninamide:

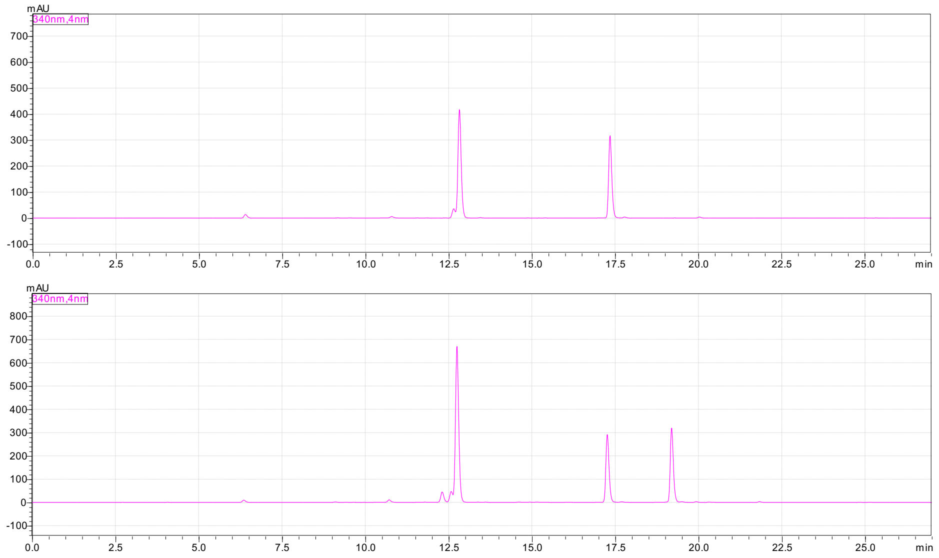

Synthesis of **8a**:

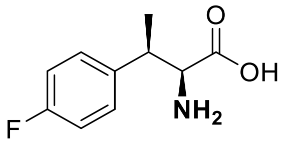

8a

Compound **8a**: white powder (21.8 mg, 66.7% yield, dr > 20:1, >99.5% ee).

^{1}H NMR (600 MHz, D_2O): δ 7.36 (dd, J = 8.5, 5.4 Hz, 2H), 7.08 (t, J = 8.7 Hz, 2H), 3.76 (d, J = 4.3 Hz, 1H), 3.54 (dd, J = 7.2, 4.4 Hz, 1H), 1.39 (d, J = 7.3 Hz, 3H).

^{13}C NMR (151 MHz, D_2O): δ 172.98, 164.45, 162.83, 138.31, 138.29, 130.86, 130.81, 116.70, 116.56, 61.97, 40.55, 15.08.

HRMS (ESI): calcd $C_{10}H_{13}FNO_2^+$ ([M +H$^+$]) 198.0924, found 198.0923.

[α]$_{25}$ = -1.2 (c = 1.0, MeOH).

HPLC traces of **8a** derivatized with FDNP-L-alaninamide and FDNP-D,L-alaninamide:

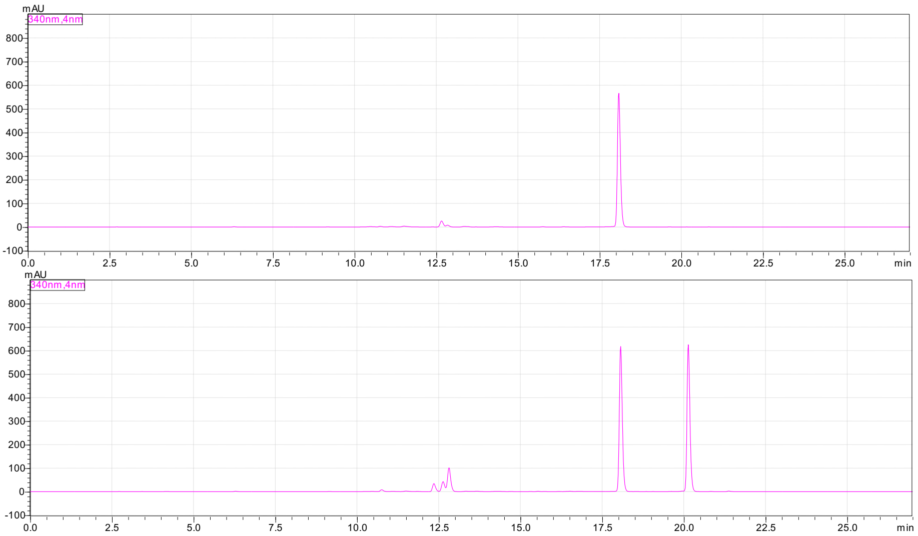

Synthesis of **9a**:

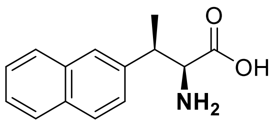

9a

Compound **9a**: white powder (20.7 mg, 64% yield, dr > 20:1, >99.5% ee).

^{1}H NMR (600 MHz, D_2O): δ 7.90 – 7.83 (m, 2H), 7.79 (s, 1H), 7.51 (dd, J = 8.5, 1.3 Hz, 1H), 7.46 (dd, J = 7.1, 1.3 Hz, 1H), 3.87 (d, J = 3.9 Hz, 1H), 3.75 (dd, J = 6.8, 4.1 Hz, 1H), 1.50 (d, J = 7.3 Hz, 3H).

^{13}C NMR (151 MHz, D_2O): δ 171.67, 138.29, 133.61, 132.81, 128.24, 127.49, 127.17, 125.83, 125.76, 125.54, 60.34, 39.53, 12.65.

HRMS (ESI): calcd $C_{14}H_{16}NO_2^+$ ([M +H$^+$]) 230.1175, found 230.1174.

$[\alpha]_{25}$ = -17.8 (c = 1.0, MeOH).

HPLC traces of **9a** derivatized with FDNP-L-alaninamide and FDNP-D,L-alaninamide:

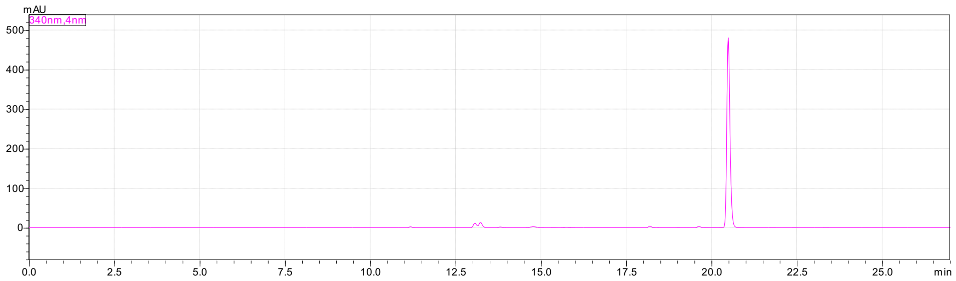

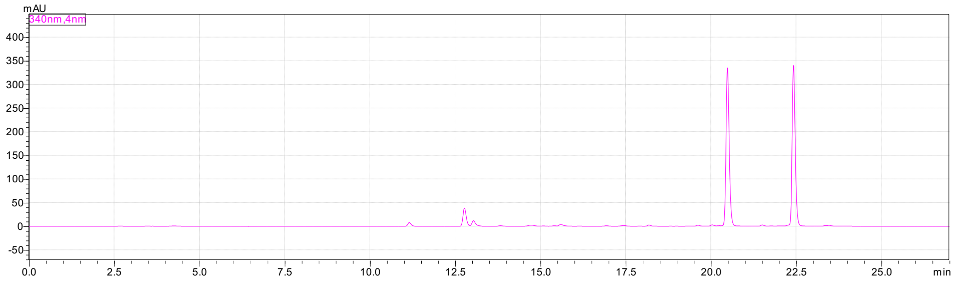

Synthesis of **10a**:

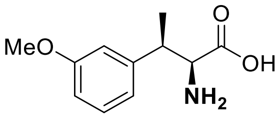

10a

Compound **10a:** white powder (14.1 mg, 43.3% yield, dr > 20:1, >99.5% ee).

^{1}H NMR (600 MHz, D_2O): δ 7.27 (t, J = 8.1 Hz, 1H), 6.96 – 6.90 (m, 2H), 6.84 (dd, J = 8.2, 2.0 Hz, 1H), 3.76 (d, J = 4.0 Hz, 1H), 3.60 – 3.53 (m, 1H), 1.37 (d, J = 7.3 Hz, 3H).

^{13}C NMR (151 MHz, D_2O): δ 173.14, 161.75, 144.02, 131.11, 121.06, 114.72, 113.96, 62.09, 55.78, 40.98, 14.19.

HRMS (ESI): calcd $C_{11}H_{16}NO_3^+$ ([M +H$^+$]) 210.1124, found 210.1123.

$[\alpha]_{25}$ = -11.6 (c = 1.0, MeOH).

HPLC traces of **10a** derivatized with FDNP-L-alaninamide and FDNP-D,L-alaninamide:

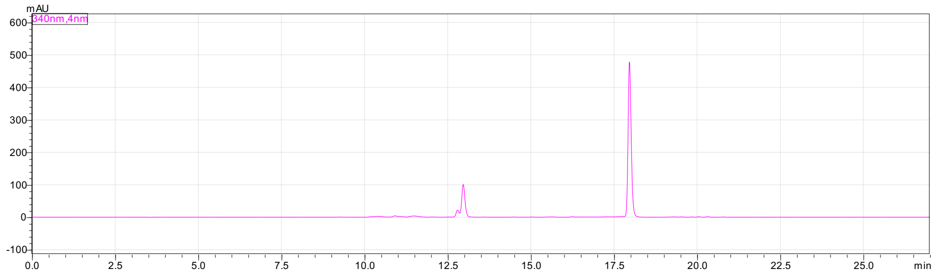

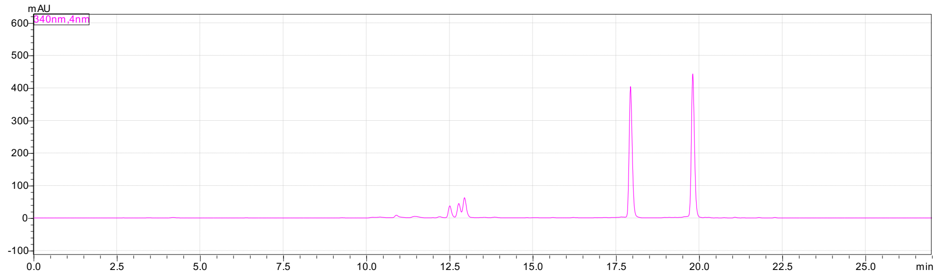

2. Supplementary Tables

Table S1. Strains, plasmids and primers used in this study

Strains	Description	Source
E. coli		
Top10	Host for general cloning	Lab storage
BL21 (DE3)	Host for protein expression	Lab storage
BL21 (DE3) ΔtyrB	Host for whole-cell biotransformation	Lab storage
**Plasmids**		
PcPALs_pETs	pETs derived, containing PcPALs	Lab storage
**Primers**		
PcPAL-L134A-F	5'-GCAGAAAGAAgctATCCGCTTCCTGAACGCTGG-3'	
PcPAL-L134G-F	5'-GCAGAAAGAAgctATCCGCTTCCTGAACGCTGG-3'	
PcPAL-L134V-F	5'-GCAGAAAGAAgctATCCGCTTCCTGAACGCTGG-3'	
PcPAL-L134X-R	5'-AGTGCACCCCCCTGTTTG-3'	
PcPAL-F137A-F	5'-ACTGATCCGCgctCTGAACGCTGG-3'	
PcPAL-F137G-F	5'-ACTGATCCGCggtCTGAACGCTGG-3'	
PcPAL-F137V-F	5'-ACTGATCCGCgttCTGAACGCTGG-3'	
PcPAL-F137X-R	5'-TCTTTCTGCAGTGCACCC -3'	
PcPAL-L138A-F	5'-GATCCGCTTCgctAACGCTGGTATCTTC-3'	
PcPAL-L138G-F	5'-GATCCGCTTCggtAACGCTGGTATCTTC-3'	
PcPAL-L138V-F	5'-GATCCGCTTCgttAACGCTGGTATCTTC-3'	
PcPAL-L138X-R	5'-AGTTCTTTCTGCAGTGCAC-3'	
PcPAL-L206A-F	5'-TTCCGGCGACgctGTTCCACTGTCC-3'	
PcPAL-L206G-F	5'-TTCCGGCGACggtGTTCCACTGTCC-3'	
PcPAL-L206V-F	5'-TTCCGGCGACgttGTTCCACTGTC-3'	
PcPAL-L206X-R	5'-GCAGTGATGGTACCACGC-3'	
PcPAL-L256A-F	5'-GAAAGAGGGCgctGCACTGGTTAAC-3'	
PcPAL-L256G-F	5'-GAAAGAGGGCggtGCACTGGTTAAC-3'	
PcPAL-L256V-F	5'-GAAAGAGGGCgttGCACTGGTTAAC-3'	
PcPAL-L256X-R	5'-GGTTGCAGTTCAAAGAAAC-3'	
PcPAL-N260A-F	5'-GGCACTGGTTgctGGTACCGCTGTTG-3'	
PcPAL-N260G-F	5'-GGCACTGGTTggtGGTACCGCTGTTG-3'	
PcPAL-N260V-F	5'-GGCACTGGTTgttGGTACCGCTGTTG-3'	
PcPAL-N260X-R	5'-AGGCCCTCTTTCGGTTGC-3'	
PcPAL-I460A-F	5'-AGGTGCAGAAggtGCTATGGCTTC-3'	

PcPAL-I460G-F	5’-AGGTGCAGAAggtGCTATGGCTTC-3’
PcPAL-I460V-F	5’-AGGTGCAGAAggtGCTATGGCTTC-3’
PcPAL-I460X-R	5’-TTGAAACCATAGTCCAGG-3’
PcPAL-E484A-F	5’-TCAGTCCGCAgctCAGCACAACCAAG -3’
PcPAL-E484G-F	5’-TCAGTCCGCAggtCAGCACAACCAAG -3’
PcPAL-E484V-F	5’-TCAGTCCGCAgttCAGCACAACCAAG -3’
PcPAL-E484X-R	5’-ACGTGGTTGGTAACCGGG-3’

Table S2. X–ray crystallographic data of **4a**

Bond precision:	C-C = 0.0098 A		Wavelength=1.54184
Cell:	a=8.24139(12) b=5.38734(7)	c=14.6796(2)	
	alpha=90 beta=99.7698(13)	gamma=90	
Temperature:	300 K		
	Calculated		Reported
Volume	642.310(16)		642.312(15)
Space group	P 21		P 1 21 1
Hall group	P 2yb		P 2yb
Moiety formula	C10 H12 I N O2, 2(H2 O)		C10 H12 I N O2, 2(H2 O)
Sum formula	C10 H16 I N O4		C10 H16 I N O4
Mr	341.14		341.14
Dx,g cm-3	1.764		1.764
Z	2		2
Mu (mm-1)	19.616		19.616
F000	336.0		336.0
F000'	336.43		
h,k,lmax	10,6,18		10,6,18
Nref	2695[1497]		2522
Tmin,Tmax	0.180,0.208		0.191,1.000
Tmin'	0.063		
Correction method= # Reported T Limits: Tmin=0.191 Tmax=1.000 AbsCorr = MULTI-SCAN			
Data completeness= 1.68/0.94		Theta(max)= 76.197	
R(reflections)= 0.0457 (2434)			wR2(reflections)= 0.1333(2522)
S = 1.104		Npar= 147	

Table S3. X–ray crystallographic data of **5a**

Bond precision:	C-C = 0.0032 A		Wavelength=1.54184
Cell:	a=7.5448(3) b=5.90884(19)	c=12.7539(5)	

	alpha=90 beta=100.056(4) gamma=90	
Temperature: 292 K		
	Calculated	Reported
Volume	559.85(4)	559.84(4)
Space group	P 21	P 1 21 1
Hall group	P 2yb	P 2yb
Moiety formula	C10 H12 N2 O4, H2 O	C10 H12 N2 O4, H2 O
Sum formula	C10 H14 N2 O5	C10 H14 N2 O5
Mr	242.23	242.23
Dx,g cm-3	1.437	1.437
Z	2	2
Mu (mm-1)	0.991	0.991
F000	256.0	256.0
F000'	256.93	
h,k,lmax	9,7,16	9,7,16
Nref	2330[1283]	2175
Tmin,Tmax	0.899,0.924	0.709,1.000
Tmin'	0.888	

Correction method= # Reported T Limits: Tmin=0.709 Tmax=1.000 AbsCorr = MULTI-SCAN

Data completeness= 1.70/0.93 Theta(max)= 75.954

R(reflections)= 0.0356(2052) wR2(reflections)= 0.1001(2175)

S = 1.101 Npar= 162

Table S4. X–ray crystallographic data of **8a**

Bond precision:	C-C = 0.0033 A		Wavelength=1.54184
Cell:	a=13.7630(4)	b=5.5065(2)	c=14.2159(4)
	alpha=90	beta=102.629(3)	gamma=90
Temperature:	300 K		

	Calculated	Reported
Volume	1051.30(6)	1051.30(6)
Space group	P 21	P 1 21 1
Hall group	P 2yb	P 2yb
Moiety formula	C10 H12 F N O2, H2 O	C10 H12 F N O2, H2 O
Sum formula	C10 H14 F N O3	C10 H14 F N O3
Mr	215.22	215.22
Dx,g cm-3	1.360	1.360
Z	4	4
Mu (mm-1)	0.948	0.948
F000	456.0	456.0
F000'	457.66	
h,k,lmax	17,6,17	17,6,17
Nref	4378[2427]	3738
Tmin,Tmax	0.903,0.927	0.665,1.000
Tmin'	0.892	

Correction method= # Reported T Limits: Tmin=0.665 Tmax=1.000
AbsCorr = MULTI-SCAN

Data completeness= 1.54/0.85 Theta(max)= 76.074

R(reflections)= 0.0329(3513) wR2(reflections)= 0.0876(3738)

S = 1.055 Npar= 281

Table S5. X–ray crystallographic data of **9a**

Bond precision:	C-C = 0.0027 A		Wavelength=1.54184
Cell:	a=5.8720(1)	b=6.4774(1)	c=16.5959(2)
	alpha=90	beta=90.612(1)	gamma=90
Temperature:	100 K		

	Calculated	Reported
Volume	631.194(16)	631.194(16)
Space group	P 21	P 1 21 1
Hall group	P 2yb	P 2yb
Moiety formula	C14 H15 N O2, H2 O	H2 O, C14 H15 N O2
Sum formula	C14 H17 N O3	C14 H17 N O3
Mr	247.29	247.28
Dx,g cm-3	1.301	1.301
Z	2	2
Mu (mm-1)	0.746	0.746
F000	264.0	264.0
F000'	264.83	
h,k,lmax		7,7,20
Nref		2428
Tmin,Tmax	0.851,0.956	0.805,1.000
Tmin'	0.742	

Correction method= # Reported T Limits: Tmin=0.805 Tmax=1.000
AbsCorr = MULTI-SCAN

Data completeness=	Theta(max)= 74.301
R(reflections)= 0.0272(2391)	wR2(reflections)= 0.0755(2428)
S = 1.040	Npar= 169

Table S6. Angle and distance statistics of key atoms after QM/MM optimized structures.

Enzyme-substrate complex	Cluster	d: MIO@N_βMeCA/CA@C2 (Å)	d: Tyr110@O_βMeCA/CA@C3 (Å)	d: Tyr110@H_βMeCA/CA@O2 (Å)	d: ARG345@CZ_βMeCA/CA@C1 (Å)	θ：Tyr110@O_Tyr110@H_βMeCA/CA@O2 (°)
(CA)/(PcPAL-WT)	1	3.5	3.13	3.28	4.06	122.05
(CA)/(PcPAL-WT)	2	3.6	3.07	2.88	4.38	129.07
(CA)/(PcPAL-WT)	3	3.68	3.01	3.39	3.98	129.5
(CA)/(PcPAL-WT)	4	3.69	3.04	3.27	4.28	136.3
(CA)/(PcPAL-WT)	5	3.48	2.99	2.93	4.44	133.82
(βMeCA)/(PcPAL-WT)	1	3.51	3.86	1.51	4.46	169.02
(βMeCA)/(PcPAL-WT)	2	3.48	3.48	1.58	4.5	164.03
(βMeCA)/(PcPAL-WT)	3	3.83	3.76	1.53	4.54	167.92
(βMeCA)/(PcPAL-WT)	4	3.73	3.73	1.52	4.46	165.38
(βMeCA)/(PcPAL-WT)	5	3.8	3.63	1.54	4.56	166.67
(βMeCA)/(PcPAL-L256V-I460V)	1	3.6	3.09	3.28	3.99	118.63
(βMeCA)/(PcPAL-L256V-I460V)	2	3.53	3.34	1.7	4.14	159.25
(βMeCA)/(PcPAL-L256V-I460V)	3	3.32	3.19	2.97	4.02	128.54
(βMeCA)/(PcPAL-L256V-I460V)	4	3.53	3.07	3.67	3.98	131.6
(βMeCA)/(PcPAL-L256V-I460V)	5	3.36	3.23	3.18	4.01	126.41

Supplementary Figures

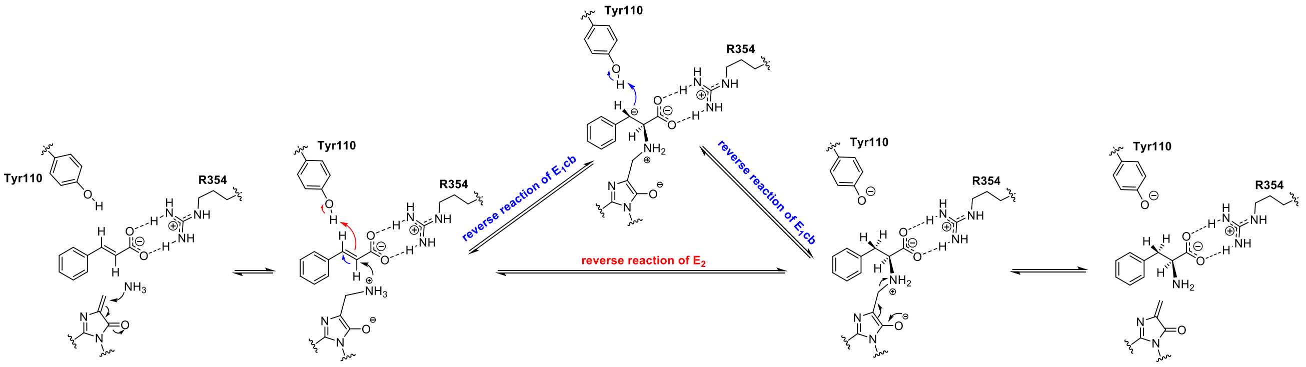

Figure S1. Proposed mechanism for the amination reaction mediated by PcPAL.

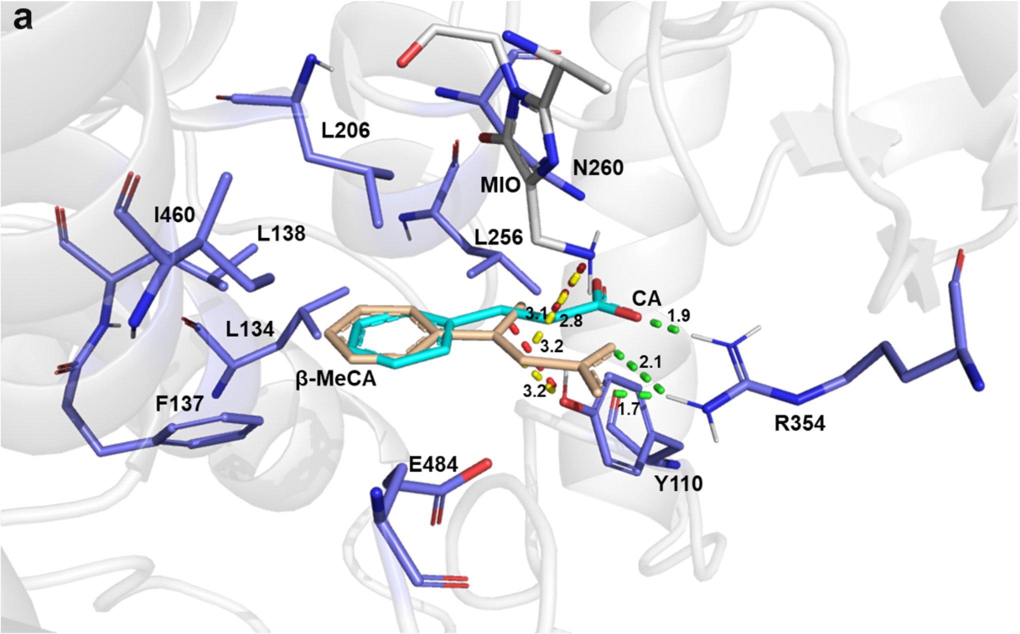

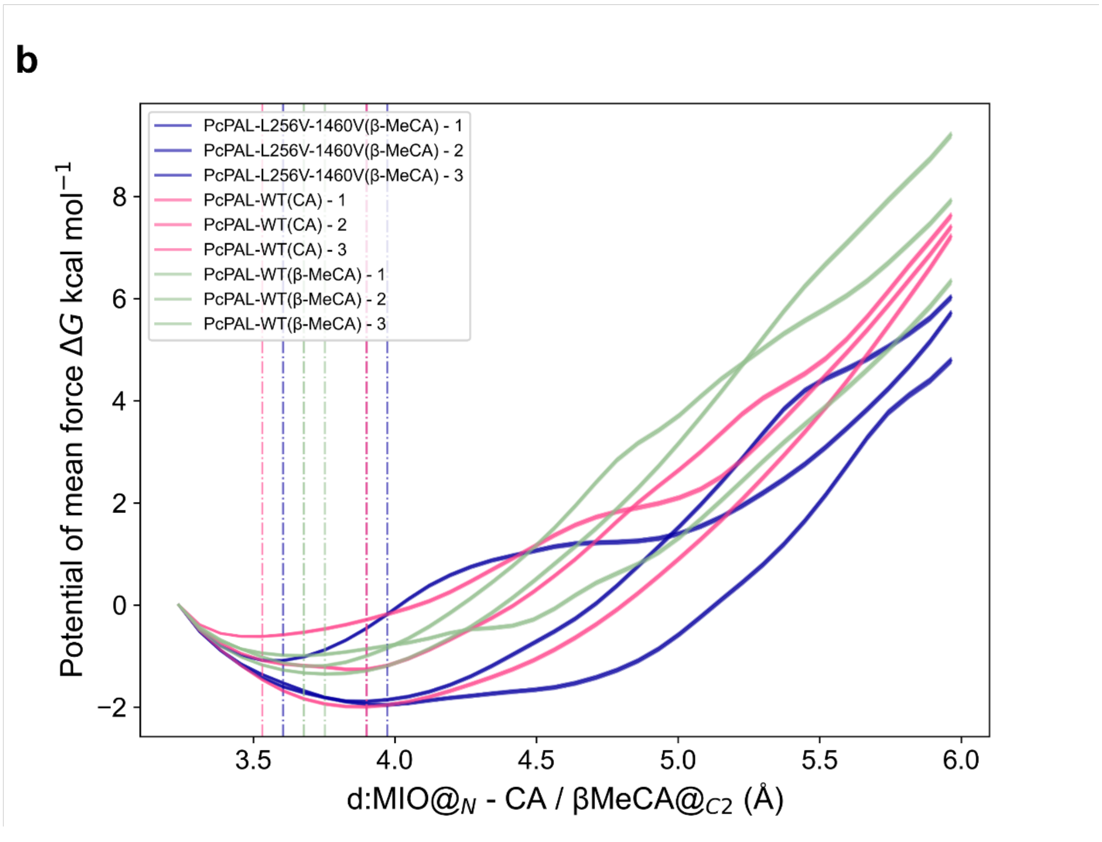

Figure S2. Docking results and the potentials of mean force results. **a**, Superposition of the docking result with CA (cyan) and β-MeCA (gray). **b**, Potentials of mean force as function of the distance from the NH_2 of MIO to αC of CA or β-MeCA. The results showed that, for substrate CA, the optimal amination distance ranged from 3.5 to 3.9 Å when it had the lowest energy conformations in *Pc*PAL-WT, whereas the corresponding distance ranged from 3.6 to 3.7 Å for β-MeCA.

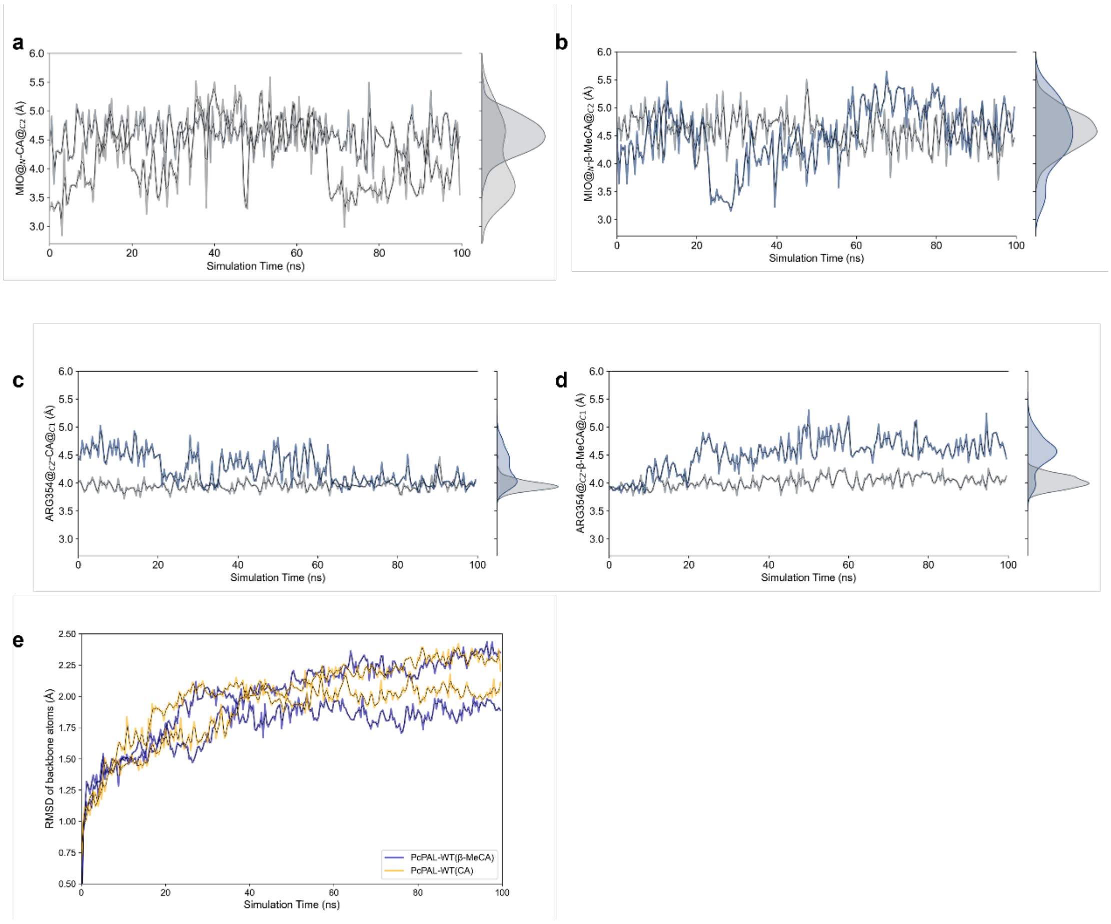

Figure S3. Computational results of cMD. **a**, **b,** the distance from atom N of MIO group to the αC of CA and β-MeCA respectively during cMD simulations. The distances from the N atom of the MIO group to the αC of CA and β-MeCA were both around 4.0 Å during cMD simulations. **c**, **d**, the distance between R354 and the carboxyl carbon of CA and β-MeCA respectively during cMD simulations. The results exhibited similar distances to the R354 residue around 4.2 Å. **e**, RMSD of the backbone atoms during cMD simulations. Similar results were observed when measuring the RMSD of backbone atoms (converging around 2.3 and 2.0 Å).

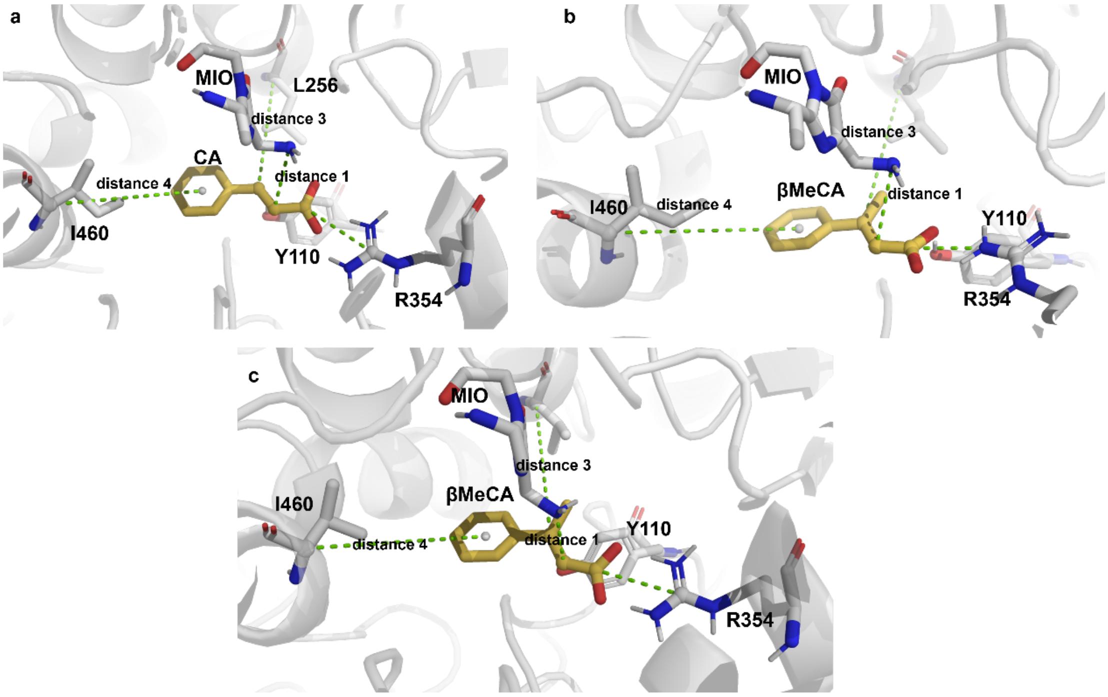

Figure S4. Statistical distances in the umbrella sampling (US) simulations. **a**, distance 1 from the amino group of MIO to the αC of CA, distance 3 from βC of CA to Leu256 and distance 4 from the benzene ring center of CA to Ile460 within the (CA)/(PcPAL-WT) complex. **b**, distance 1 from amino group of MIO to the αC of β-MeCA, distance 3 from βC of β-MeCA to Leu256 and distance 4 from the benzene ring center of β-MeCA to Ile460 within the (β-MeCA)/(PcPAL-WT) complex. **c**, distance1 from amino group of MIO to the αC of β-MeCA, distance 3 from βC of β-MeCA to Val256 and distance 4 from the benzene ring center of β-MeCA to Val460 within the (β-MeCA)/(PcPAL-L256V-IV460V) complex.

A

p1 [mM]	0.05	0.1	0.25	0.5	1
Average Peak Area [mAU]	660867	1491509	4617663	10033292	21106863
SD	12.35583533	11.77568116	4.966554809	9.899494937	5.715476066

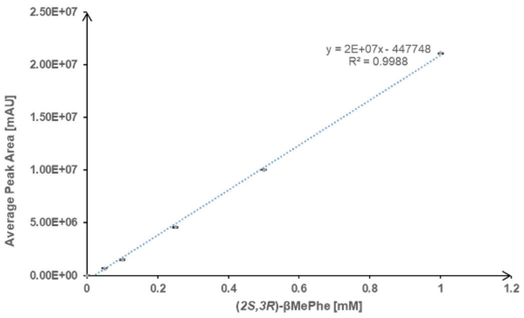

B

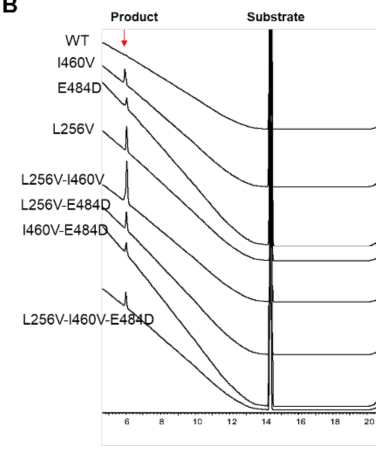

Figure S5. A) Standard curve of (*2S,3R*)-2-amino-3-phenyl-butyric acid (1a) and B) *in vitro* amination assays of different variants against β-methyl cinnamic acid.

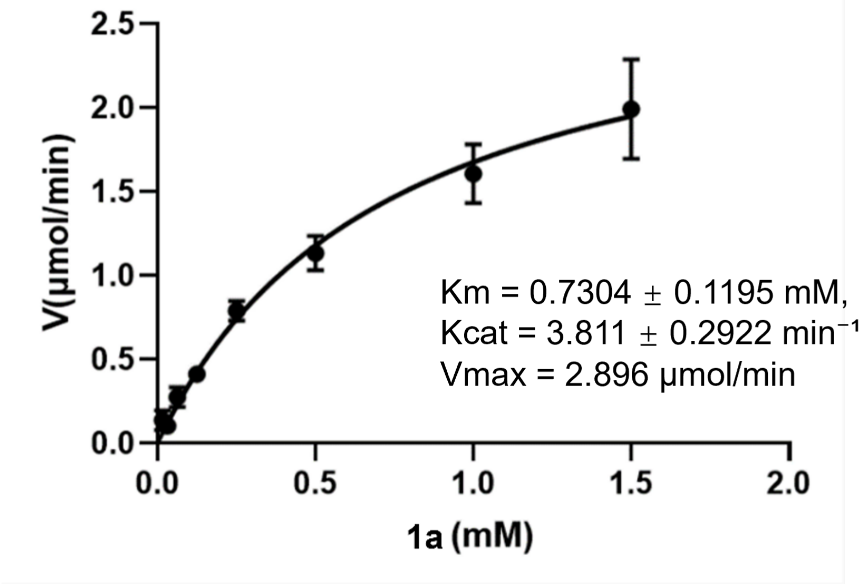

Figure S6. Michaelis-Menten curves for the ammonia elimination from (2*S*,3*R*)-β-MePhe (1a) catalyzed by PcPAL-L256V-I460V; measured in triplicate.

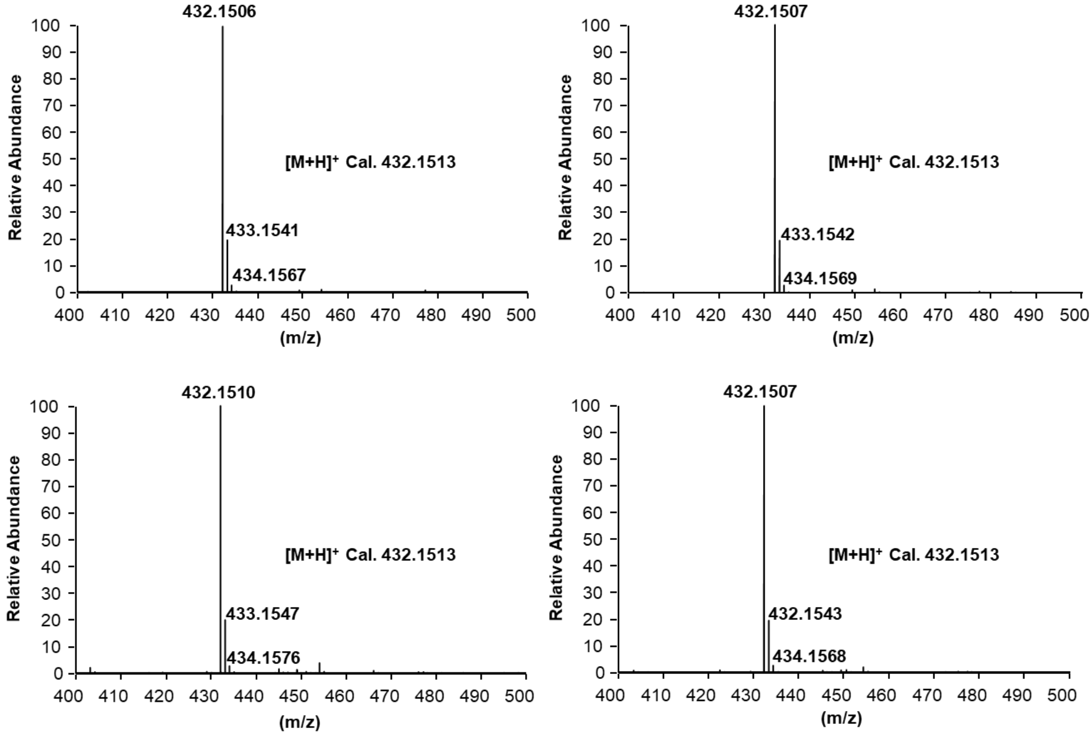

Figure S7. LC-HRMS data of Marfey's reagent derivatization products of β-MePhe. A) (*2S,3S*)-β-MePhe derivatized with Marfey's reagent; B) (*2S,3R*)-β-MePhe derivatized with Marfey's reagent; C) derivation of *in vitro* amination reaction product produced by PcPAL-L256V-I460V mutant; D) (*2R,3R*)-β-MePhe with Marfey's reagent.

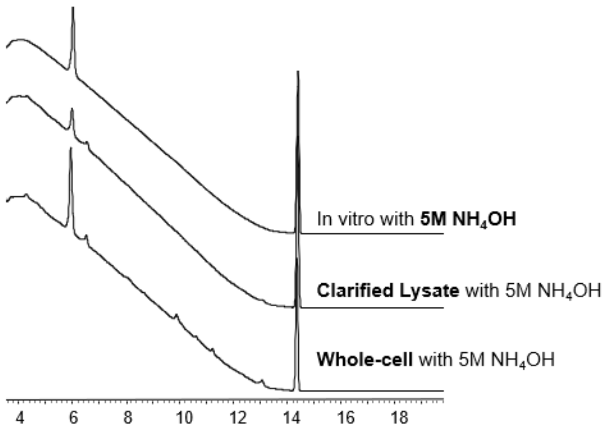

Figure S8. Optimization of the *in vivo* amination reaction conditions.

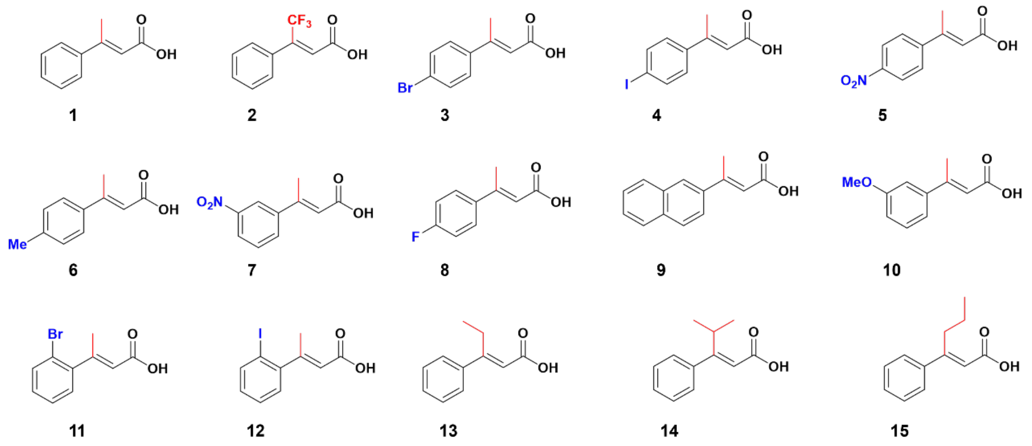

Figure S9. Overview of the chemically synthesized substrates for this study.

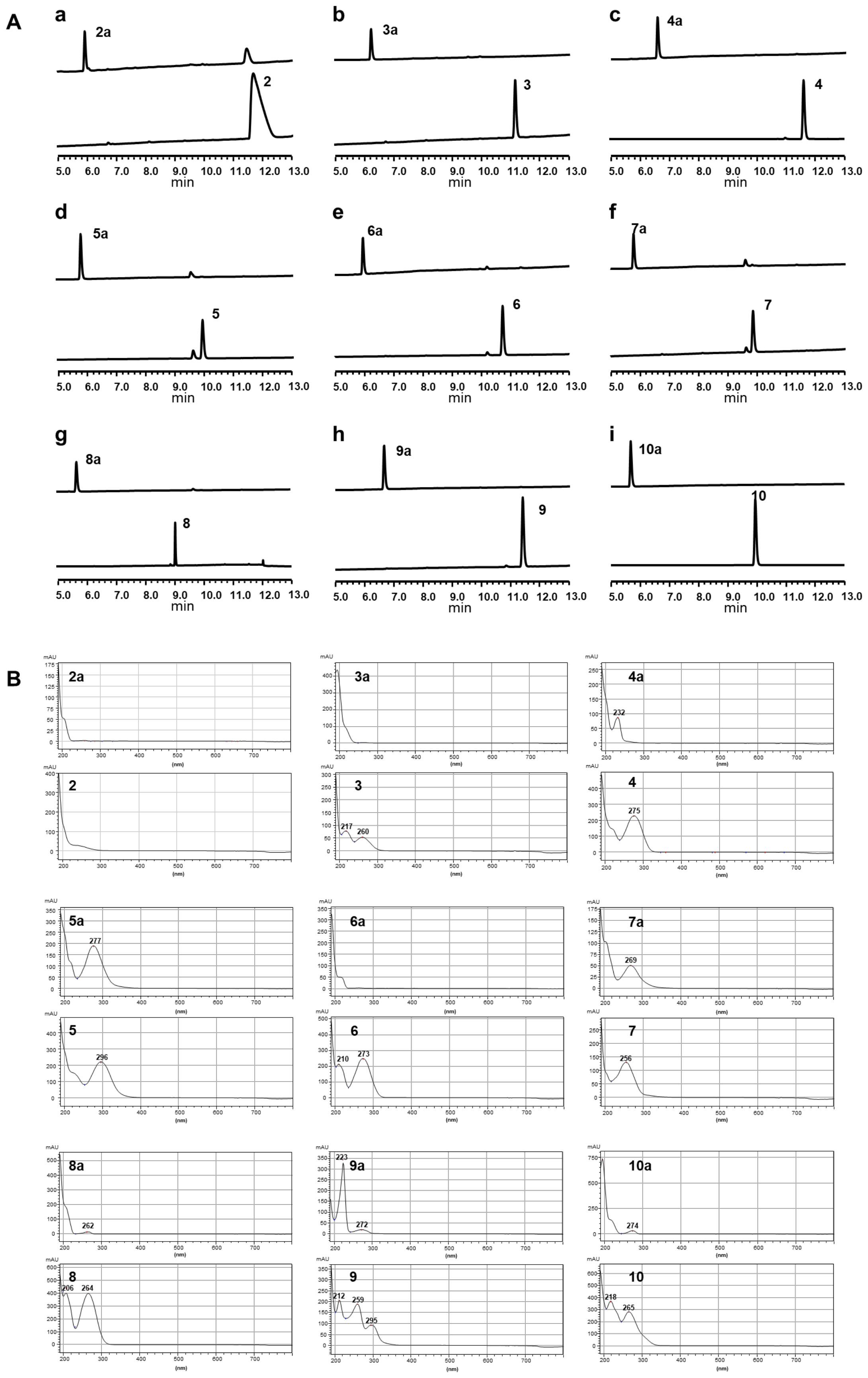

Figure S10. HPLC analysis of *in vitro* assays of PcPAL mutants against different substrates. **A**: a) **2** catalyzed by PcPAL-L256V-I460V; b) **3** catalyzed by PcPAL-

F137V-L256V-I460V; c) **4** catalyzed by PcPAL-L256V-I460V; d) **5** catalyzed by PcPAL-L256V-I460V; e) **6** catalyzed by PcPAL-L256V-I460V; f) **7** catalyzed by PcPAL-L256V-I460V; g) **8** catalyzed by PcPAL-L256V-I460V; h) **9** catalyzed by PcPAL-F137V-L256V-I460V; i) **10** catalyzed by PcPAL-F137V-L256V-I460V. **B**: UV- absorption spectra of the substrates and products.

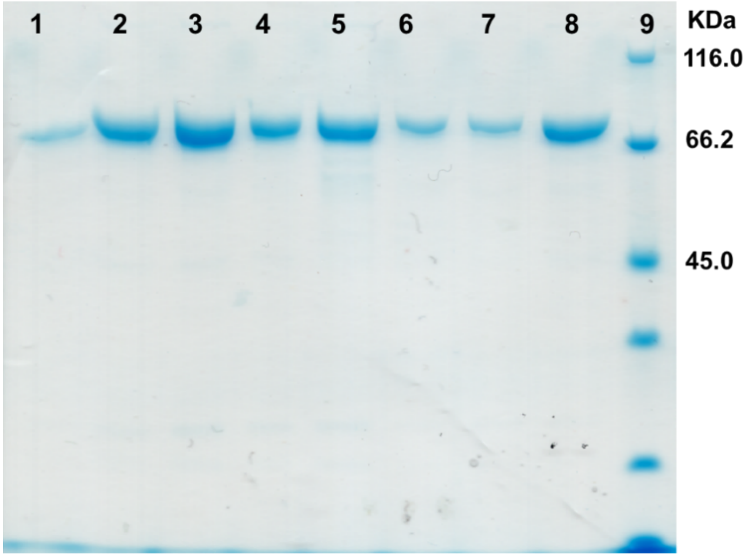

Figure S11. 12% SDS-PAGE analysis of the purified PcPALs. Lane **1**: PcPAL-E484D, 77.8 kDa; Lane **2**: PcPAL-I460V, 77.8 kDa; Lane **3**: PcPAL-L256V, 77.8 kDa; Lane **4**: PcPAL-L256V-I460V, 77.8 kDa; Lane **5**: PcPAL-L256V-E484D, 77.8 kDa; Lane **6**: PcPAL-E484D-I460V, 77.8 kDa; Lane **7**: PcPAL-L256V-I460V-E484D, 77.8 kDa; Lane **8**: PcPAL-F137V-L256V-I460V, 77.8 kDa; Lane **9**: Protein ladder.

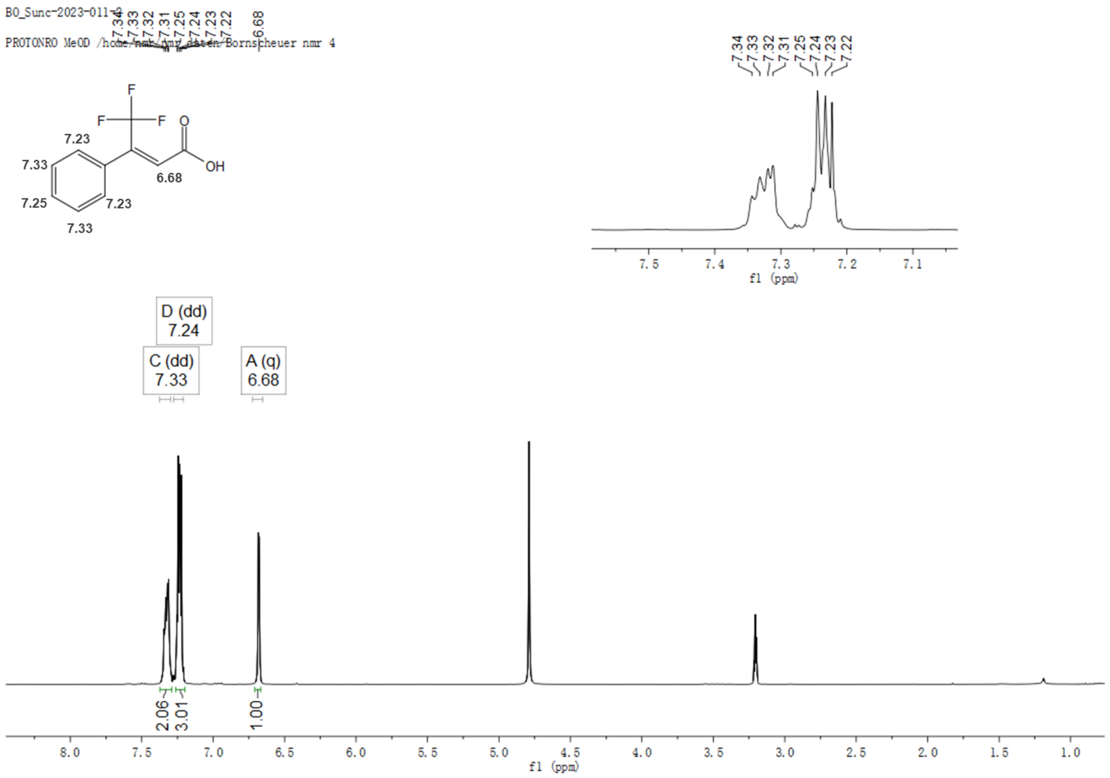

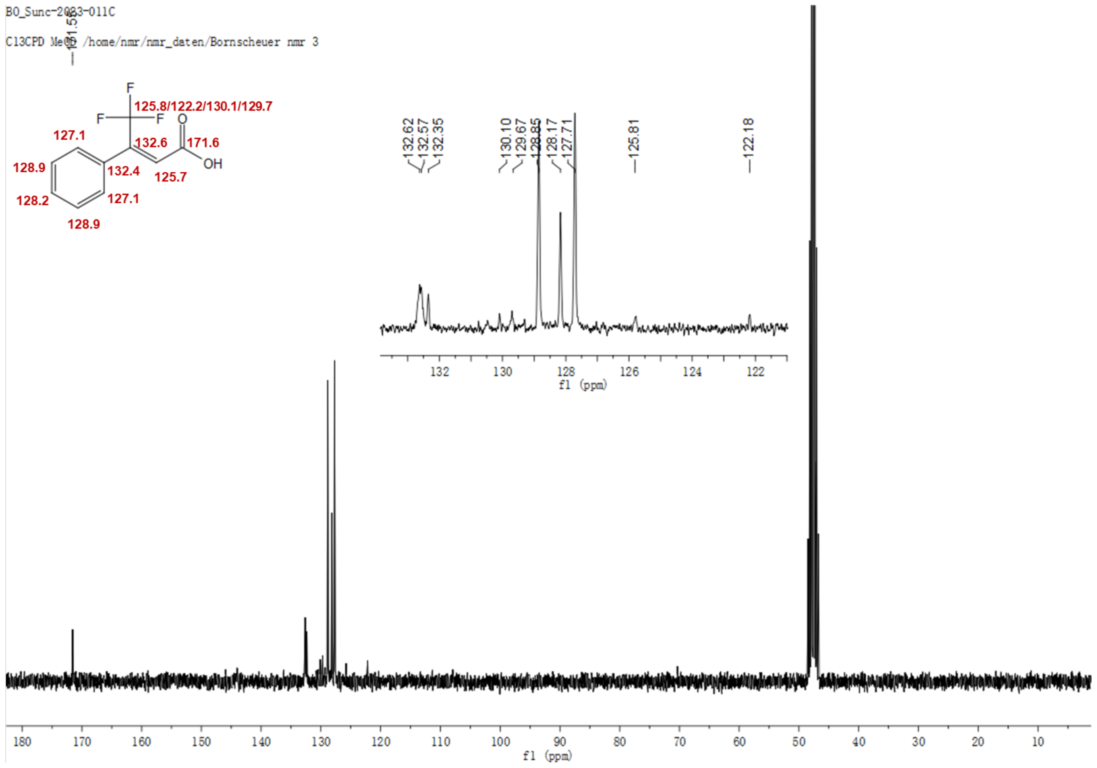

Figure S12. NMR spectra of **2**. ^{1}H-NMR (300 MHz, MeOD-d4); ^{13}C-NMR (75 MHz, MeOD-d4).

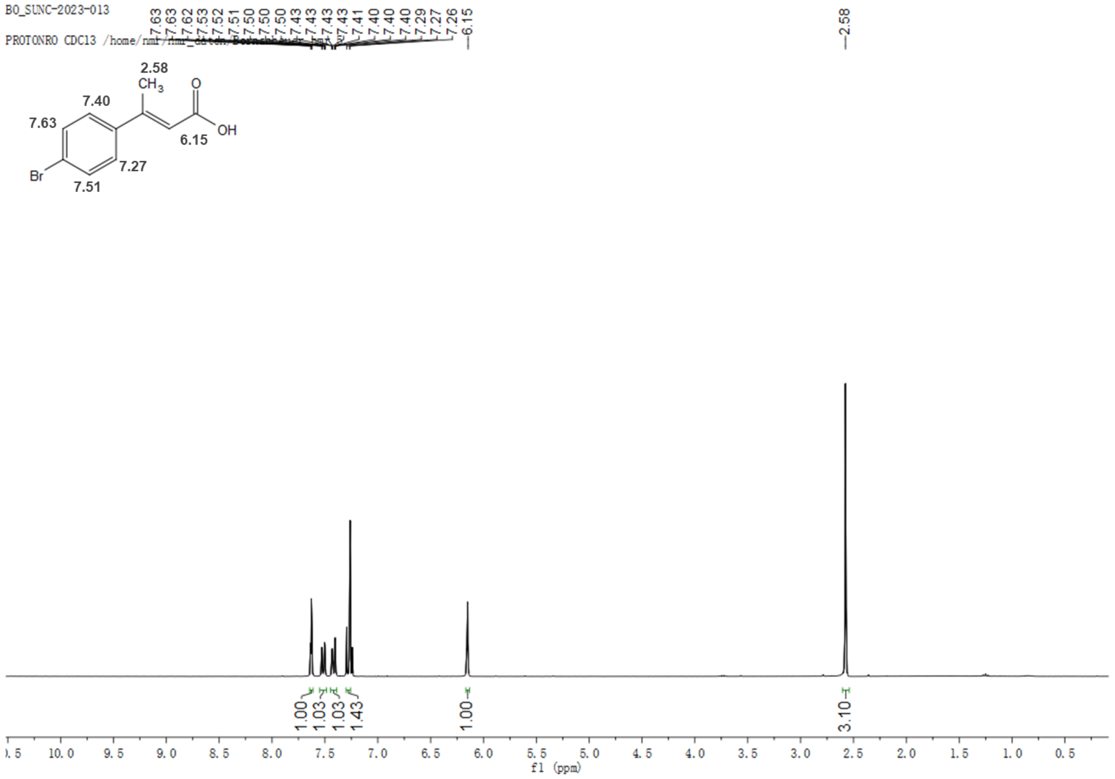

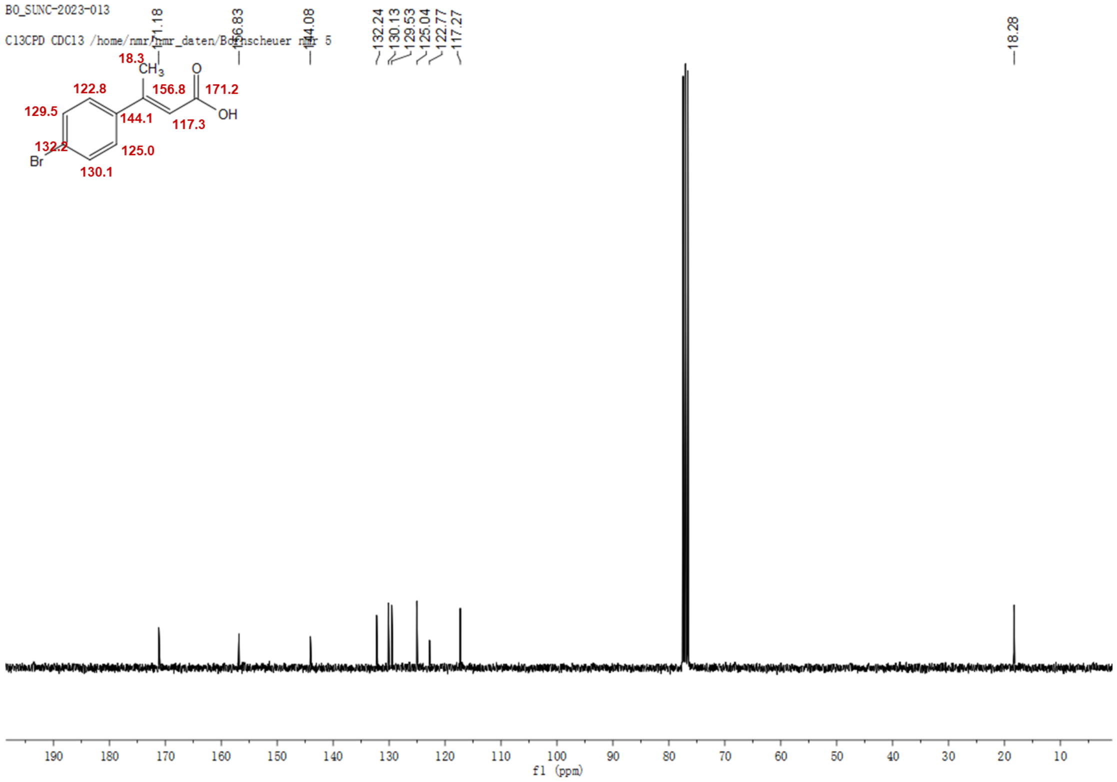

Figure S13. NMR spectra of **3**. ^{1}H-NMR (300 MHz, $CDCl_3$); ^{13}C-NMR (75 MHz, $CDCl_3$).

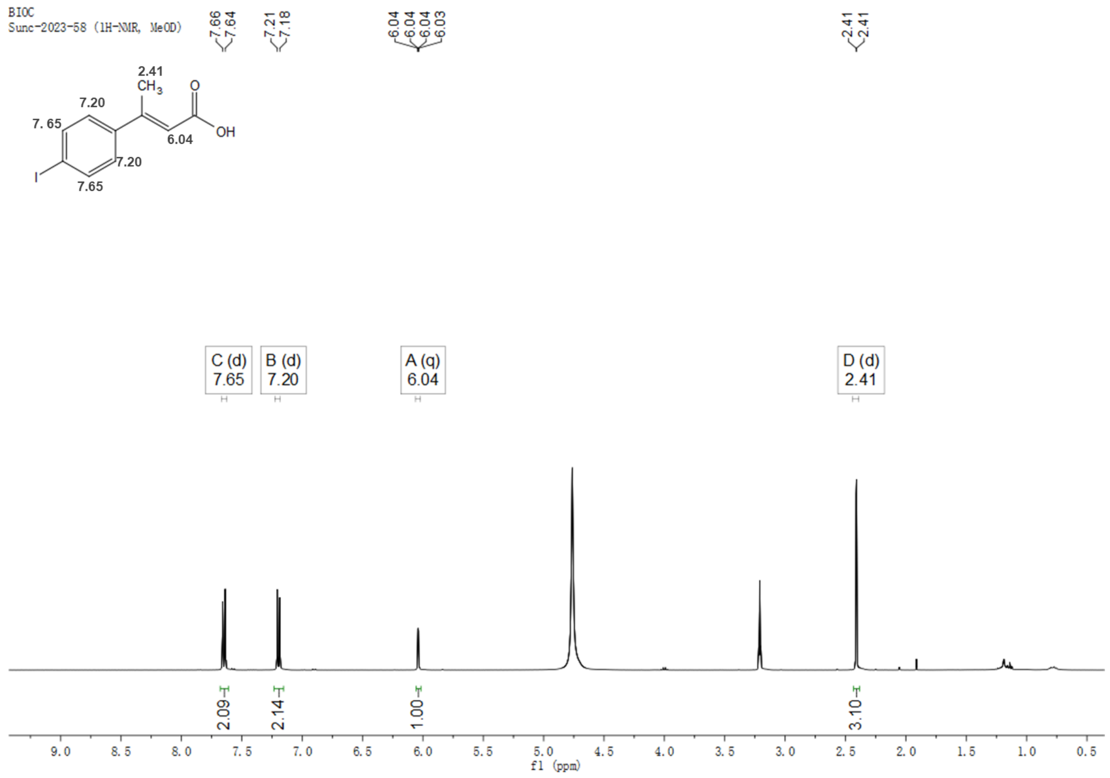

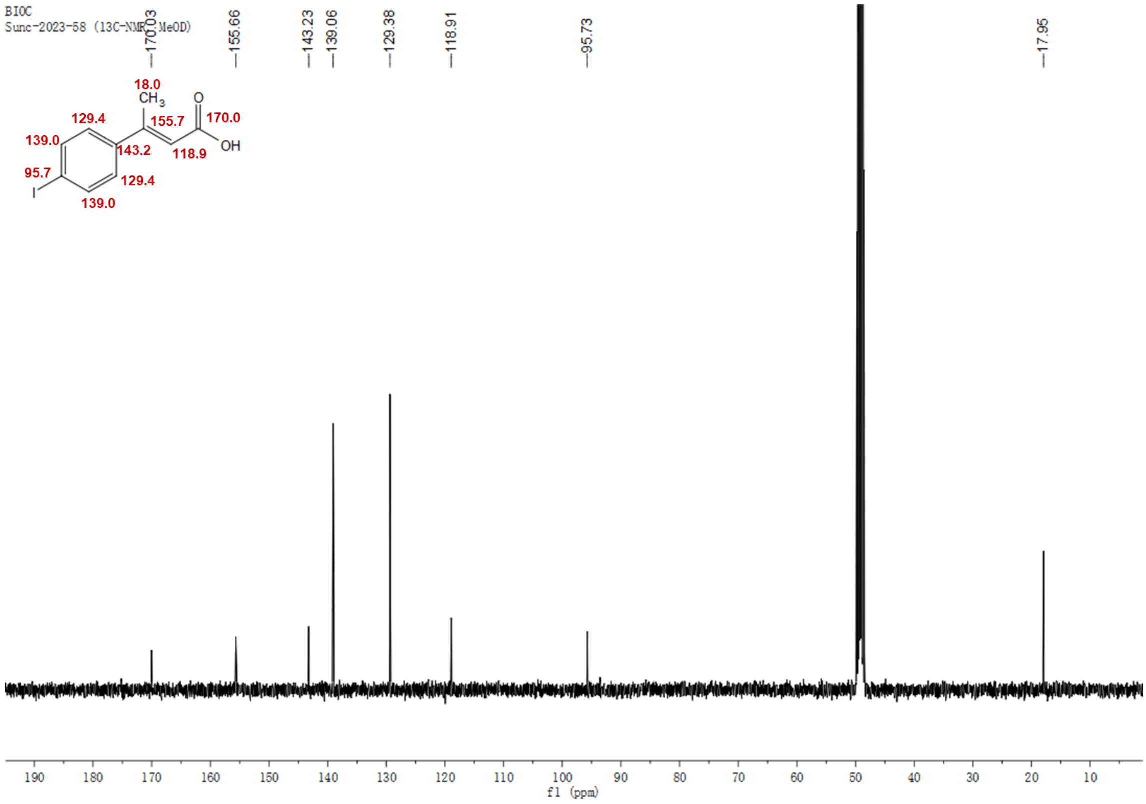

Figure S14. NMR spectra of **4**. ^{1}H-NMR (400 MHz, MeOD-d4); ^{13}C-NMR (101 MHz, MeOD-d4).

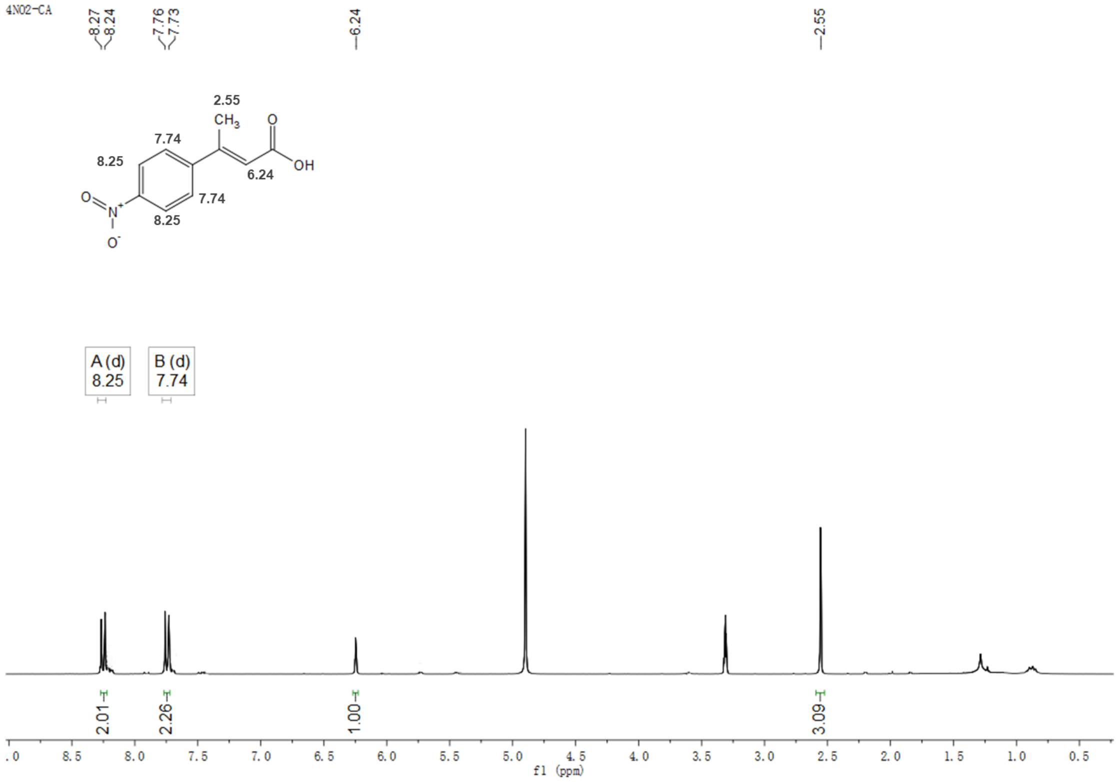

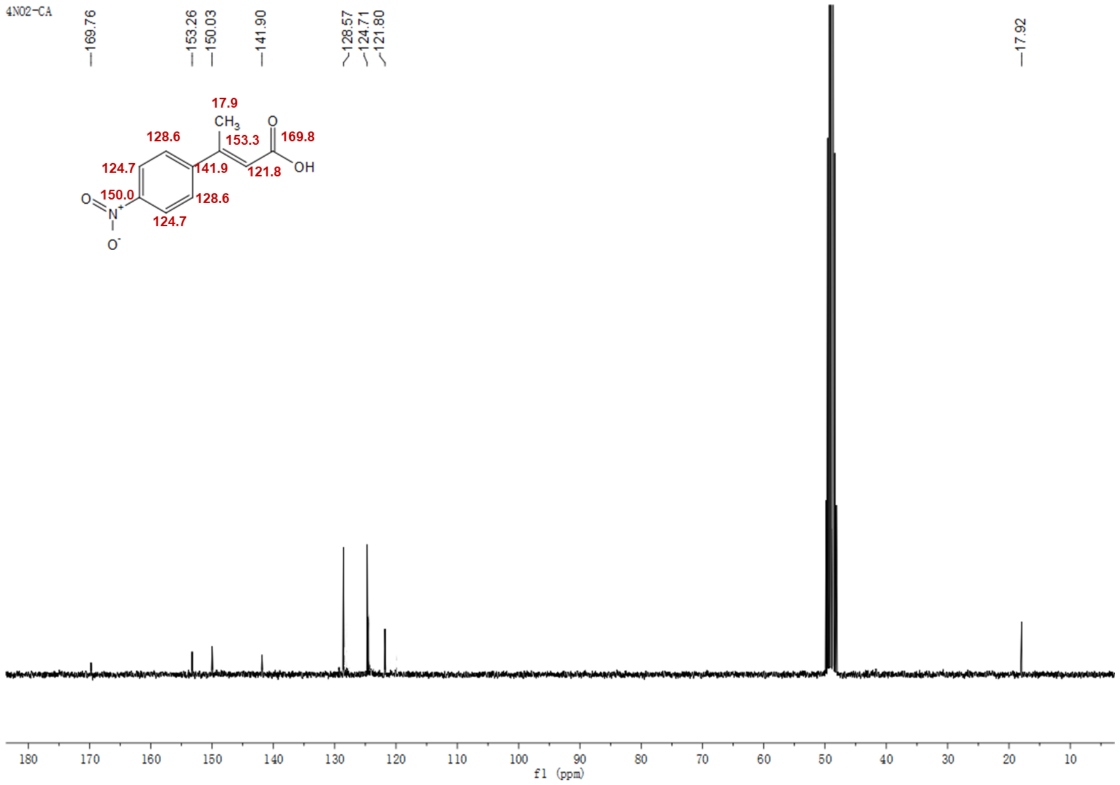

Figure S15. NMR spectra of **5**. ^{1}H-NMR (400 MHz, MeOD-d4); ^{13}C-NMR (101 MHz, MeOD-d4).

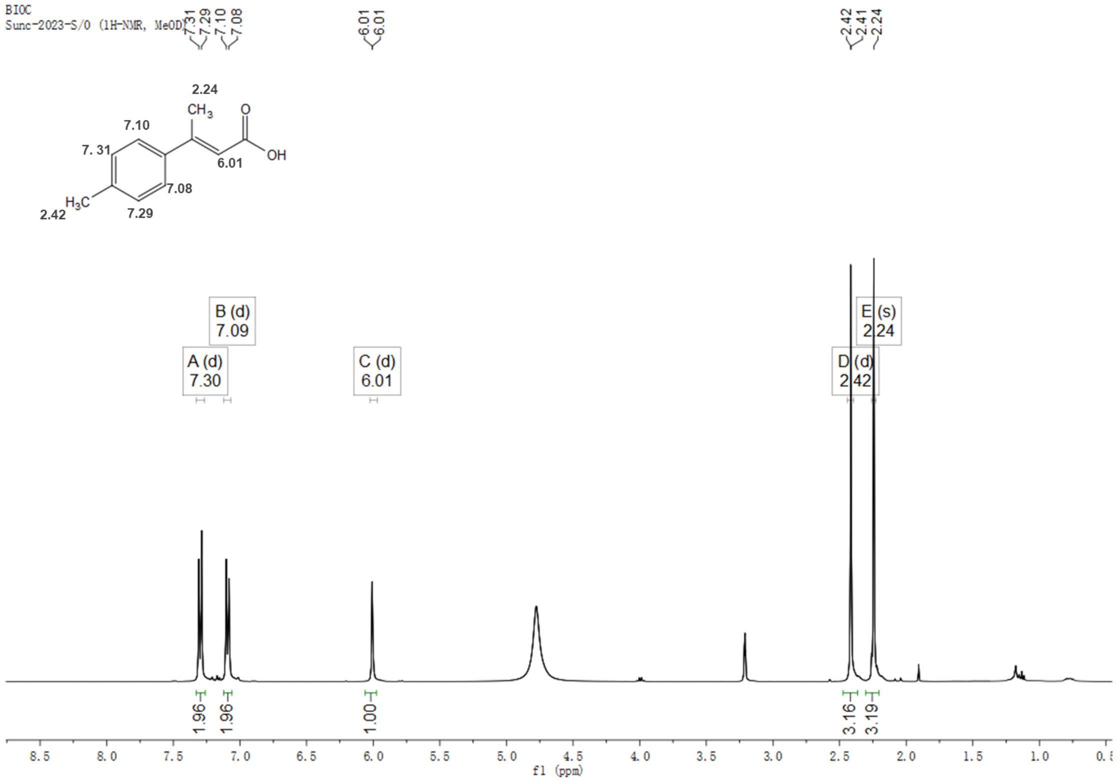

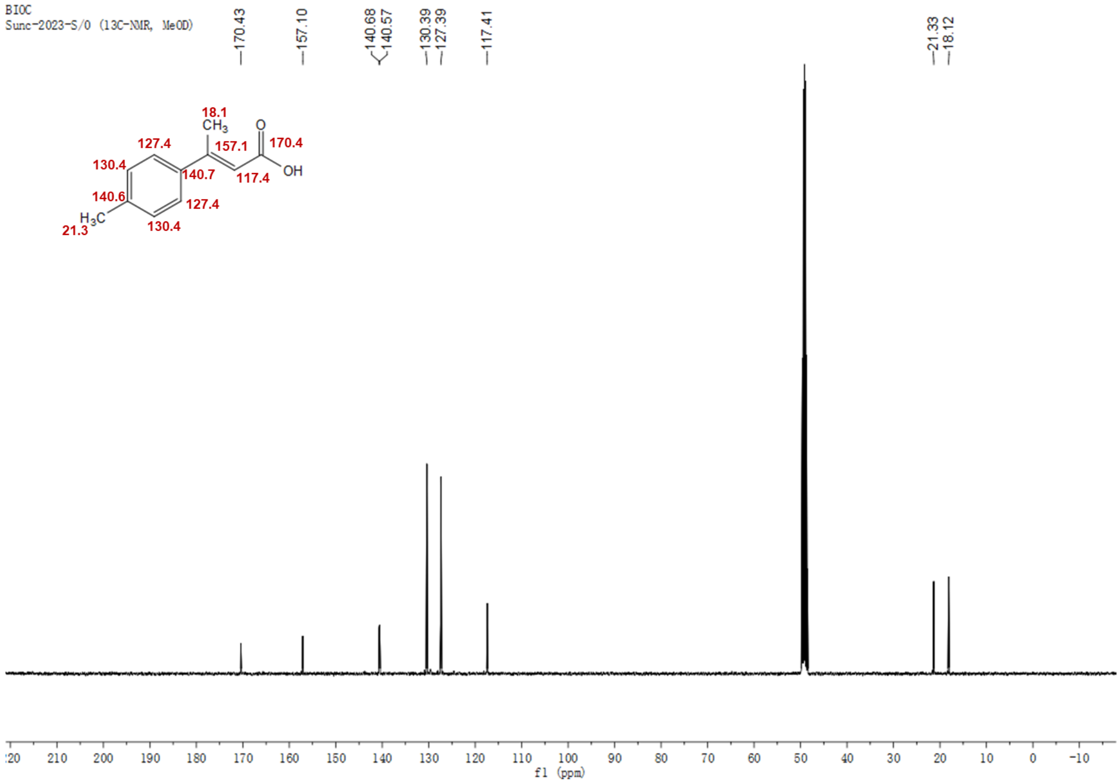

Figure S16. NMR spectra of **6**. ^{1}H-NMR (400 MHz, MeOD-d4); ^{13}C-NMR (101 MHz, MeOD-d4).

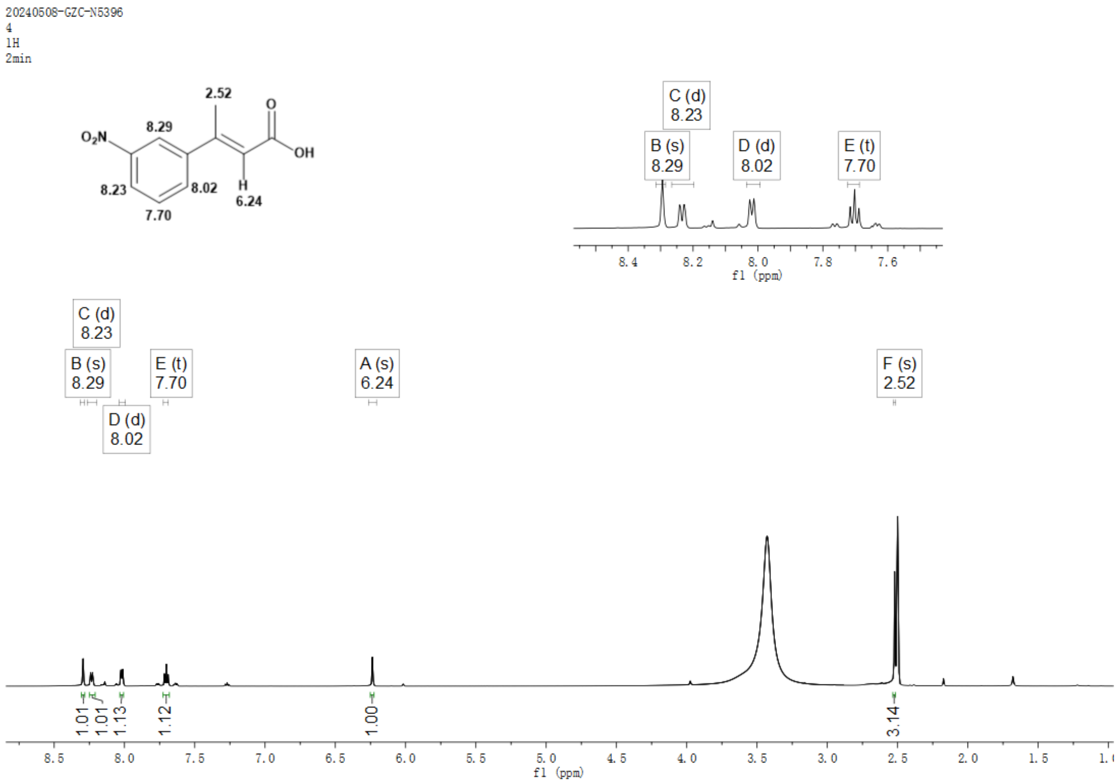

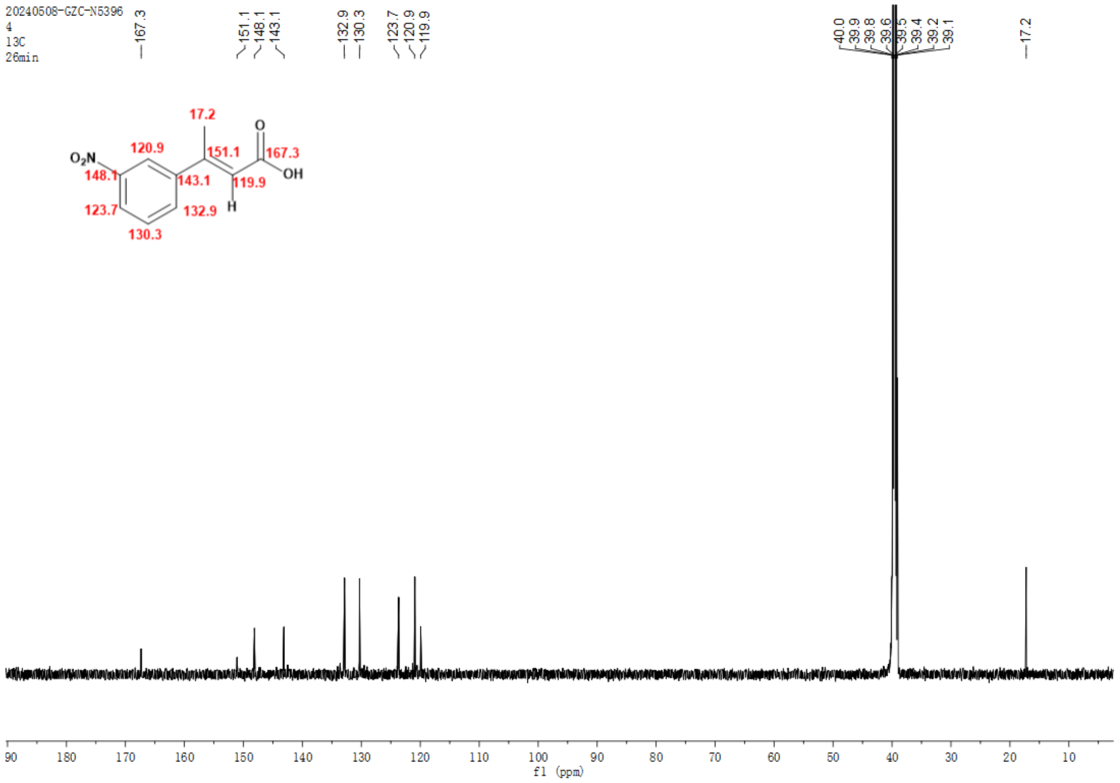

Figure S17. NMR spectra of **7**. ^{1}H-NMR (600 MHz, MeOD-d4); ^{13}C-NMR (151 MHz, MeOD-d4).

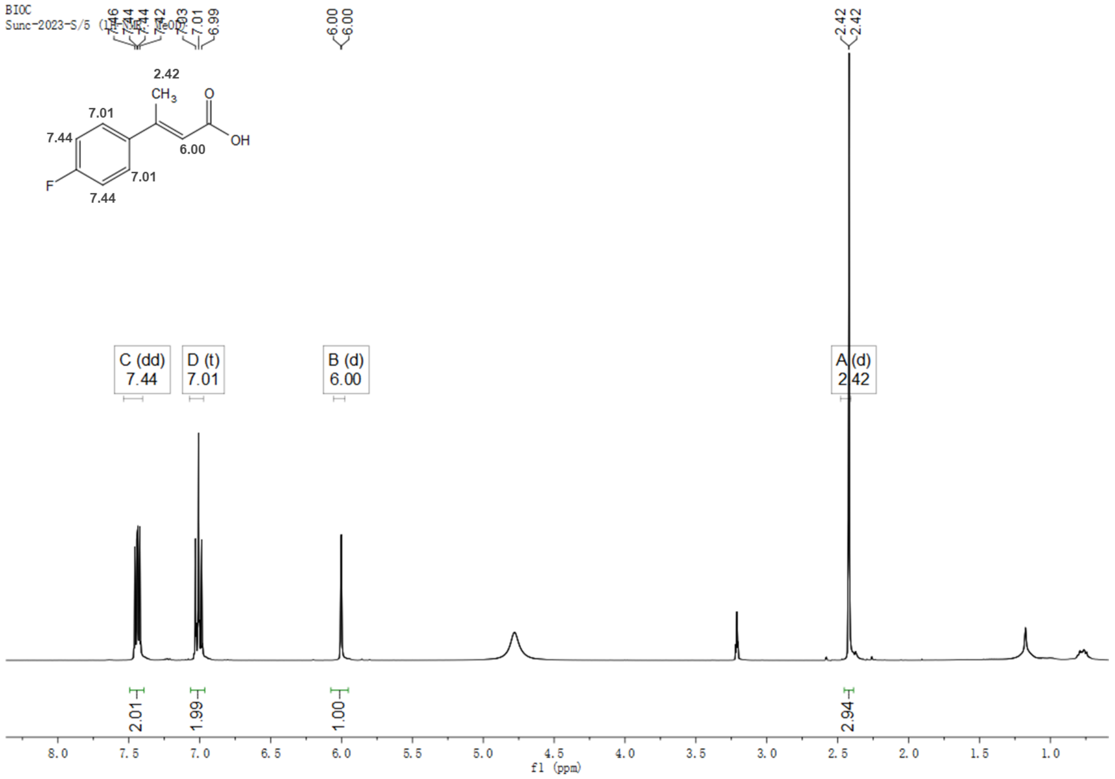

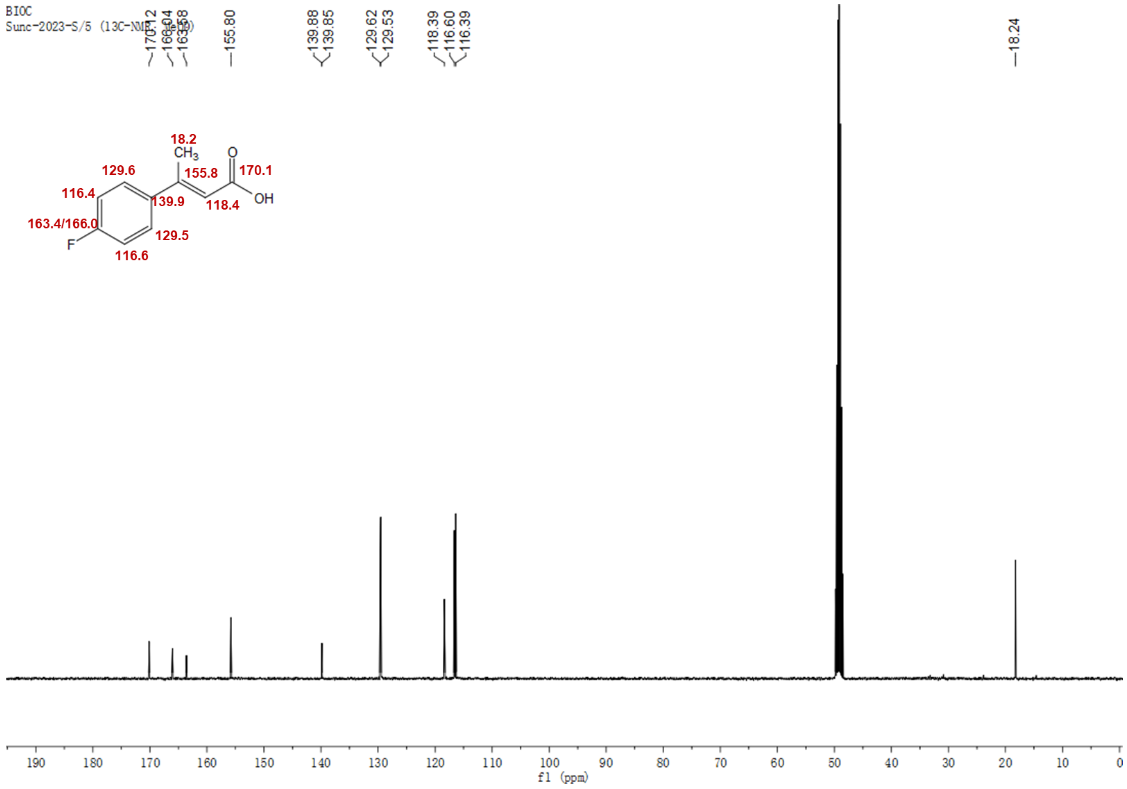

Figure S18. NMR spectra of **8**. ^{1}H-NMR (400 MHz, MeOD-d4); ^{13}C-NMR (101 MHz, MeOD-d4).

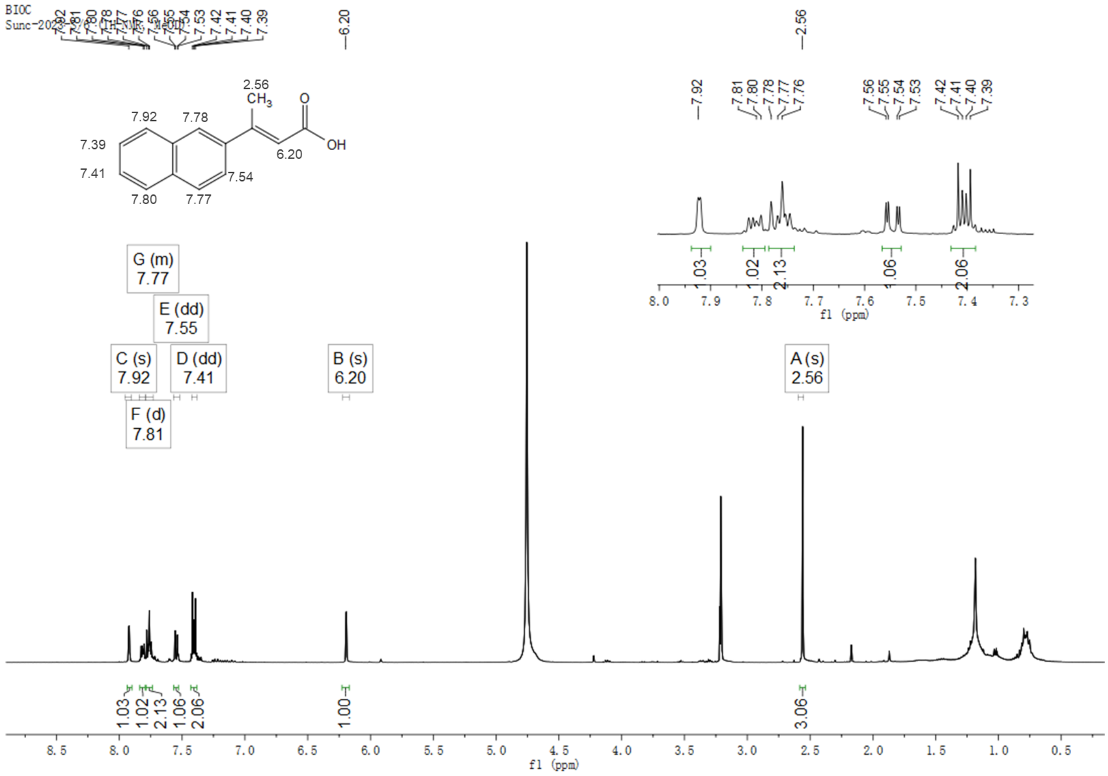

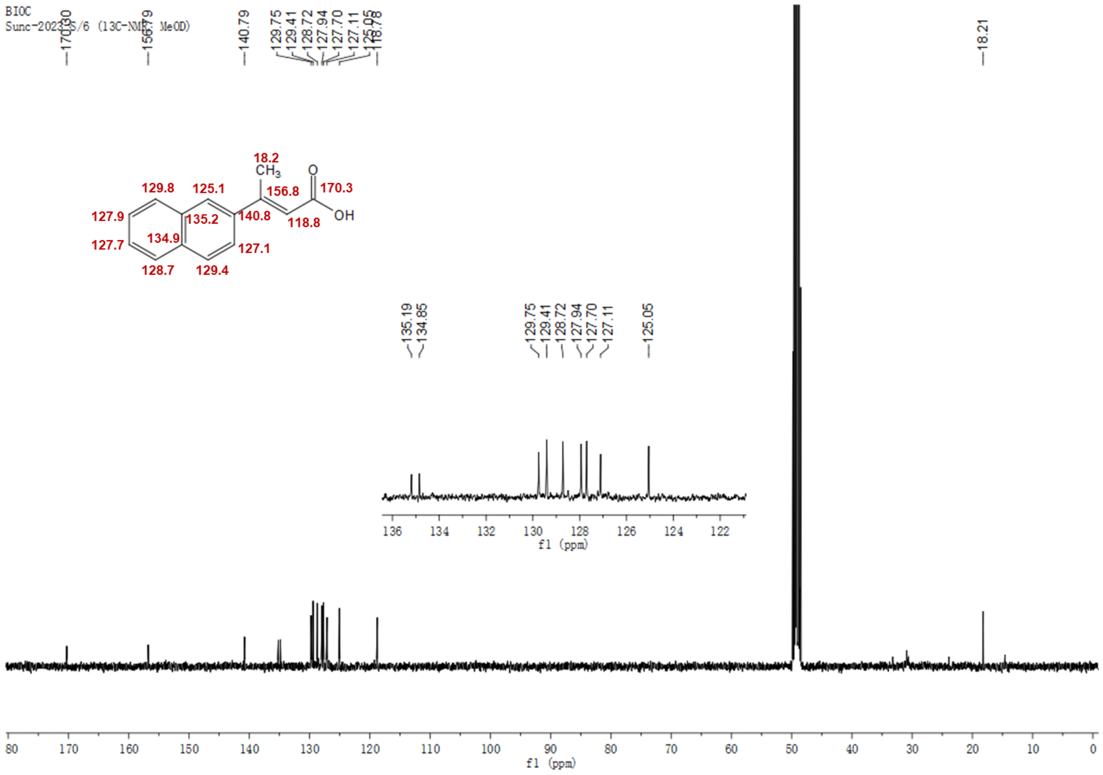

Figure S19. NMR spectra of **9**. ^{1}H-NMR (400 MHz, MeOD-d4); ^{13}C-NMR (101 MHz, MeOD-d4).

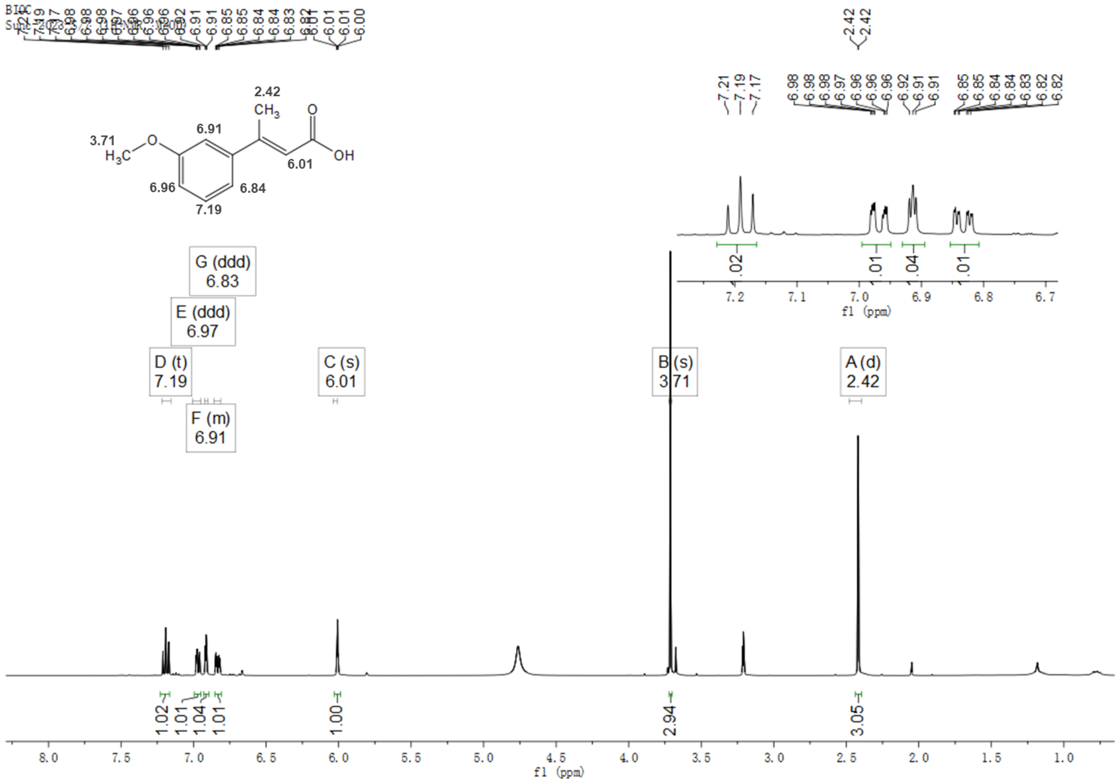

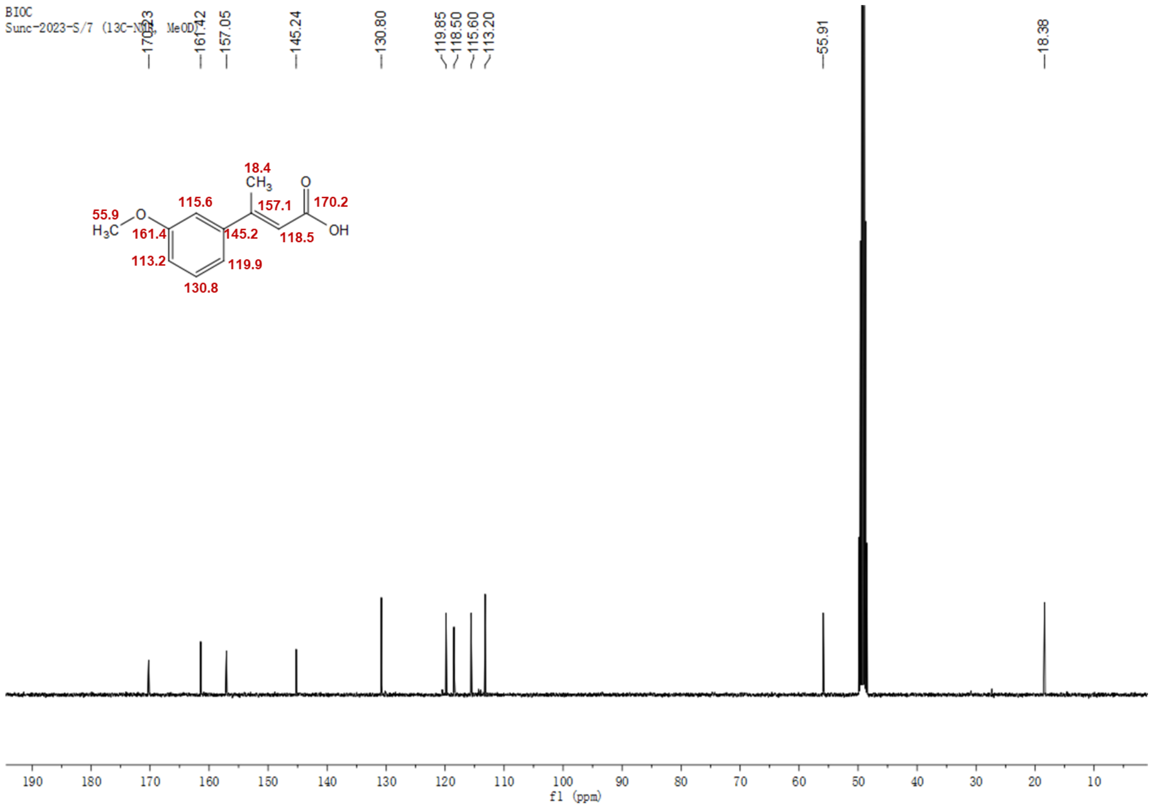

Figure S20. NMR spectra of **10**. ^{1}H-NMR (400 MHz, MeOD-d4); ^{13}C-NMR (101 MHz, MeOD-d4).

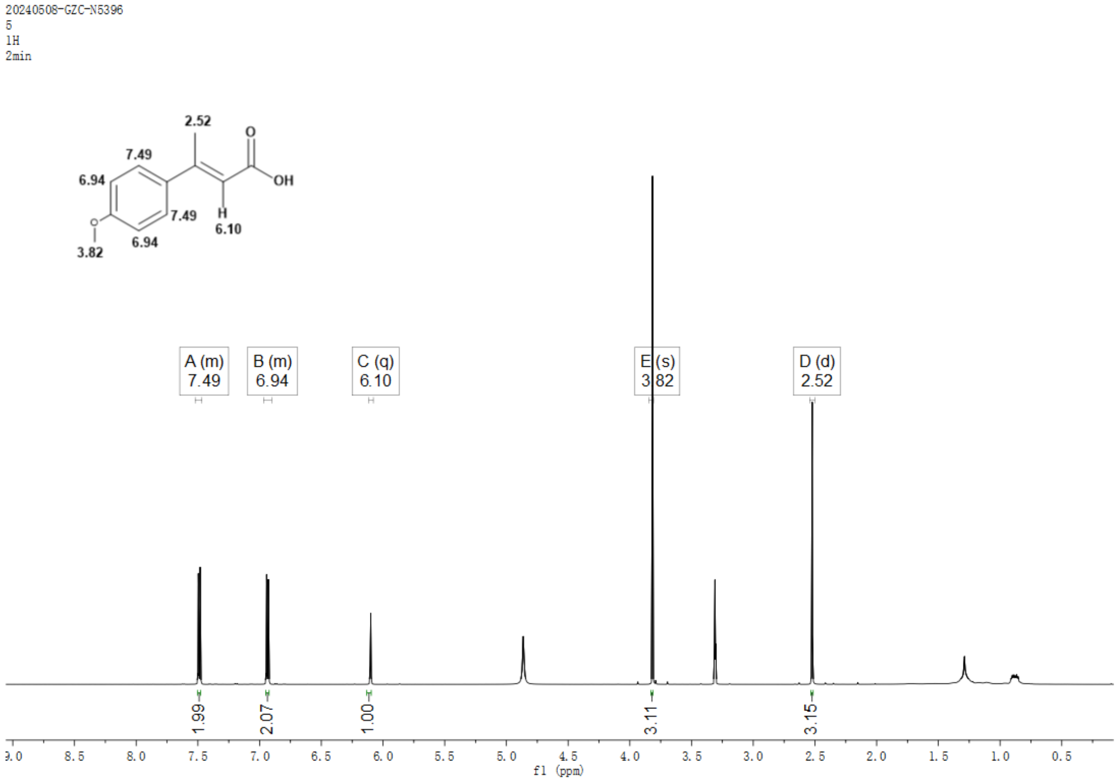

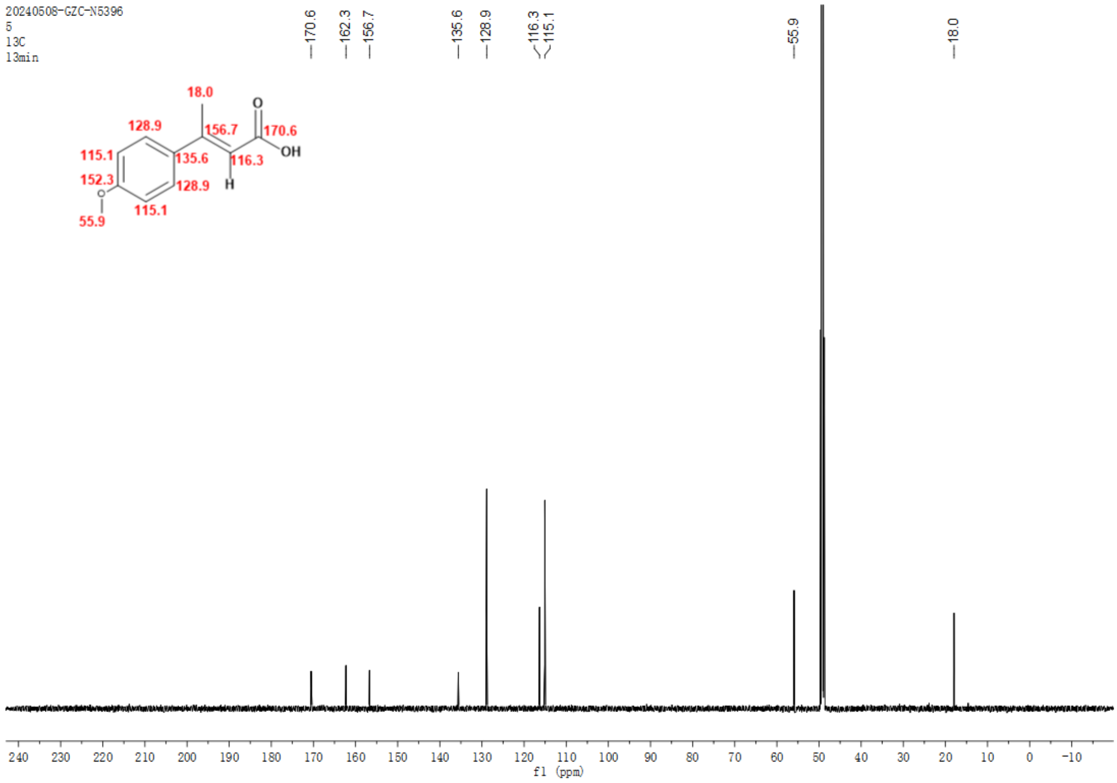

Figure S21. NMR spectra of **11**. ^{1}H-NMR (600 MHz, MeOD-d4); ^{13}C-NMR (151 MHz, MeOD-d4).

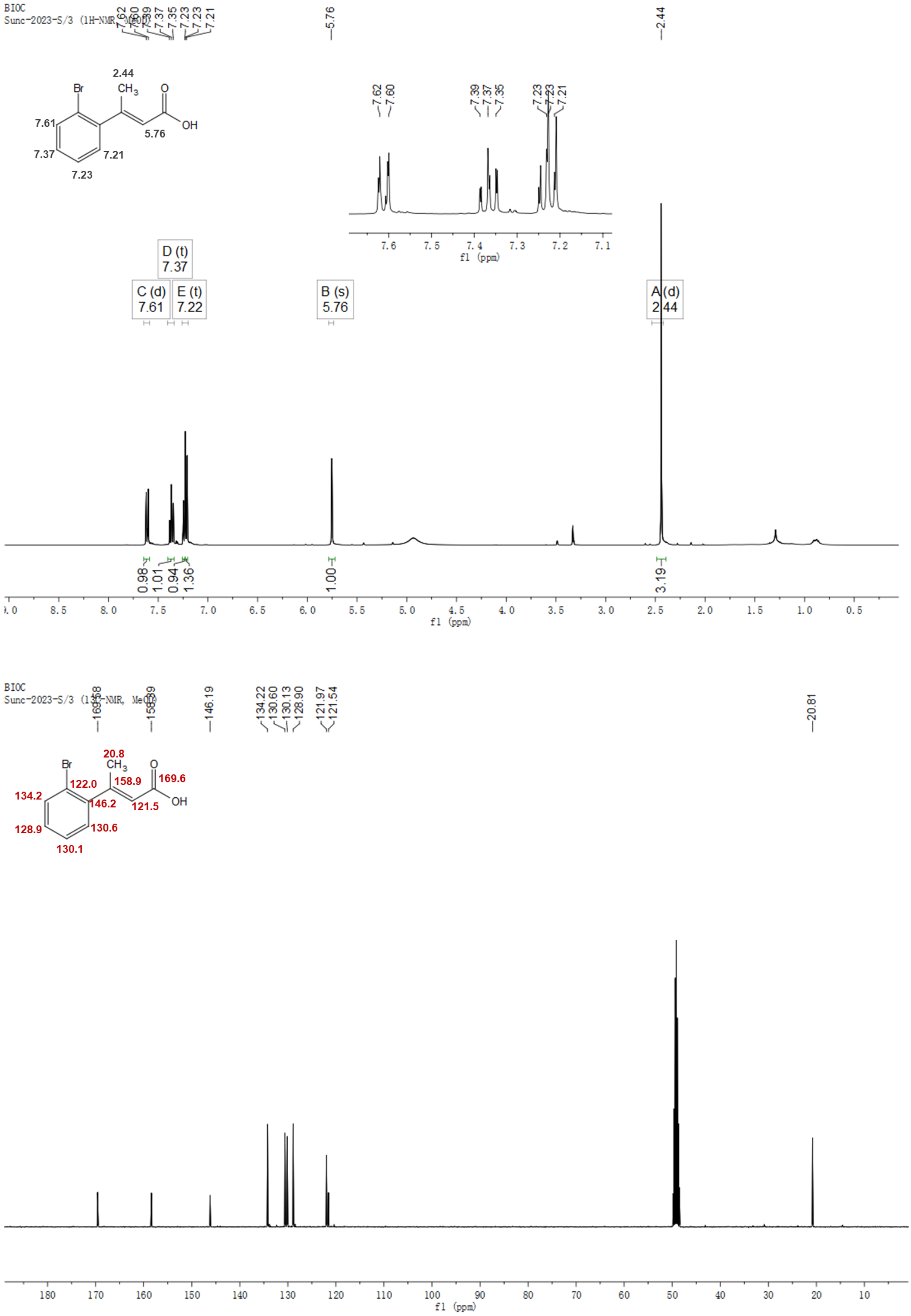

Figure S22. NMR spectra of **12**. ^{1}H-NMR (400 MHz, MeOD-d4); ^{13}C-NMR (101 MHz, MeOD-d4).

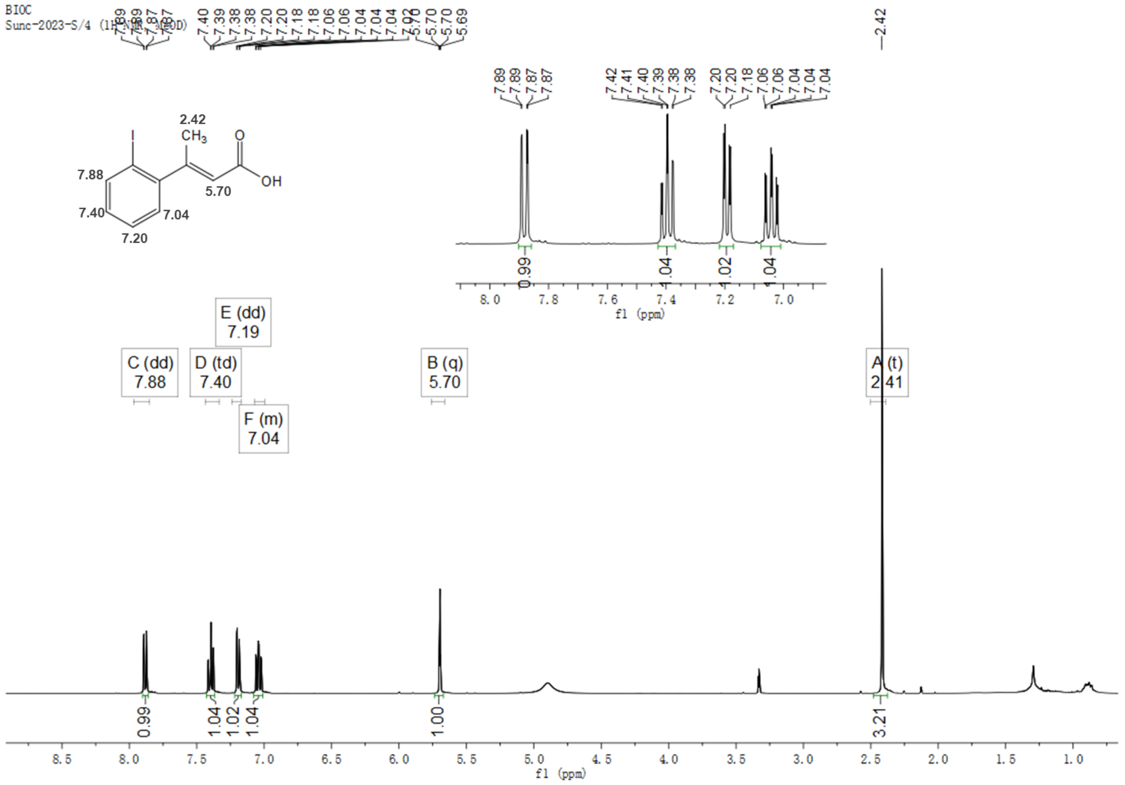

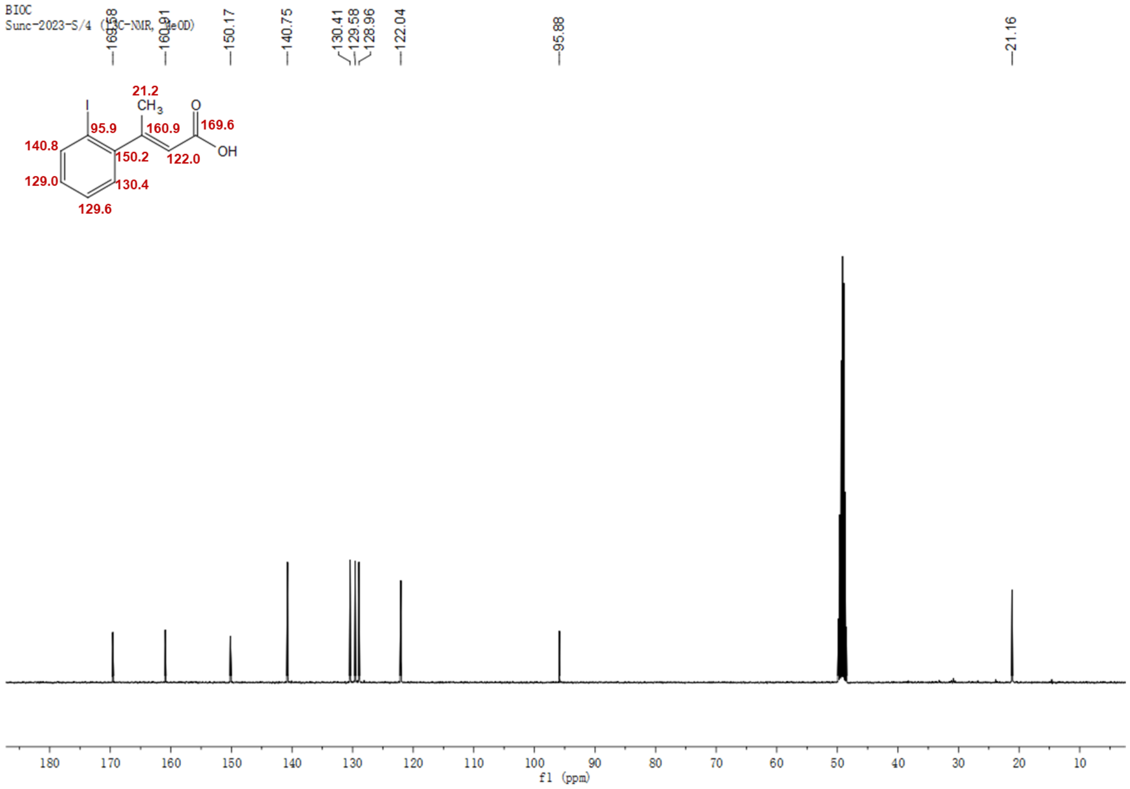

Figure S23. NMR spectra of **13**. ^{1}H-NMR (400 MHz, MeOD-d4); ^{13}C-NMR (101 MHz, MeOD-d4).

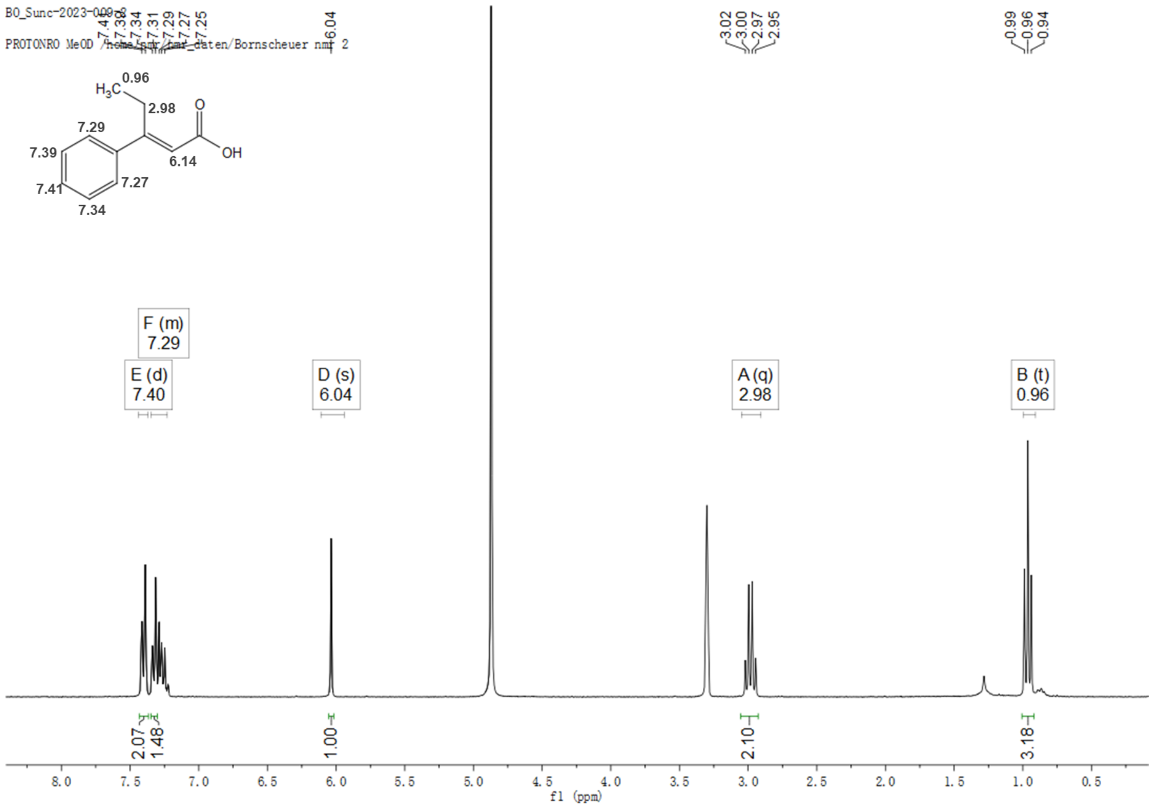

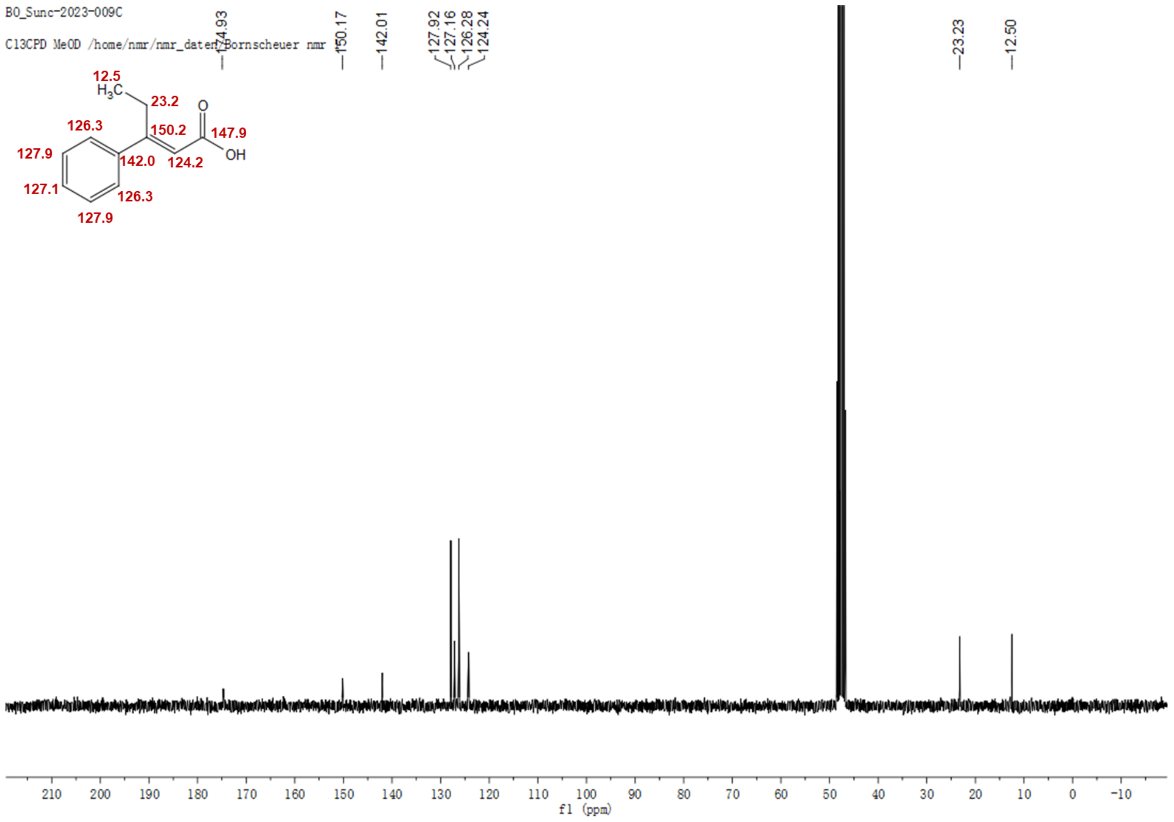

Figure S24. NMR spectra of **14**. ^{1}H-NMR (300 MHz, MeOD-d4); ^{13}C-NMR (75 MHz, MeOD-d4).

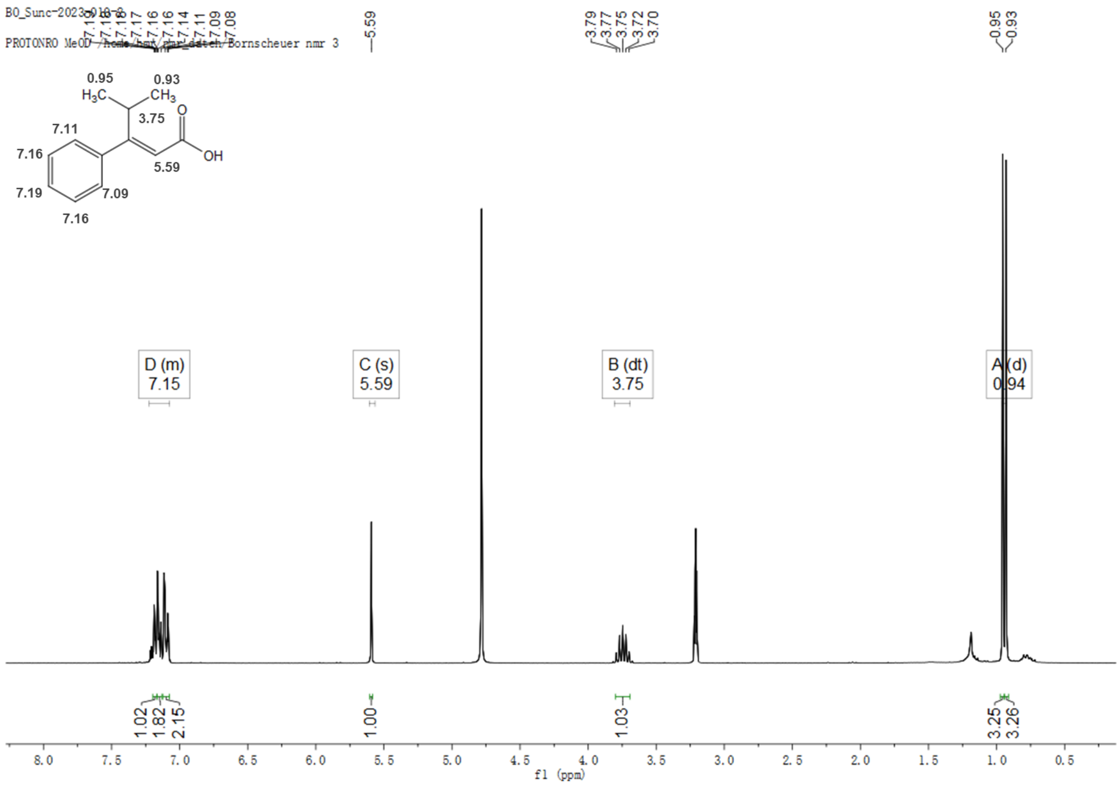

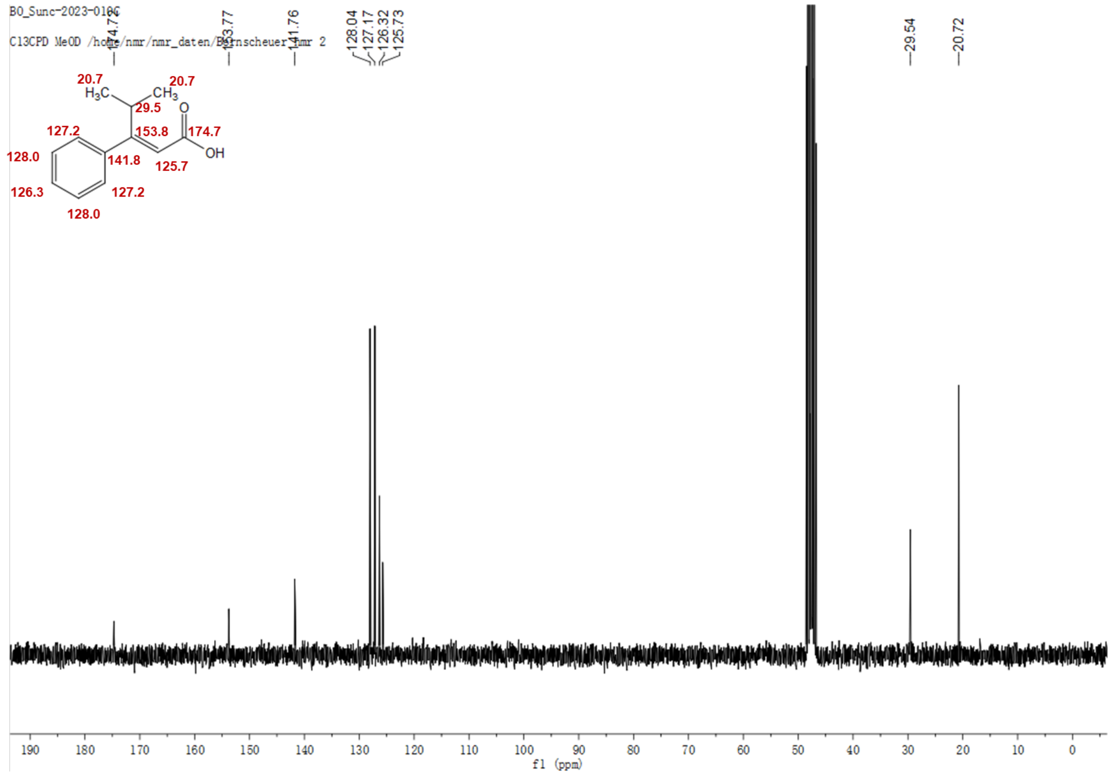

Figure S25. NMR spectra of **15**. ^{1}H-NMR (300 MHz, MeOD-d4); ^{13}C-NMR (75 MHz, MeOD-d4).

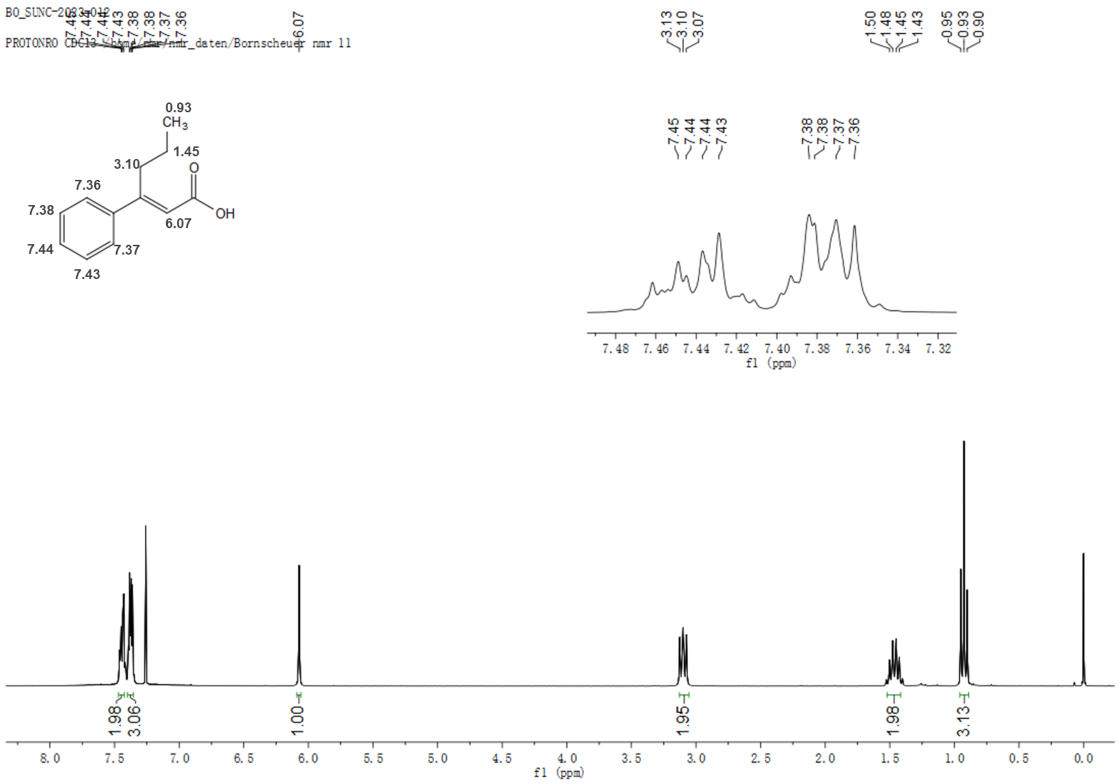

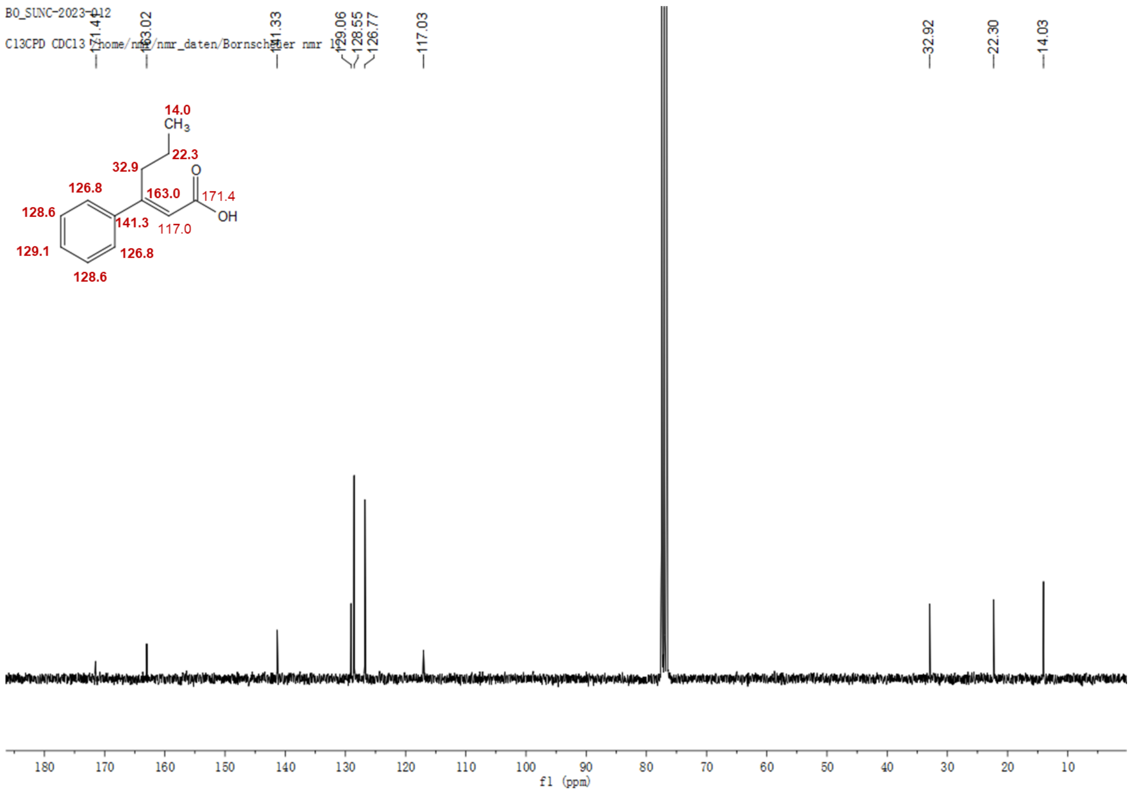

Figure S26. NMR spectra of **16**. ^{1}H-NMR (300 MHz, $CDCl_3$); ^{13}C-NMR (75 MHz, $CDCl_3$).

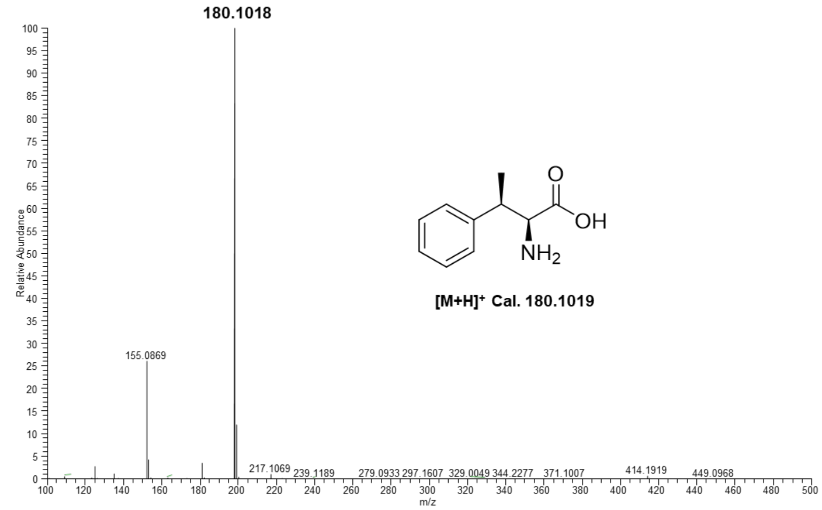
180.1018
155.0869
217.1069
239.1189
279.0933
297.1607
329.0049
344.2277
371.1007
414.1919
449.0968
Relative Abundance
m/z
OH
NH2
[M+H]+ Cal. 180.1019

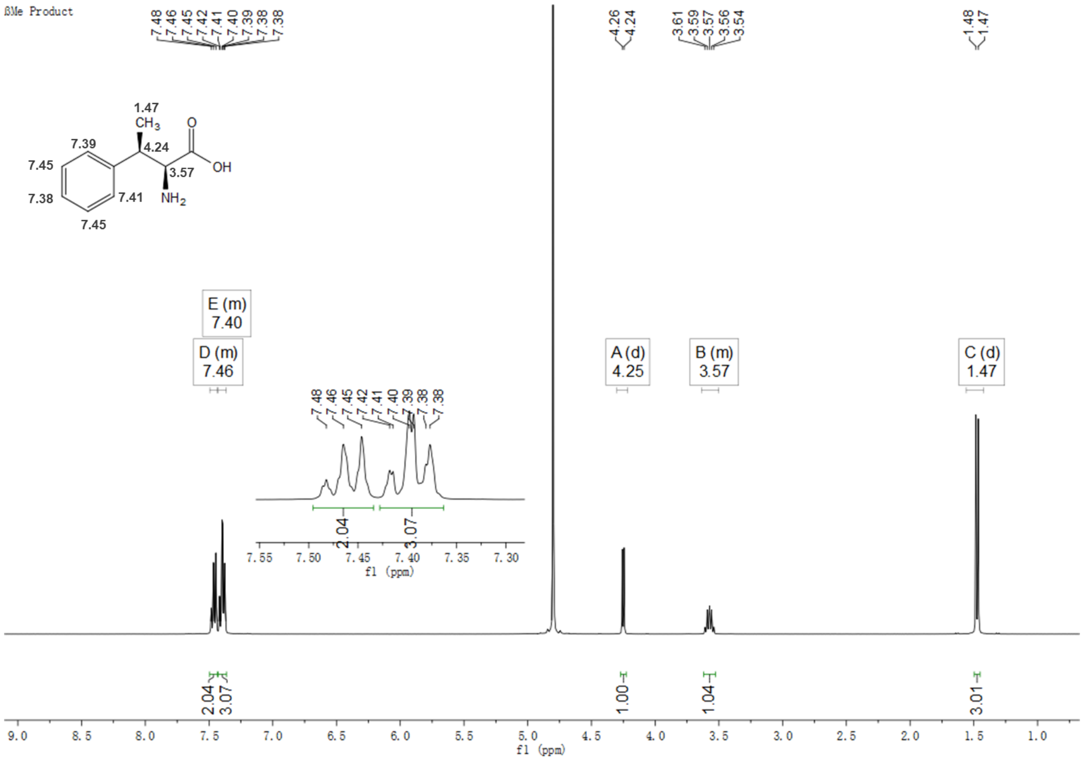
ßMe Product
7.48
7.46
7.45
7.42
7.41
7.40
7.39
7.38
7.38
4.26
4.24
3.61
3.59
3.57
3.56
3.54
1.48
1.47
E (m)
7.40
D (m)
7.46
A (d)
4.25
B (m)
3.57
C (d)
1.47
2.04
3.07
1.00
1.04
3.01
f1 (ppm)

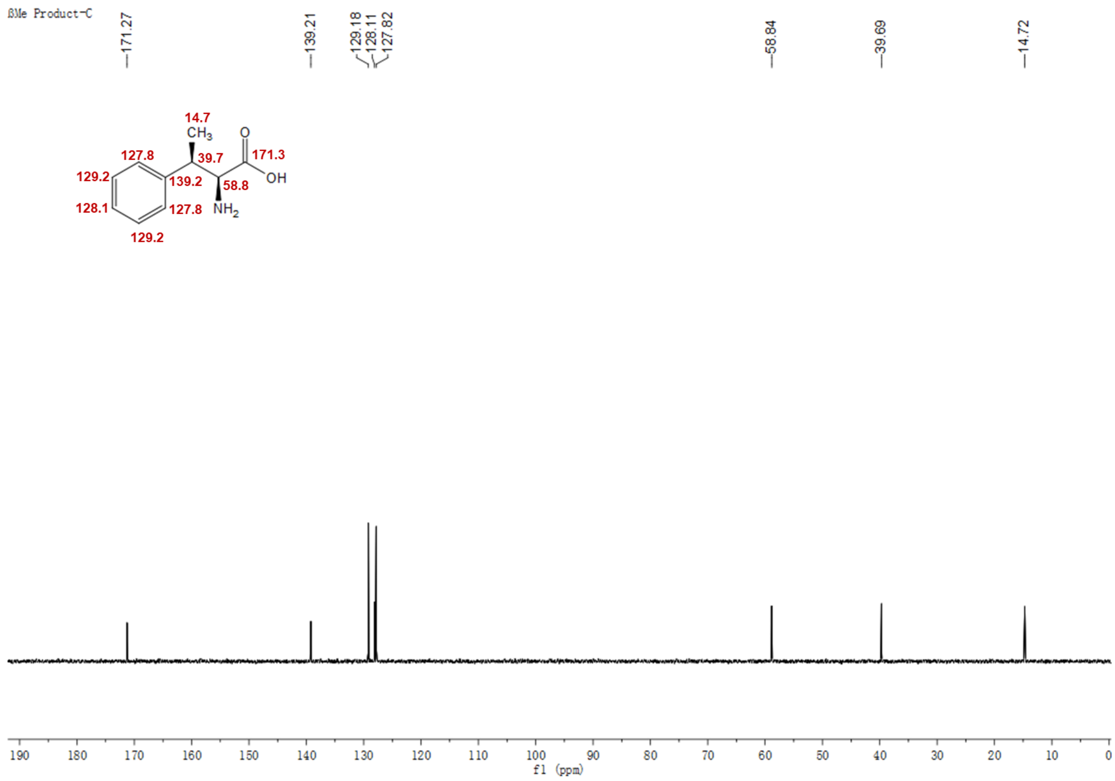

Figure S27. HRMS and NMR spectra of **1a**. ^{1}H-NMR (400 MHz, D_2O); ^{13}C-NMR (101 MHz, D_2O).

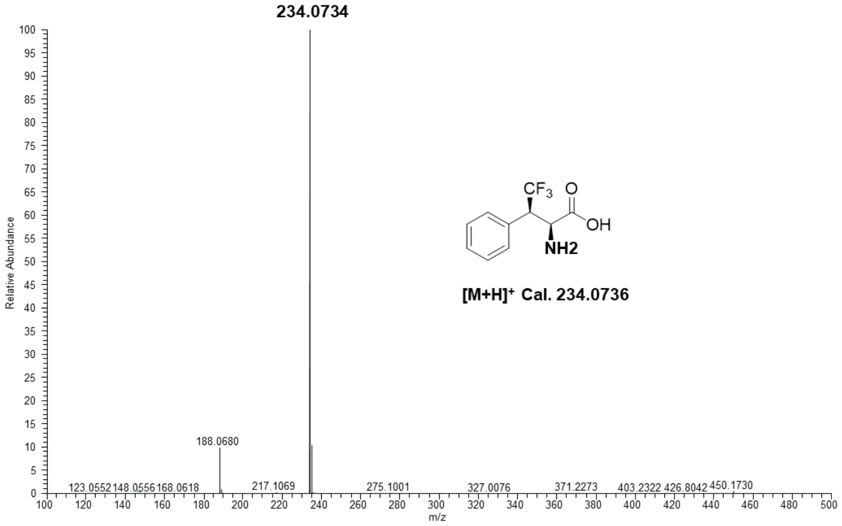
234.0734
Relative Abundance
188.0680
123.0552
148.0556
168.0618
217.1069
275.1001
327.0076
371.2273
403.2322
426.8042
450.1730
m/z
CF3
O
OH
NH2
[M+H]+ Cal. 234.0736

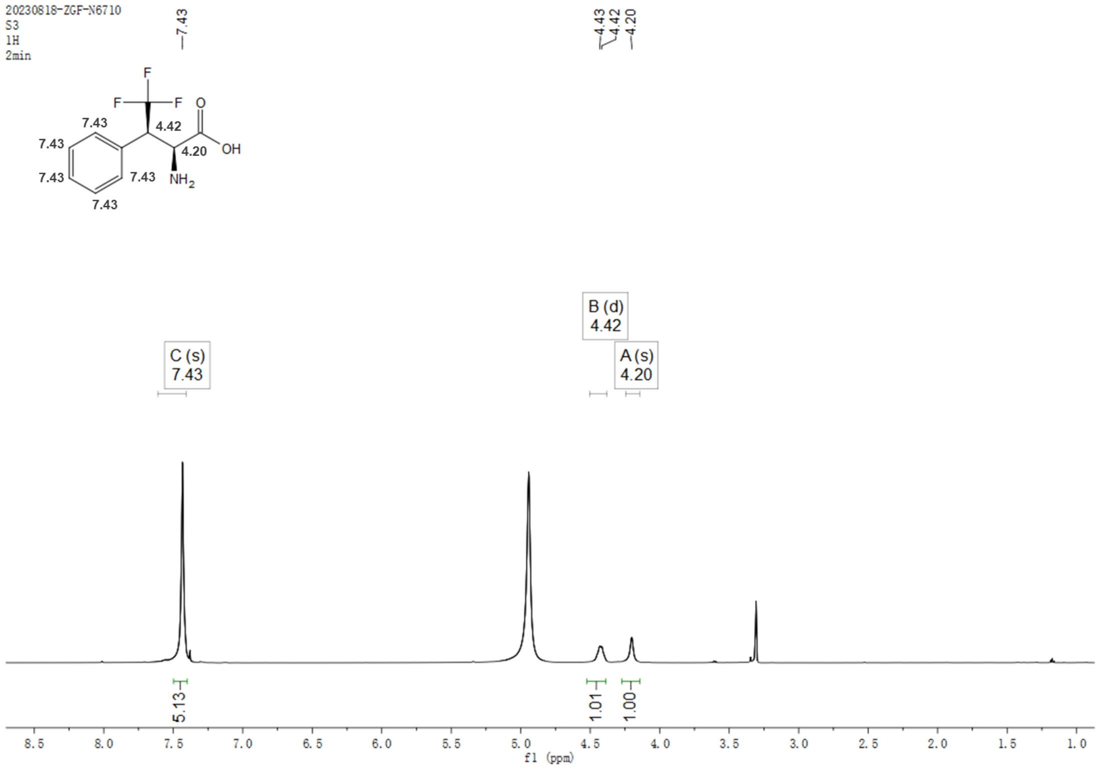
20230818-ZGF-N6710
S3
1H
2min
7.43
4.43
4.42
4.20
F
O
OH
NH2
B (d)
4.42
C (s)
7.43
A (s)
4.20
5.13
1.01
1.00
f1 (ppm)

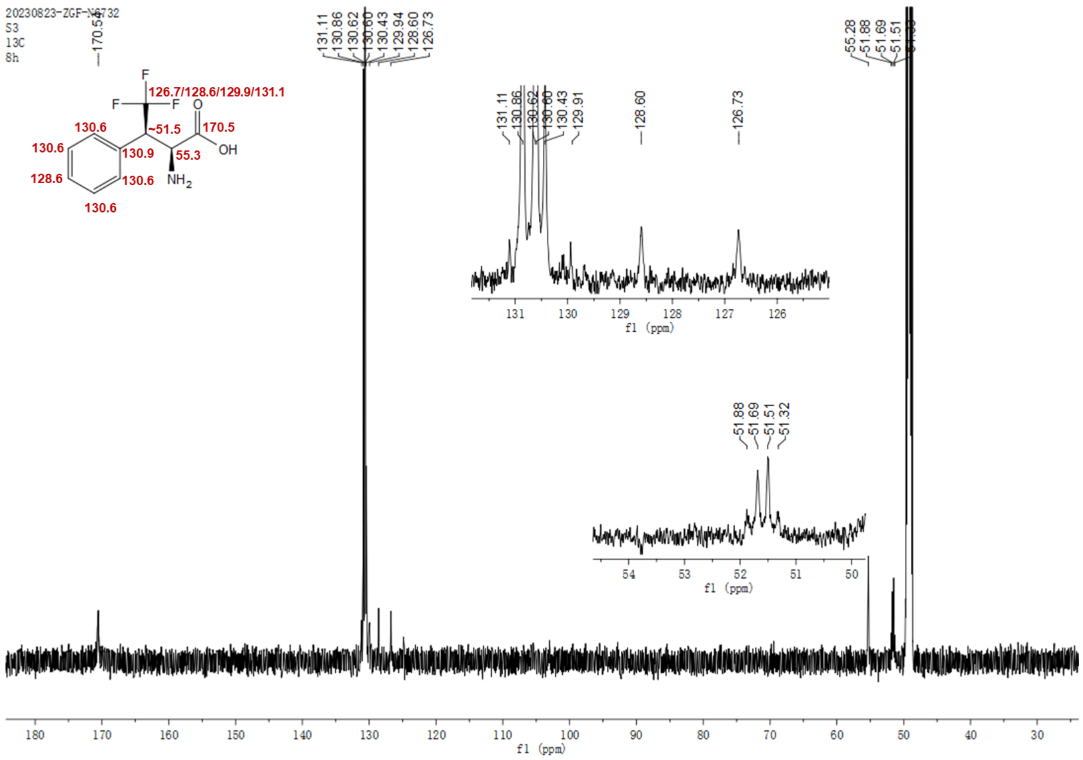

Figure S28. HRMS and NMR spectra of **2a**. ^{1}H-NMR (600 MHz, D_2O); ^{13}C-NMR (151 MHz, D_2O).

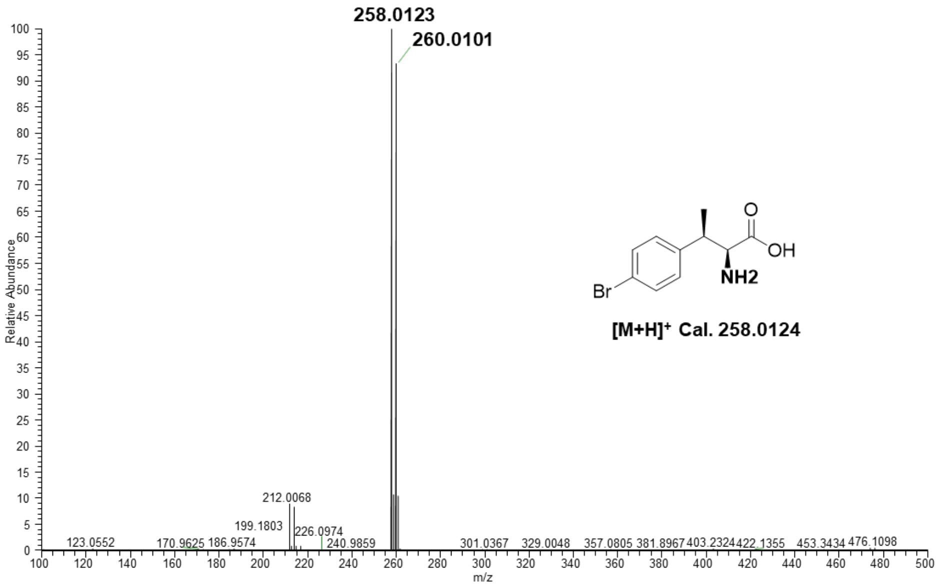
258.0123
260.0101
Relative Abundance
m/z
212.0068
199.1803
226.0974
240.9859
123.0552
170.9625
186.9574
301.0367
329.0048
357.0805
381.8967
403.2324
422.1355
453.3434
476.1098
Br
NH2
OH
O
[M+H]+ Cal. 258.0124

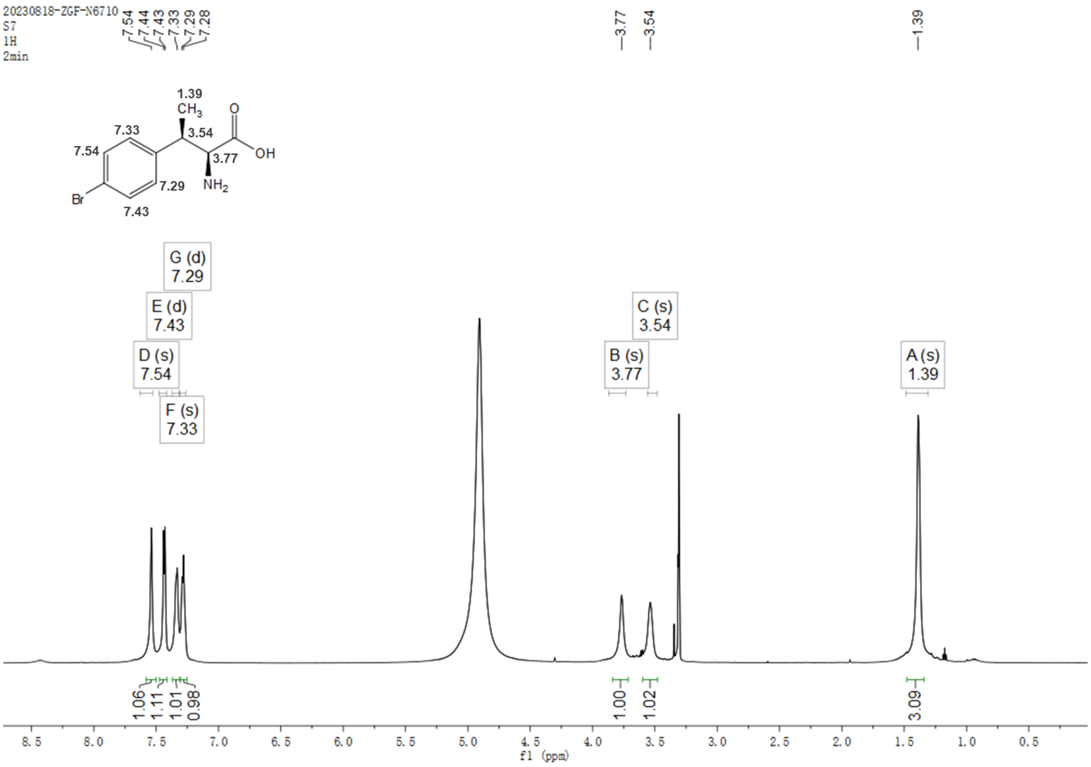
20230818-ZGF-N6710
S7
1H
2min
7.54
7.44
7.43
7.33
7.29
7.28
3.77
3.54
1.39
CH3
NH2
OH
Br
G (d) 7.29
E (d) 7.43
D (s) 7.54
F (s) 7.33
C (s) 3.54
B (s) 3.77
A (s) 1.39
1.06
1.11
1.01
0.98
1.00
1.02
3.09
f1 (ppm)

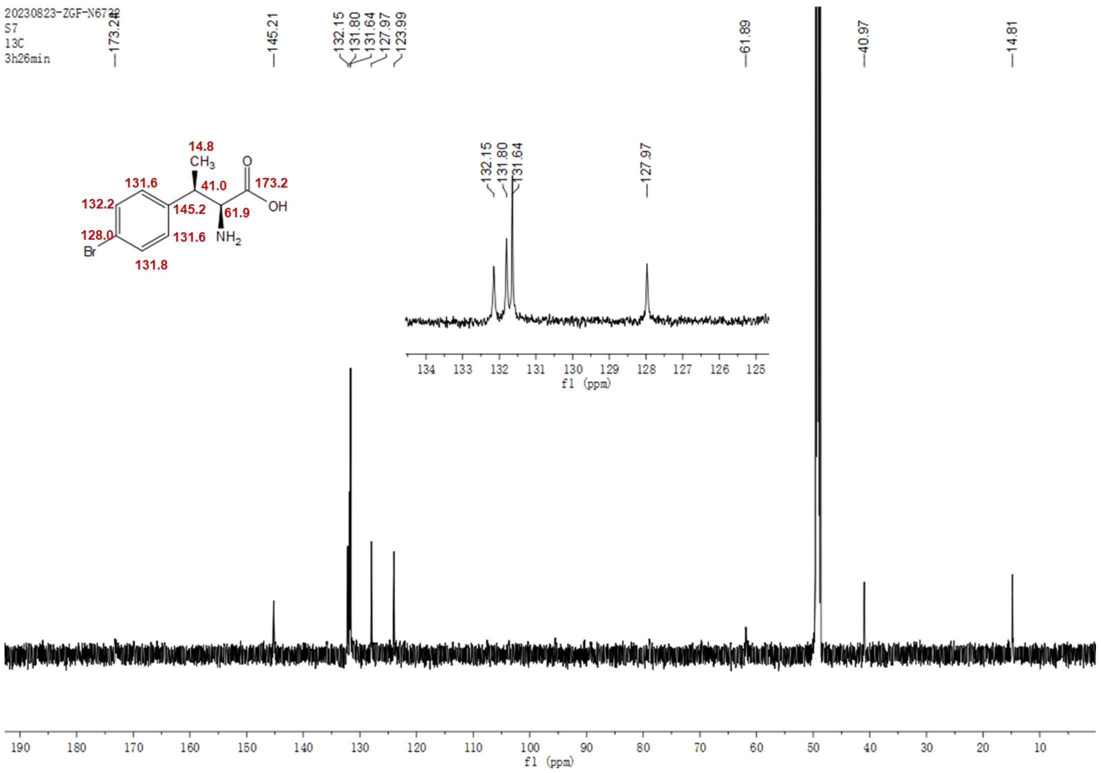

Figure S29. HRMS and NMR spectra of **3a**. ^{1}H-NMR (600 MHz, D_2O); ^{13}C-NMR (151 MHz, D_2O).

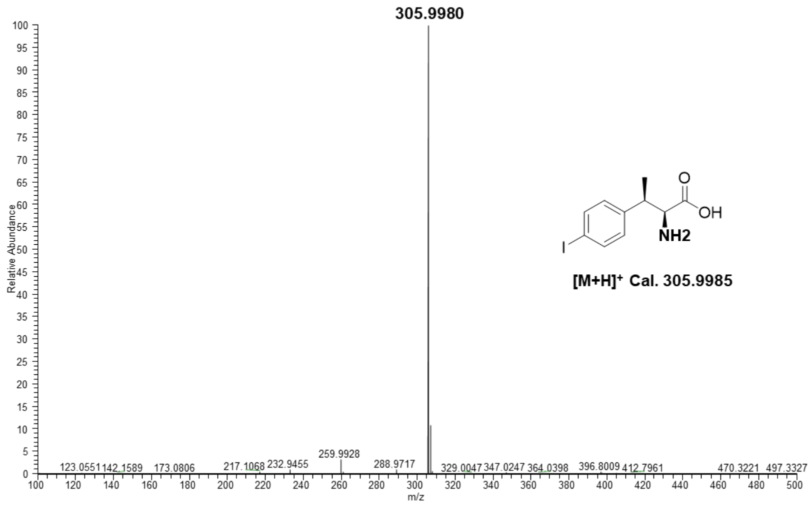
305.9980
Relative Abundance
m/z
259.9928
288.9717
123.0551
142.1589
173.0806
217.1068
232.9455
329.0047
347.0247
364.0398
396.8009
412.7961
470.3221
497.3327
OH
NH2
I
[M+H]+ Cal. 305.9985

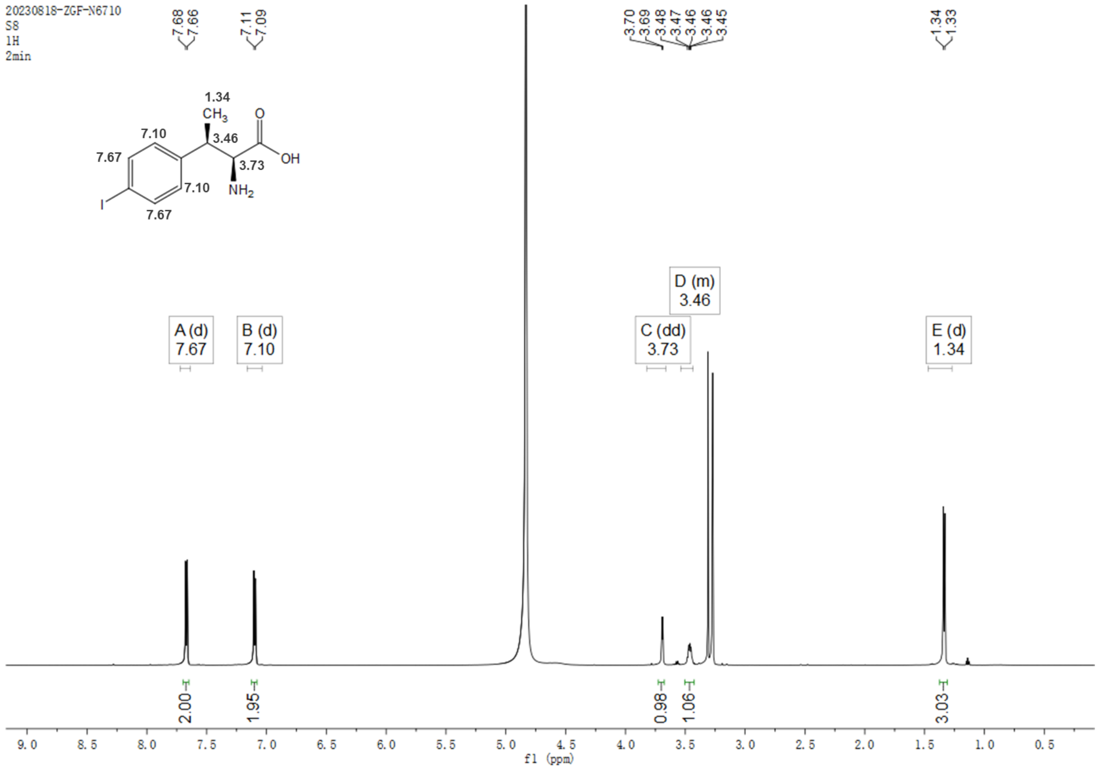
20230818-ZGF-N6710
S8
1H
2min
7.68
7.66
7.11
7.09
3.70
3.69
3.48
3.47
3.46
3.46
3.45
1.34
1.33
1.34
CH3
3.46
3.73
7.10
7.67
OH
NH2
I
A (d)
7.67
B (d)
7.10
C (dd)
3.73
D (m)
3.46
E (d)
1.34
2.00
1.95
0.98
1.06
3.03
f1 (ppm)

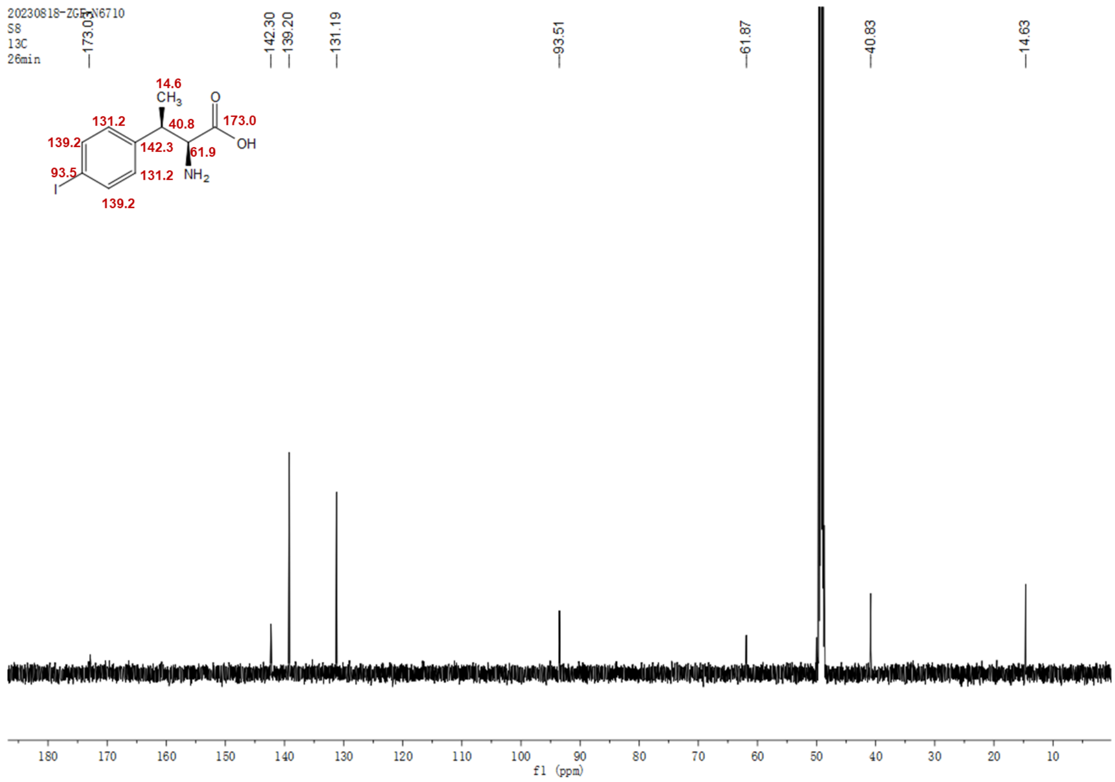

Figure S30. HRMS and NMR spectra of **4a**. ^{1}H-NMR (600 MHz, D_2O); ^{13}C-NMR (151 MHz, D_2O).

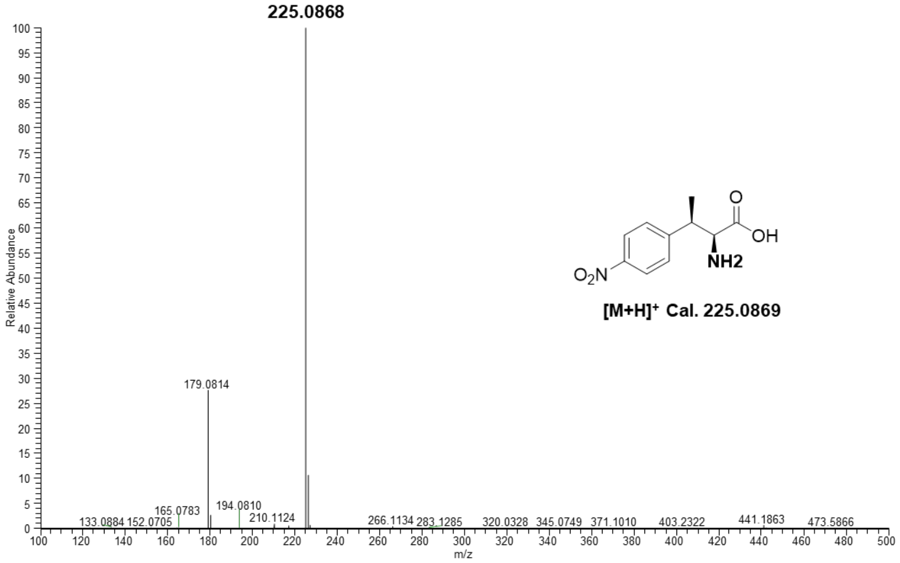
225.0868
179.0814
165.0783
194.0810
210.1124
133.0884 152.0705
266.1134 283.1285 320.0328 345.0749 371.1010 403.2322 441.1863 473.5866
Relative Abundance
m/z
O2N
NH2
OH
O
[M+H]+ Cal. 225.0869

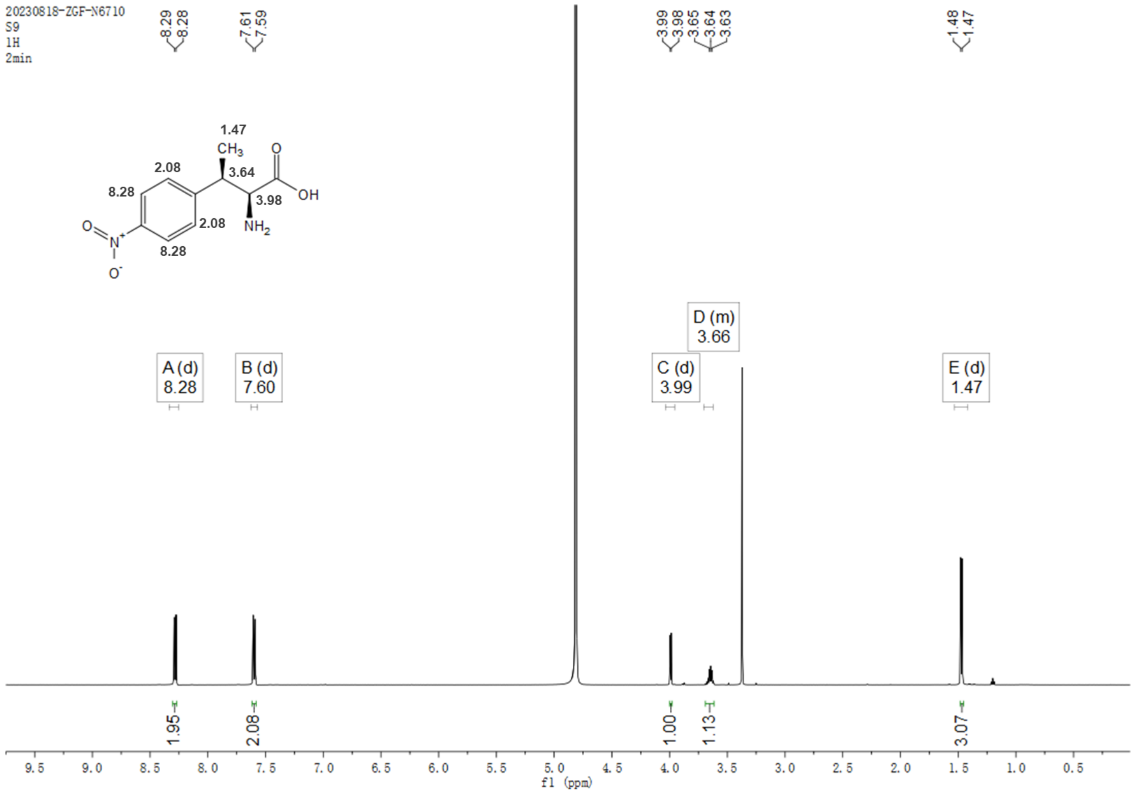
20230818-ZGF-N6710
S9
1H
2min
8.29
8.28
7.61
7.59
3.99
3.98
3.65
3.64
3.63
1.48
1.47
1.47
CH3
3.64
2.08
8.28
3.98
OH
2.08
NH2
8.28
A (d)
8.28
B (d)
7.60
C (d)
3.99
D (m)
3.66
E (d)
1.47
1.95
2.08
1.00
1.13
3.07
f1 (ppm)

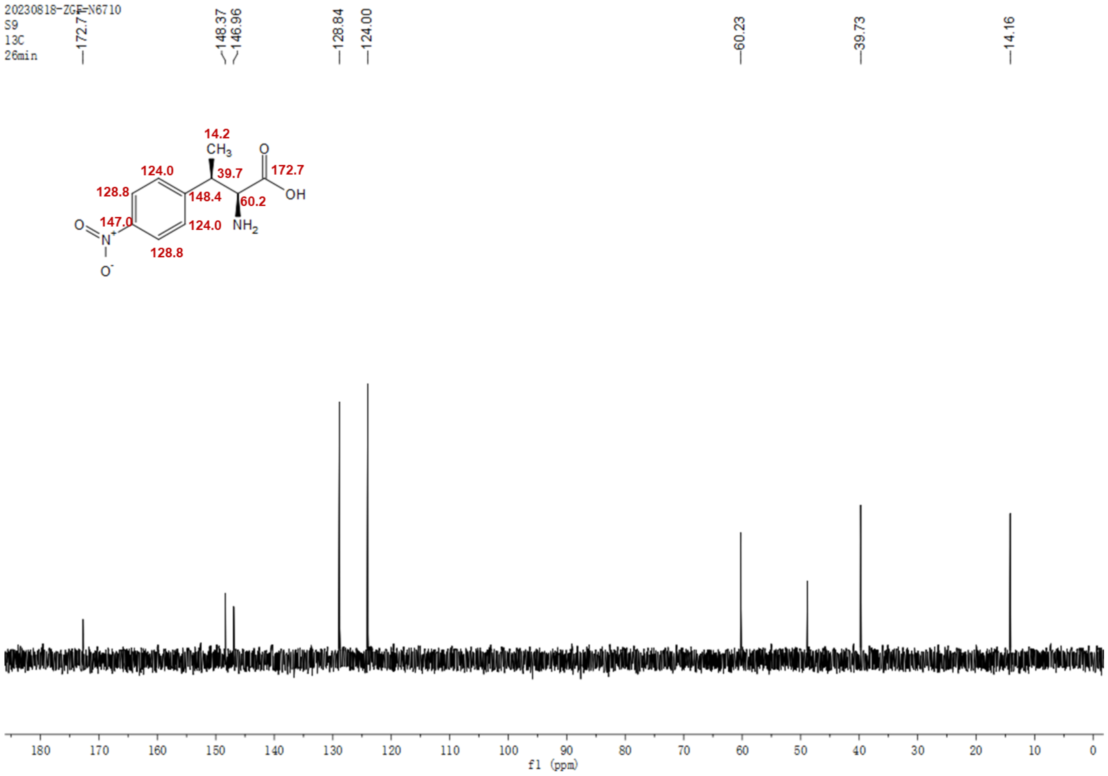

Figure S31. HRMS and NMR spectra of **5a**. ^{1}H-NMR (600 MHz, D_2O); ^{13}C-NMR (151 MHz, D_2O).

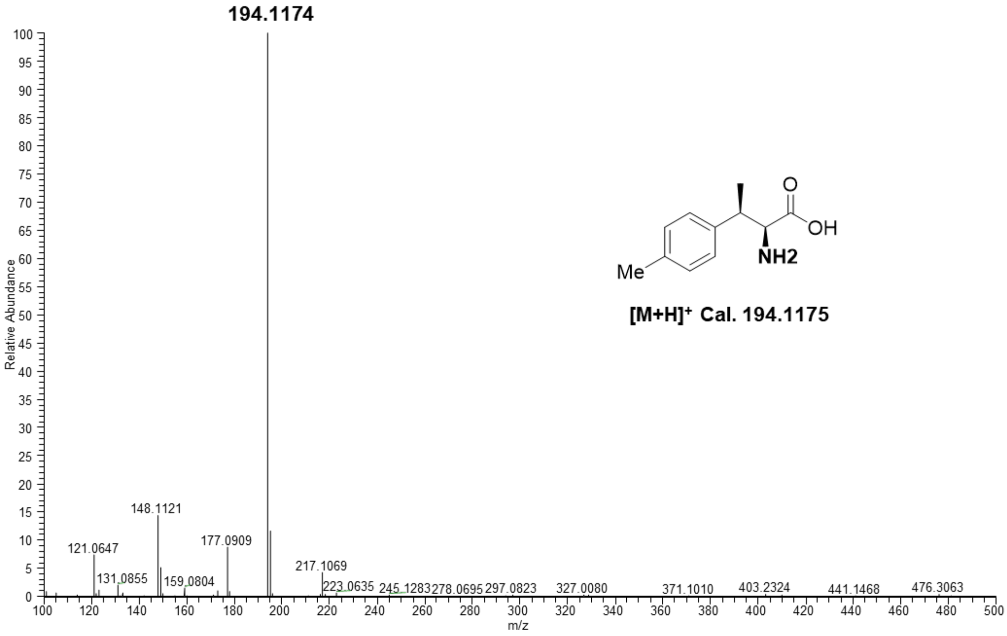
194.1174
Relative Abundance
m/z
148.1121
121.0647
177.0909
217.1069
131.0855
159.0804
223.0635
245.1283
278.0695
297.0823
327.0080
371.1010
403.2324
441.1468
476.3063
Me
OH
O
NH2
[M+H]+ Cal. 194.1175

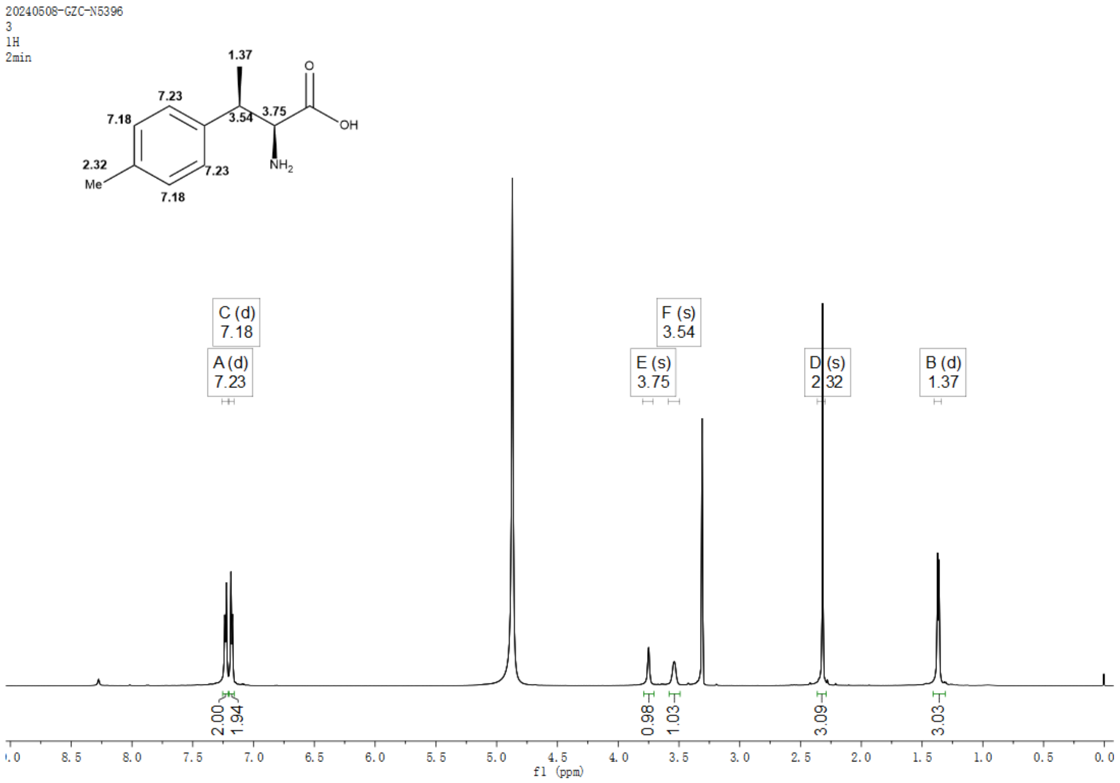
20240508-GZC-N5396
3
1H
2min
1.37
7.23
7.18
3.54
3.75
OH
2.32
Me
7.23
7.18
NH2
C (d)
7.18
A (d)
7.23
F (s)
3.54
E (s)
3.75
D (s)
2.32
B (d)
1.37
2.00
1.94
0.98
1.03
3.09
3.03
f1 (ppm)

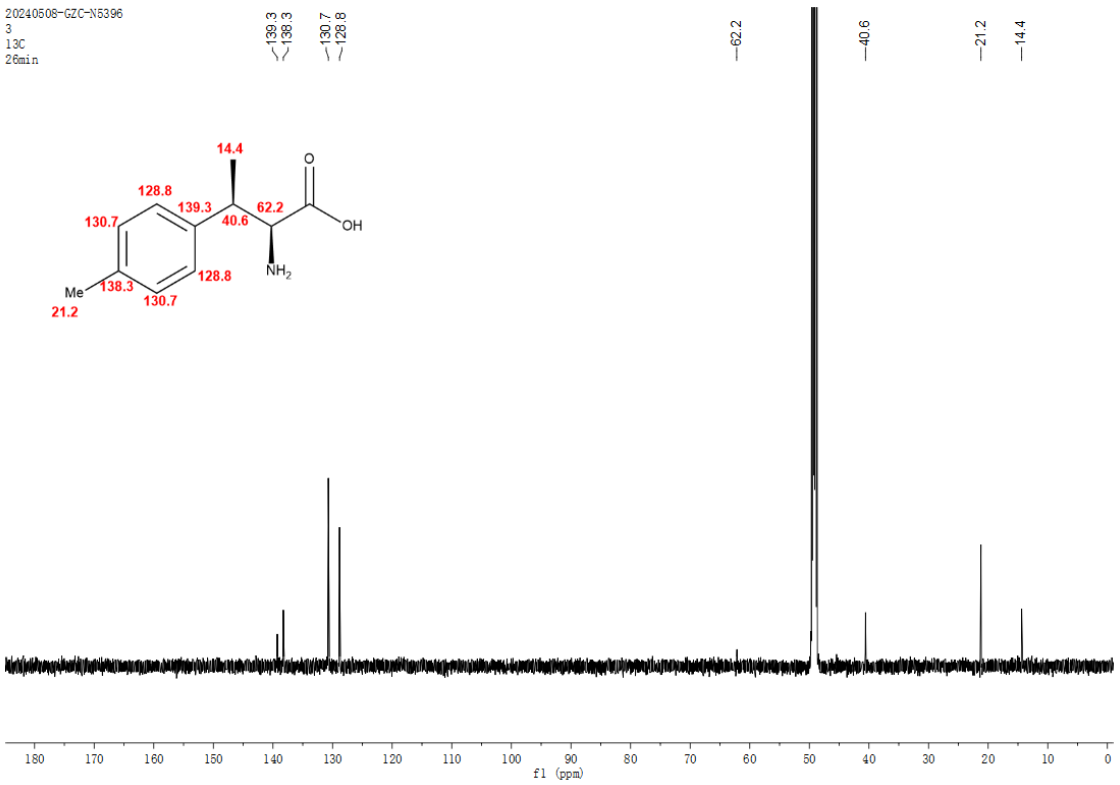

Figure S32. HRMS and NMR spectra of **6a**. ^{1}H-NMR (600 MHz, D_2O); ^{13}C-NMR (151 MHz, D_2O).

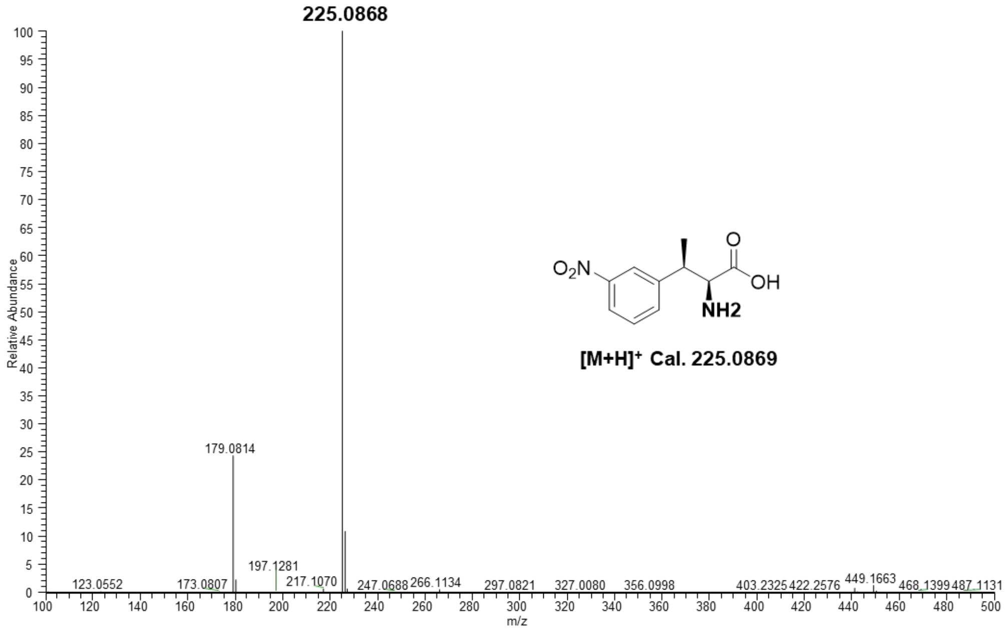
225.0868
Relative Abundance
179.0814
197.1281
217.1070
123.0552
173.0807
247.0688
266.1134
297.0821
327.0080
356.0998
403.2325
422.2576
449.1663
468.1399
487.1131
m/z
O2N
O
OH
NH2
[M+H]+ Cal. 225.0869

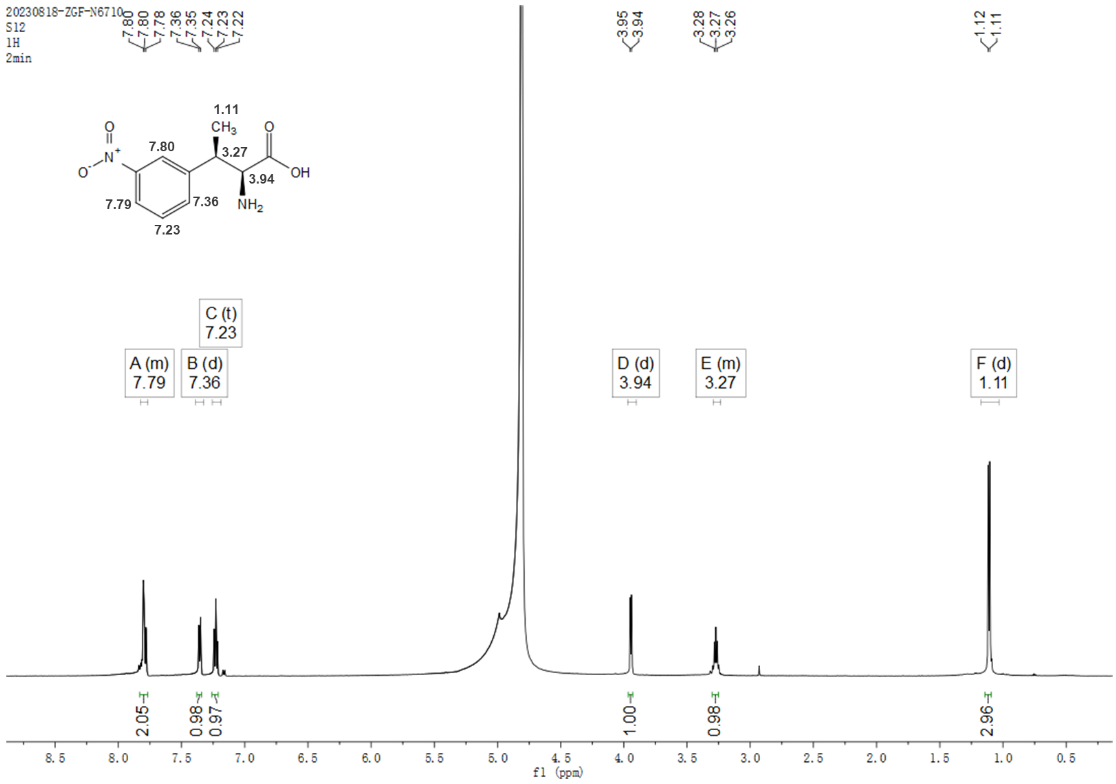
20230818-ZGF-N6710
S12
1H
2min
1.11
CH3
3.27
3.94
7.80
7.79
7.36
7.23
NH2
OH
A (m) 7.79
B (d) 7.36
C (t) 7.23
D (d) 3.94
E (m) 3.27
F (d) 1.11
2.05
0.98
0.97
1.00
0.98
2.96
f1 (ppm)

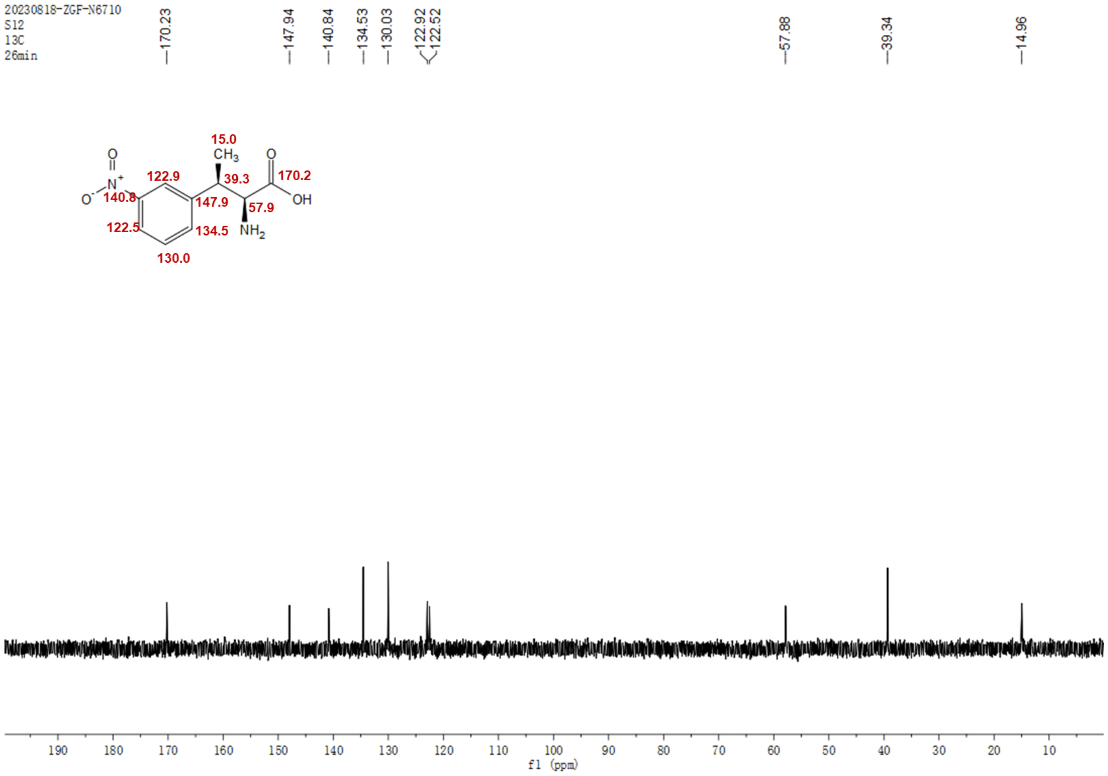

Figure S33. HRMS and NMR spectra of **7a**. ^{1}H-NMR (600 MHz, D_2O); ^{13}C-NMR (151 MHz, D_2O).

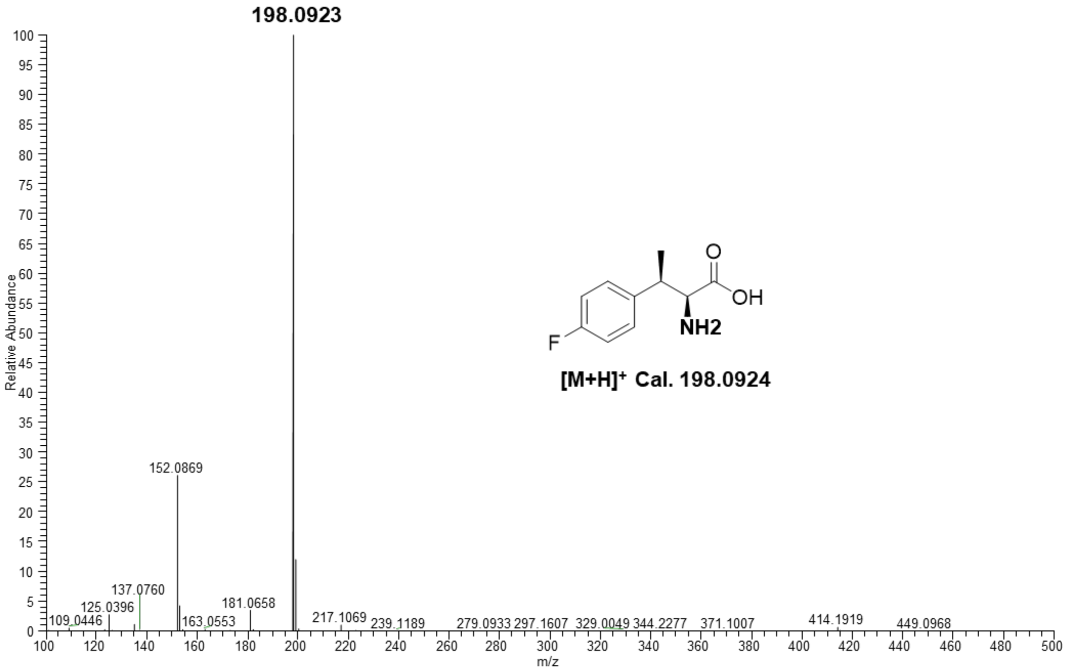
198.0923
Relative Abundance
152.0869
137.0760
125.0396
109.0446
163.0553
181.0658
217.1069
239.1189
279.0933
297.1607
329.0049
344.2277
371.1007
414.1919
449.0968
m/z
F
NH2
OH
O
[M+H]+ Cal. 198.0924

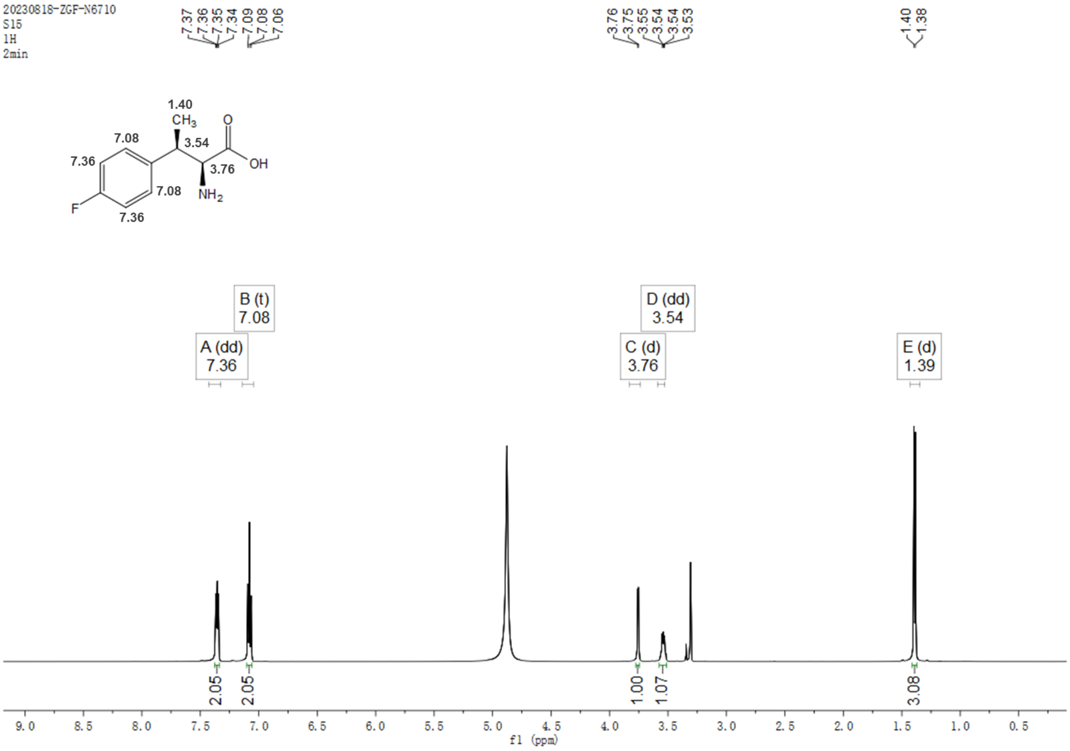
20230818-ZGF-N6710
S15
1H
2min
7.37
7.36
7.35
7.34
7.09
7.08
7.06
3.76
3.75
3.55
3.54
3.54
3.53
1.40
1.38
1.40
CH3
3.54
3.76
7.08
7.36
7.08
7.36
F
NH2
OH
O
A (dd)
7.36
B (t)
7.08
C (d)
3.76
D (dd)
3.54
E (d)
1.39
2.05
2.05
1.00
1.07
3.08
f1 (ppm)

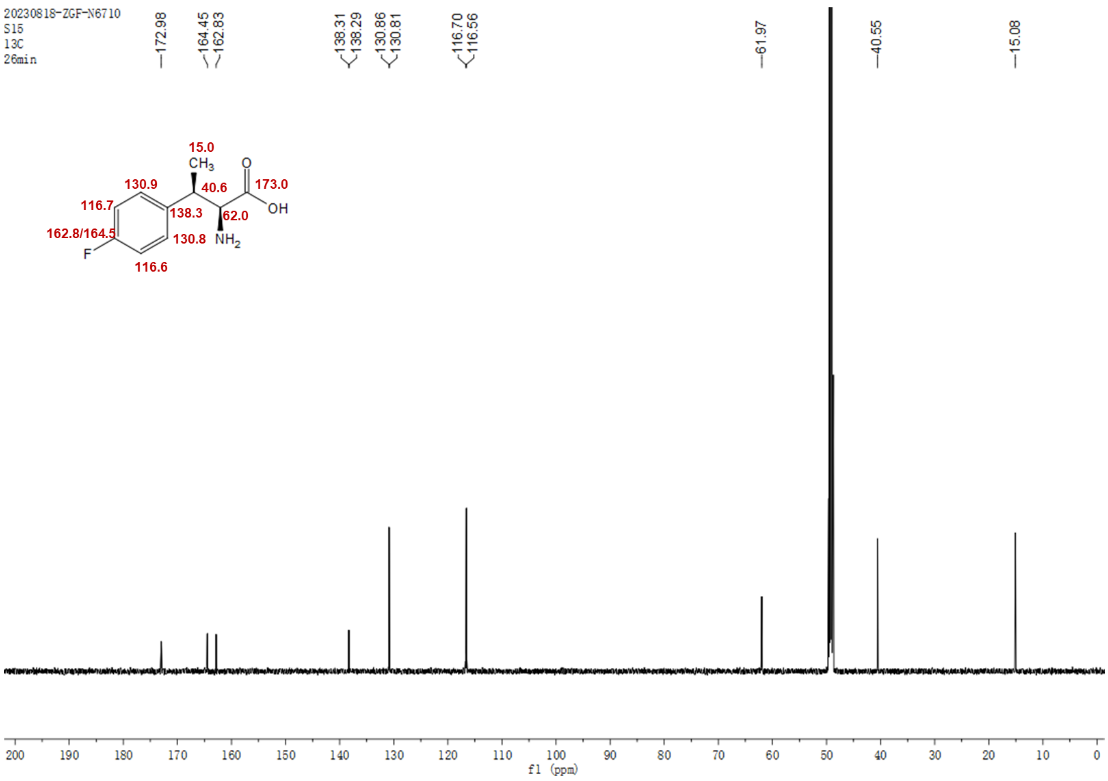

Figure S34. HRMS and NMR spectra of **8a**. ^{1}H-NMR (600 MHz, D_2O); ^{13}C-NMR (151 MHz, D_2O).

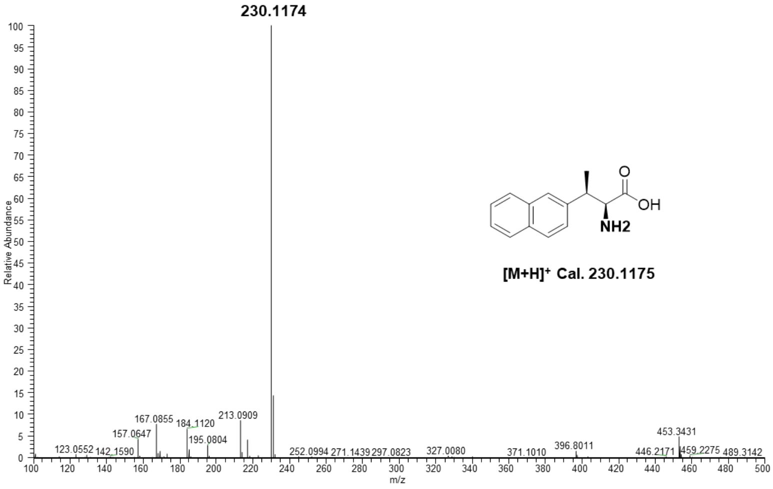
230.1174
[M+H]+ Cal. 230.1175
NH2
OH
O
Relative Abundance
m/z
167.0855
157.0647
184.1120
195.0804
213.0909
123.0552
142.1590
252.0994
271.1439
297.0823
327.0080
371.1010
396.8011
453.3431
446.2171
459.2275
489.3142

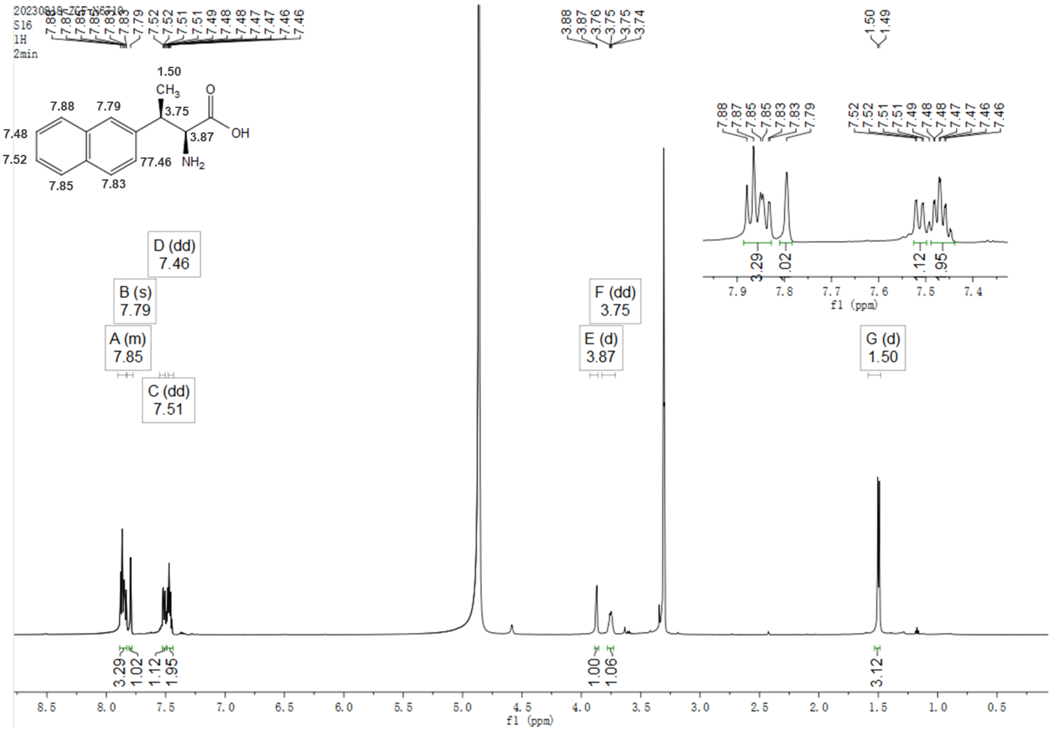
S16
1H
2min
1.50
CH3
3.75
3.87
7.88
7.79
7.48
7.52
7.85
7.83
77.46
NH2
OH
A (m)
7.85
B (s)
7.79
C (dd)
7.51
D (dd)
7.46
E (d)
3.87
F (dd)
3.75
G (d)
1.50
3.29
1.02
1.12
1.95
1.00
1.06
3.12
f1 (ppm)

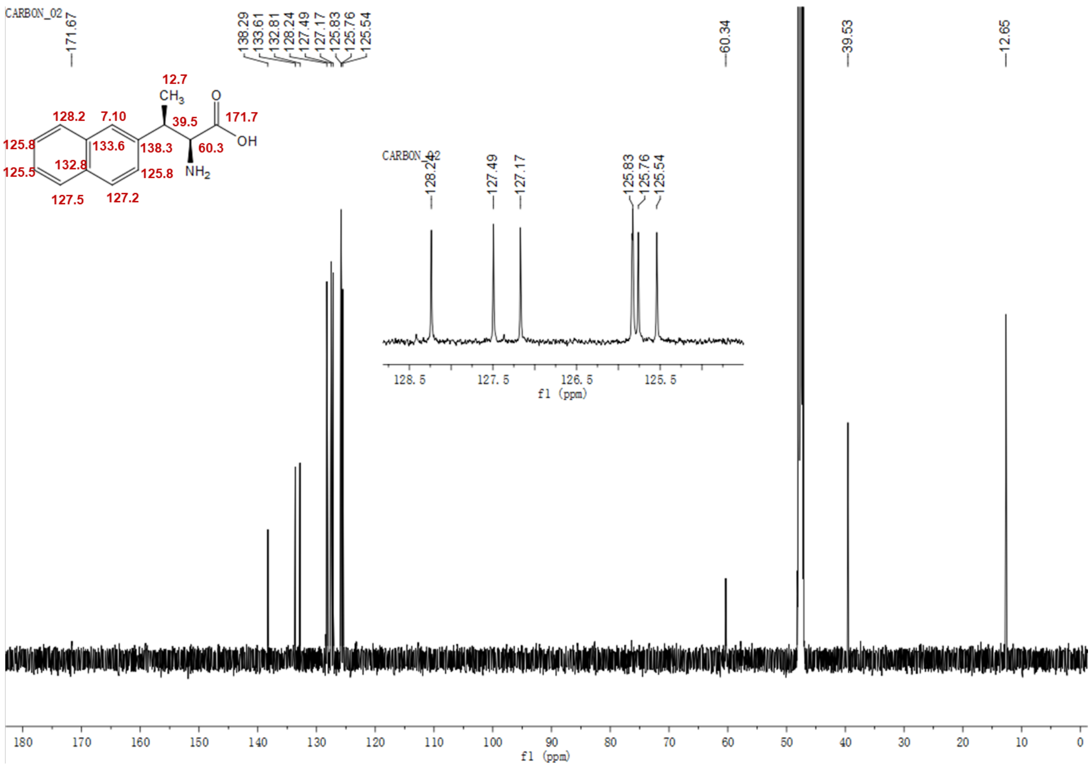

Figure S35. HRMS and NMR spectra of **9a**. ^{1}H-NMR (600 MHz, D_2O); ^{13}C-NMR (151 MHz, D_2O).

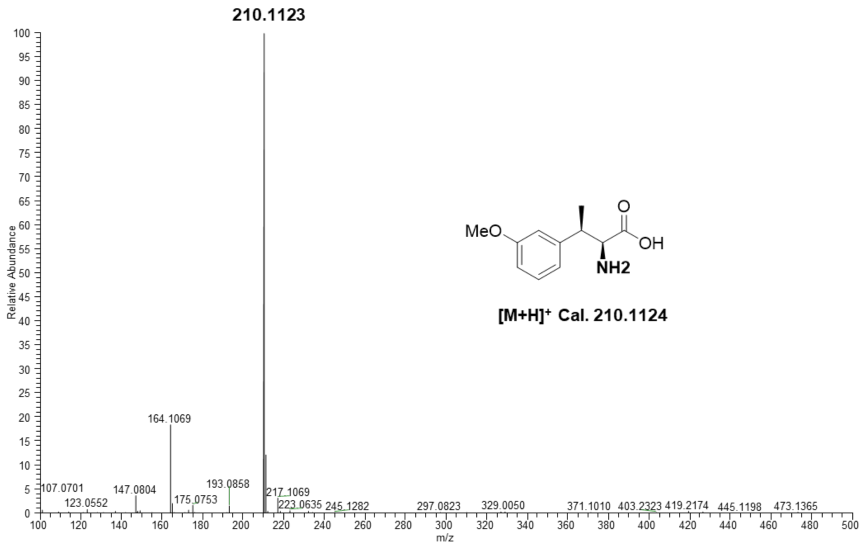
210.1123
Relative Abundance
m/z
164.1069
107.0701
123.0552
147.0804
175.0753
193.0858
217.1069
223.0635
245.1282
297.0823
329.0050
371.1010
403.2323
419.2174
445.1198
473.1365
MeO
O
OH
NH2
[M+H]+ Cal. 210.1124

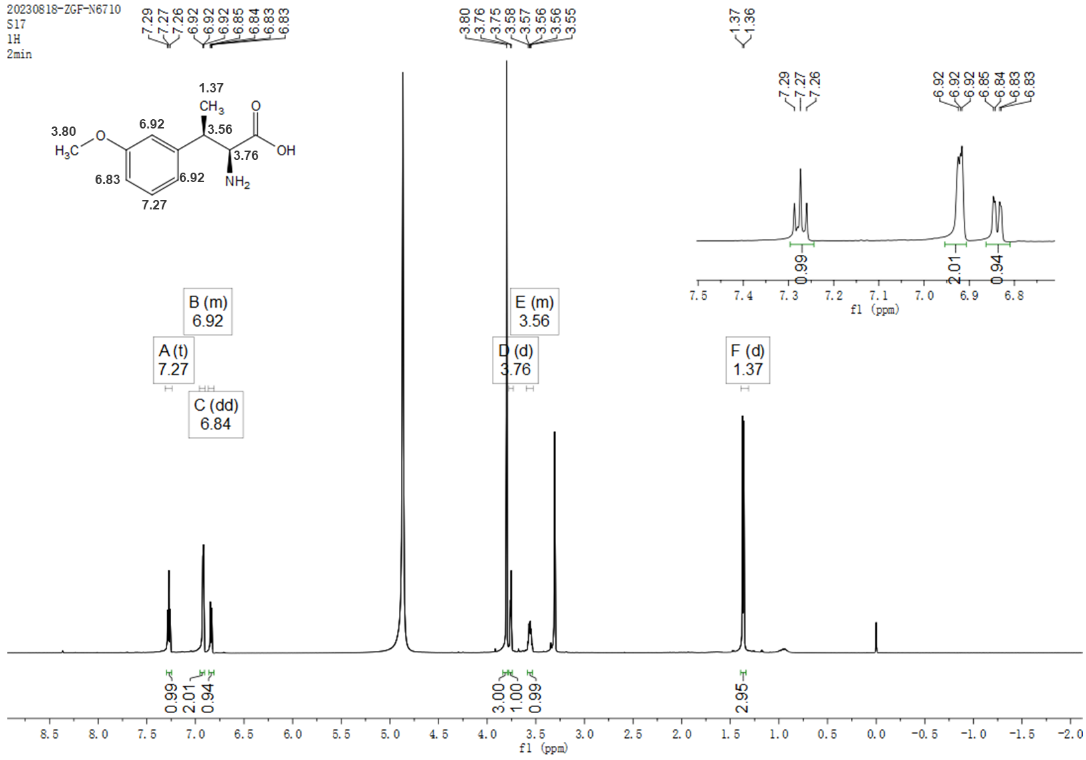
20230818-ZGF-N6710
S17
1H
2min
7.29
7.27
7.26
6.92
6.92
6.92
6.85
6.84
6.83
6.83
3.80
3.76
3.75
3.58
3.57
3.56
3.56
3.55
1.37
1.36
1.37
CH3
3.56
3.80
H3C
O
6.92
3.76
OH
NH2
6.83
6.92
7.27
B (m)
6.92
A (t)
7.27
C (dd)
6.84
E (m)
3.56
D (d)
3.76
F (d)
1.37
0.99
2.01
0.94
3.00
1.00
0.99
2.95
f1 (ppm)

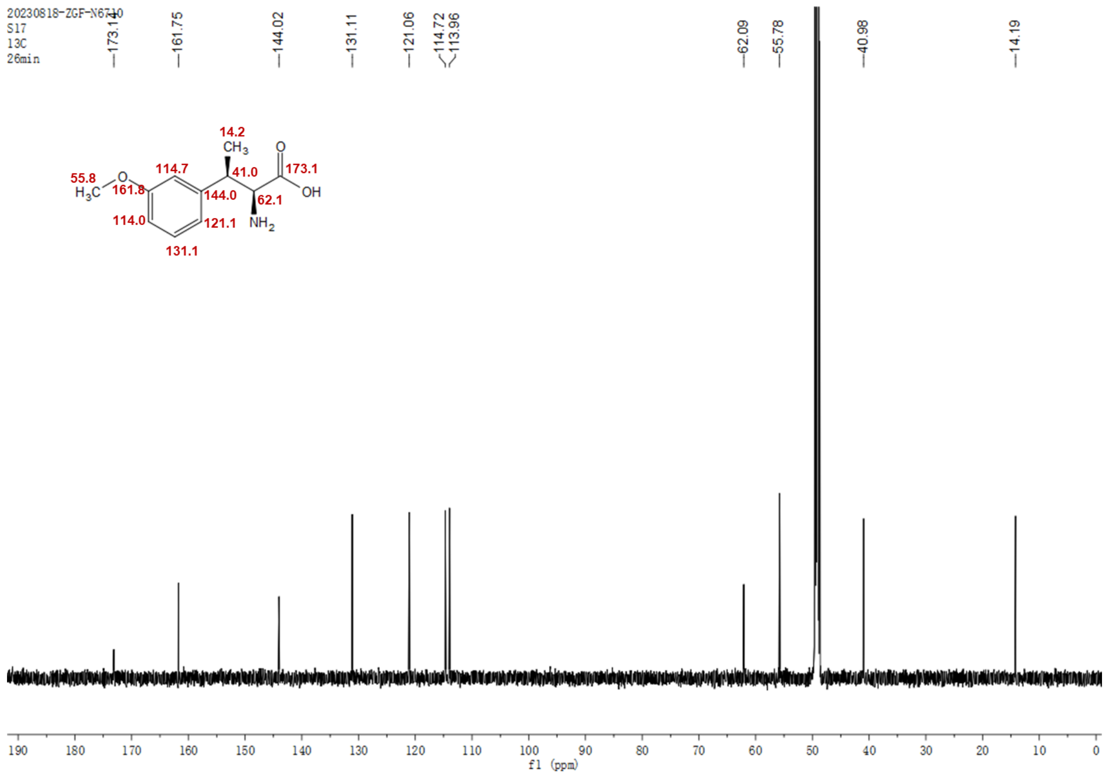

Figure S36. HRMS and NMR spectra of **10a**. ¹H-NMR (600 MHz, D_2O); ¹³C-NMR (151 MHz, D_2O).

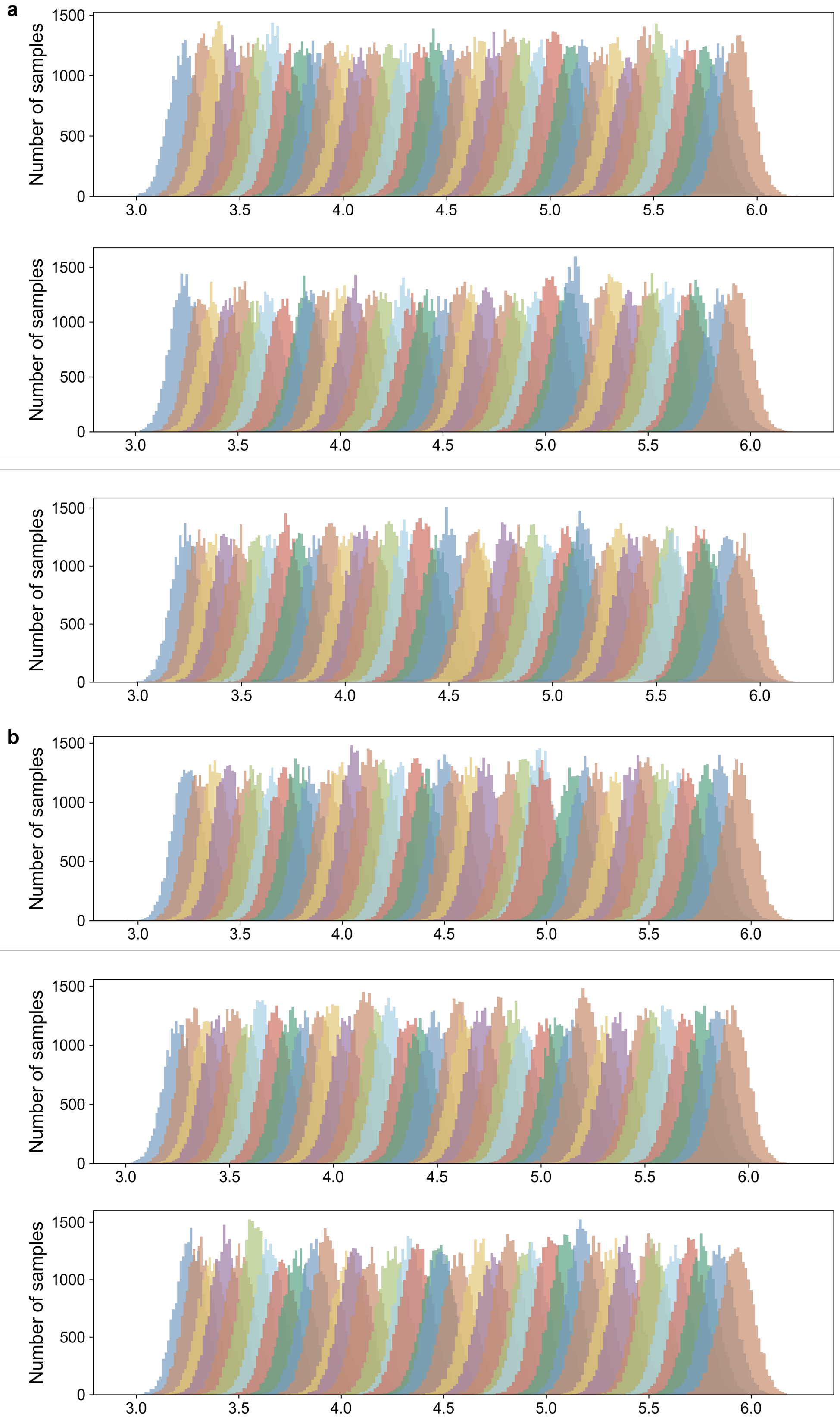
a
b
Number of samples
0
500
1000
1500
3.0
3.5
4.0
4.5
5.0
5.5
6.0

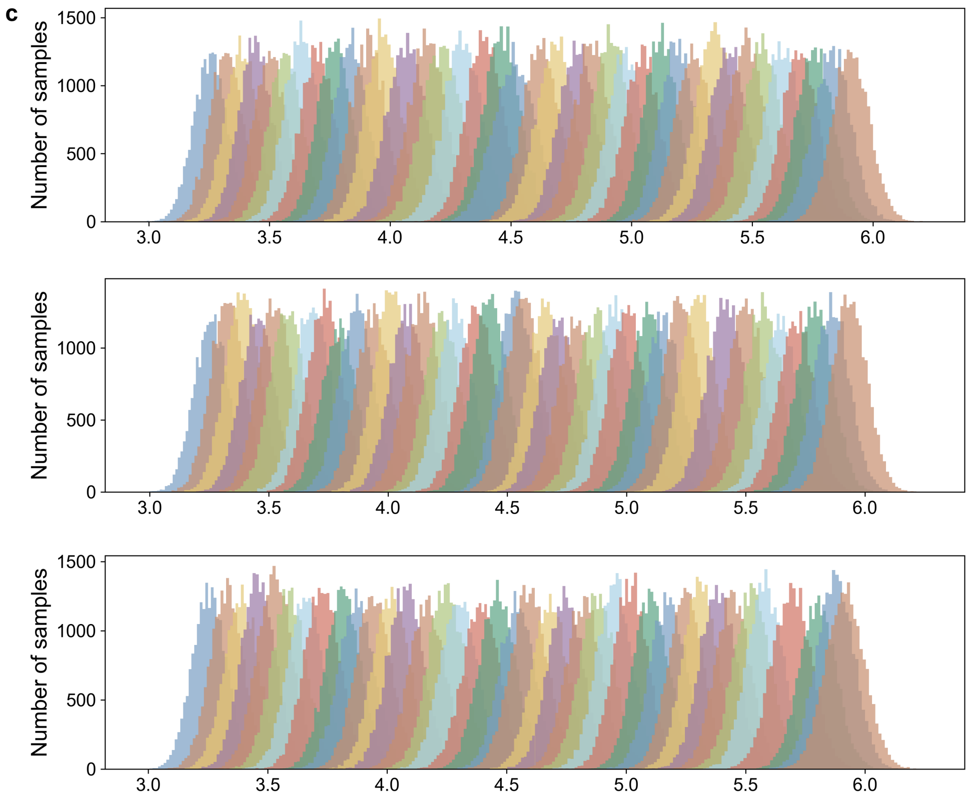

Figure S37. Overlap check of specific windows with histograms. The X-axis represents the reaction coordinate (distance from the N atom of MIO to αC of CA or βMeCA), while the Y-axis shows the corresponding frequency. Through this figure, one can visually observe the overlap of different windows on the reaction coordinate and the distribution of data within each window. **a**, for the (CA)/(*Pc*PAL-WT) complex. **b**, for the (β-MeCA)/(*Pc*PAL-WT) complex. **c**, for the (β-MeCA)/(*Pc*PAL-L256V-I460V) mutant complex.This figure presents a comparison of overlap checks and histograms for specific windows.

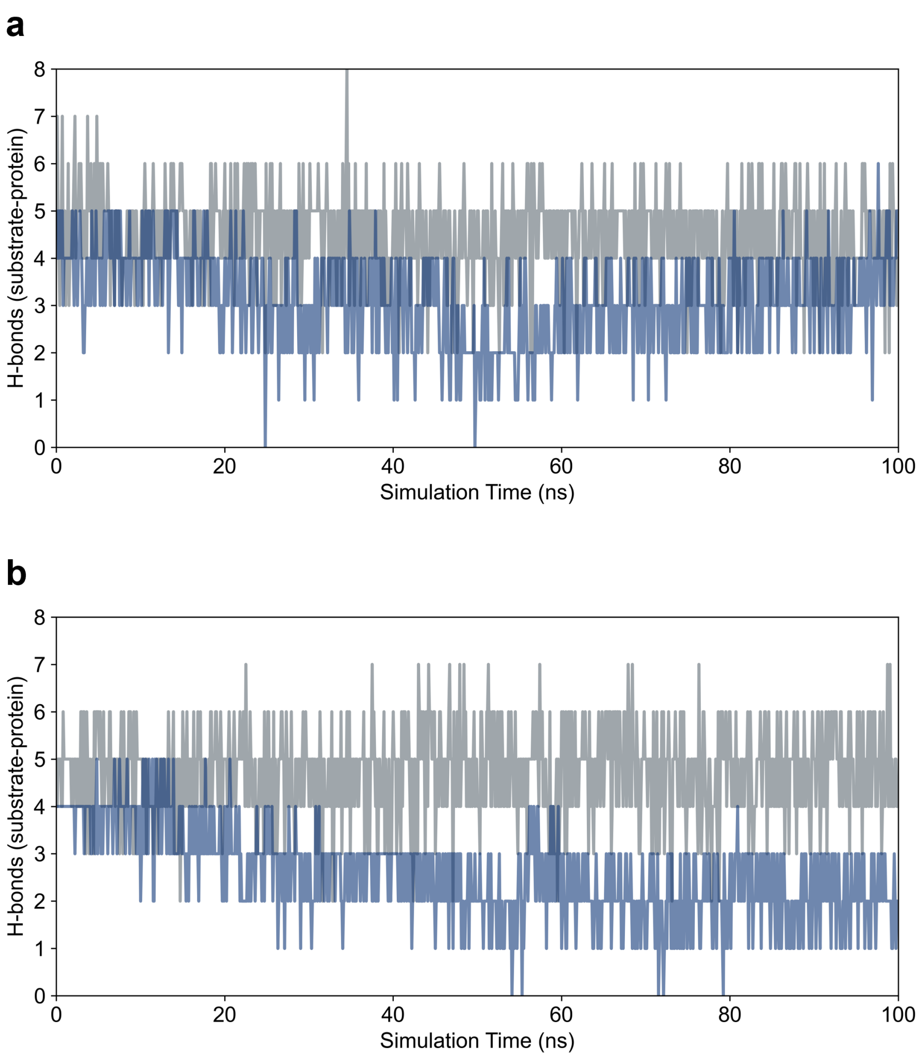

Figure S38. Molecular dynamics analysis of hydrogen bonds in protein-substrate complexes. The two colors represent two different parallel simulations. **a**, for the (CA)/(*Pc*PAL-WT) complex. **b**, for the (β-MeCA)/(*Pc*PAL-WT) complex.

3. Supplementary References

[1] J. Sambrook, D. W. Russell, Molecular cloning: a laboratory manual, 3rd ed., Cold Spring Harbor Laboratory Press, New York. (2001).

[2] Xiao, G., Xie, C., Guo, Q., Zi, G., Hou, G., & Huang, Y. Highly enantioselective Ni-catalyzed asymmetric hydrogenation of β, β-disubstituted acrylic acids. *Org. Chem. Front.*, 9(16), 4472-4477. (2022).

[3] Wu, S., Xiang, C., Zhou, Y., Khan, M. S. H., Liu, W., Feiler, C. G., Wei, R., Weber, G., Höhne, M. & Bornscheuer, U. T. A growth selection system for the directed evolution of amine-forming or converting enzymes. *Nat. Commun.*, 13(1), 7458. (2022).

[4] Li, F., Yang, L. C., Zhang, J., Chen, J. S., & Renata, H. Stereoselective synthesis of β-branched aromatic α-amino acids by biocatalytic dynamic kinetic resolution. *Angew. Chem. Int. Ed.*, 60(32), 17680-17685. (2021).

[5] Romney, D. K., Sarai, N. S., & Arnold, F. H. Nitroalkanes as versatile nucleophiles for enzymatic synthesis of noncanonical amino acids. *ACS Catal.*, 9(9), 8726-8730. (2019).

[6] Alias, M., Lopez, M. P., & Cativiela, C. An efficient and stereodivergent synthesis of threo- and erythro-β-methylphenylalanine. Resolution of each racemic pair by semipreparative HPLC. *Tetrahedron*, 60(4), 885-891. (2004).
