# Supplementary material for: Direct asymmetric synthesis of β-branched aromatic α-amino acids using engineered phenylalanine ammonia lyases
Source: Nat Commun. 2024 Sep 26;15:8264. doi: 10.1038/s41467-024-52613-x (PMC11427684; doi:10.1038/s41467-024-52613-x)
Supplement: Supplementary file 3 — Reporting Summary [file 41467_2024_52613_MOESM3_ESM.pdf]

nature portfolio

Corresponding author(s): Uwe T. Bornscheuer; Guifa Zhai;Chenghai Sun.

Last updated by author(s): Sep 13, 2024

Reporting Summary

Nature Portfolio wishes to improve the reproducibility of the work that we publish. This form provides structure for consistency and transparency in reporting. For further information on Nature Portfolio policies, see our Editorial Policies and the Editorial Policy Checklist.

Statistics

For all statistical analyses, confirm that the following items are present in the figure legend, table legend, main text, or Methods section.

n/a	Confirmed	
☐	☒	The exact sample size (n) for each experimental group/condition, given as a discrete number and unit of measurement
☐	☒	A statement on whether measurements were taken from distinct samples or whether the same sample was measured repeatedly
☒	☐	The statistical test(s) used AND whether they are one- or two-sided<br>*Only common tests should be described solely by name; describe more complex techniques in the Methods section.*
☒	☐	A description of all covariates tested
☒	☐	A description of any assumptions or corrections, such as tests of normality and adjustment for multiple comparisons
☐	☒	A full description of the statistical parameters including central tendency (e.g. means) or other basic estimates (e.g. regression coefficient) AND variation (e.g. standard deviation) or associated estimates of uncertainty (e.g. confidence intervals)
☒	☐	For null hypothesis testing, the test statistic (e.g. F, t, r) with confidence intervals, effect sizes, degrees of freedom and P value noted<br>*Give P values as exact values whenever suitable.*
☒	☐	For Bayesian analysis, information on the choice of priors and Markov chain Monte Carlo settings
☒	☐	For hierarchical and complex designs, identification of the appropriate level for tests and full reporting of outcomes
☒	☐	Estimates of effect sizes (e.g. Cohen's d, Pearson's r), indicating how they were calculated

Our web collection on statistics for biologists contains articles on many of the points above.

Software and code

Policy information about availability of computer code

Data collection	Collection of Protein concentration data: NanoDrop 1000 (version 3.8). Collection of HPLC data: SHIMADZU LC-30A UPLC system and SHIMADZU LC-20A HPLC system. Collection of NMR data: Bruker DRX-400 or DRX-600 NMR spectrometers (Bruker Co. Ltd., Germany). Protein modeling: AlphaFold 2. Docking: AutoDock (version 1.5.6). Conventional molecular dynamics (cMD): AMBER 22. Quantum mechanics/ molecular mechanics (QM/MM) simulations: ORCA 5.04. Umbrella sampling simulations: XTB 6.6.1. Primer design and calculation of annealing temperature data: NEBaseChanger (Version 1.3.3).
Data analysis	GraphPad Prism (version 8.0.1) was used to draw charts. Pymol (version 1.8.0.2) was used for preparing enzyme structure figures. MestReNova (version 14.0.0) was used for analysis of NMR data. Olex2 (version 1.5) was used to visualize the X-ray crystal structures of products. ChemDraw (version 19.0) was used to draw the schemes. Microsoft Excel (version 2010) was employed for statistical analysis. Microsoft Power Point (version 2010) was used to prepare and combine figures.

For manuscripts utilizing custom algorithms or software that are central to the research but not yet described in published literature, software must be made available to editors and reviewers. We strongly encourage code deposition in a community repository (e.g. GitHub). See the Nature Portfolio guidelines for submitting code & software for further information.

Data

Policy information about availability of data

All manuscripts must include a data availability statement. This statement should provide the following information, where applicable:

- Accession codes, unique identifiers, or web links for publicly available datasets
- A description of any restrictions on data availability
- For clinical datasets or third party data, please ensure that the statement adheres to our policy

Crystallographic data for the structures reported in this article has been deposited at the Cambridge Crystallographic Data Centre, under deposition no. CCDC 2293061 (4a), 2293062 (8a), 2293063 (5a) and 2313111 (9a). Copies of the data can be obtained free of charge via https://www.ccdc.cam.ac.uk/structures. All other characterization data and detailed experimental procedures are available in the supplementary materials.

Research involving human participants, their data, or biological material

Policy information about studies with human participants or human data. See also policy information about sex, gender (identity/presentation), and sexual orientation and race, ethnicity and racism.

Reporting on sex and gender	not relevant to our study.
Reporting on race, ethnicity, or other socially relevant groupings	not relevant to our study.
Population characteristics	not relevant to our study.
Recruitment	not relevant to our study.
Ethics oversight	not relevant to our study.

Note that full information on the approval of the study protocol must also be provided in the manuscript.

Field-specific reporting

Please select the one below that is the best fit for your research. If you are not sure, read the appropriate sections before making your selection.

☒ Life sciences ☐ Behavioural & social sciences ☐ Ecological, evolutionary & environmental sciences

For a reference copy of the document with all sections, see nature.com/documents/nr-reporting-summary-flat.pdf

Life sciences study design

All studies must disclose on these points even when the disclosure is negative.

Sample size	A sample size of 3 or 1 was used for most of the experiments.
Data exclusions	No data were excluded from the study.
Replication	All attempts were repeated three time at same time.
Randomization	Randomization is not relevant to this study.
Blinding	Blinding and randomization were not relevant to our study because our experiments were focused on rational enzyme design and modification based on prior computational predictions. The experimental groups and controls were specifically designed to test the hypotheses derived from these computational results. Therefore, randomization or blinding was not applicable in this context

Reporting for specific materials, systems and methods

We require information from authors about some types of materials, experimental systems and methods used in many studies. Here, indicate whether each material, system or method listed is relevant to your study. If you are not sure if a list item applies to your research, read the appropriate section before selecting a response.

Materials & experimental systems

n/a	Involved in the study
☒	☐ Antibodies
☒	☐ Eukaryotic cell lines
☒	☐ Palaeontology and archaeology
☒	☐ Animals and other organisms
☒	☐ Clinical data
☒	☐ Dual use research of concern
☒	☐ Plants

Methods

n/a	Involved in the study
☒	☐ ChIP-seq
☒	☐ Flow cytometry
☒	☐ MRI-based neuroimaging

Plants

Seed stocks	*Report on the source of all seed stocks or other plant material used. If applicable, state the seed stock centre and catalogue number. If plant specimens were collected from the field, describe the collection location, date and sampling procedures.*
Novel plant genotypes	*Describe the methods by which all novel plant genotypes were produced. This includes those generated by transgenic approaches, gene editing, chemical/radiation-based mutagenesis and hybridization. For transgenic lines, describe the transformation method, the number of independent lines analyzed and the generation upon which experiments were performed. For gene-edited lines, describe the editor used, the endogenous sequence targeted for editing, the targeting guide RNA sequence (if applicable) and how the editor was applied.*
Authentication	*Describe any authentication procedures for each seed stock used or novel genotype generated. Describe any experiments used to assess the effect of a mutation and, where applicable, how potential secondary effects (e.g. second site T-DNA insertions, mosiacism, off-target gene editing) were examined.*
