# Supplementary material for: Direct asymmetric synthesis of β-branched aromatic α-amino acids using engineered phenylalanine ammonia lyases
Source: Nat Commun. 2024 Sep 26;15:8264. doi: 10.1038/s41467-024-52613-x (PMC11427684; doi:10.1038/s41467-024-52613-x)

checkCIF/PLATON report

Structure factors have been supplied for datablock(s) 20230817-s8

THIS REPORT IS FOR GUIDANCE ONLY. IF USED AS PART OF A REVIEW PROCEDURE FOR PUBLICATION, IT SHOULD NOT REPLACE THE EXPERTISE OF AN EXPERIENCED CRYSTALLOGRAPHIC REFEREE.

No syntax errors found. CIF dictionary Interpreting this report

Datablock: 20230817-s8

```
Bond precision:   C-C = 0.0098 A          Wavelength=1.54184

Cell:            a=8.24139(12)    b=5.38734(7)        c=14.6796(2)
                 alpha=90         beta=99.7698(13)    gamma=90
Temperature:     300 K
```

	Calculated	Reported
Volume	642.310(16)	642.312(15)
Space group	P 21	P 1 21 1
Hall group	P 2yb	P 2yb
Moiety formula	C10 H12 I N O2, 2(H2 O)	C10 H12 I N O2, 2(H2 O)
Sum formula	C10 H16 I N O4	C10 H16 I N O4
Mr	341.14	341.14
Dx,g cm-3	1.764	1.764
Z	2	2
Mu (mm-1)	19.616	19.616
F000	336.0	336.0
F000’	336.43	
h,k,lmax	10,6,18	10,6,18
Nref	2695[1497]	2522
Tmin,Tmax	0.180,0.208	0.191,1.000
Tmin’	0.063	

```
Correction method= # Reported T Limits: Tmin=0.191 Tmax=1.000
AbsCorr = MULTI-SCAN

Data completeness= 1.68/0.94        Theta(max)= 76.197

R(reflections)= 0.0457( 2434)                          wR2(reflections)=
                                                       0.1333( 2522)
S = 1.104                   Npar= 147
```

The following ALERTS were generated. Each ALERT has the format
test-name_ALERT_alert-type_alert-level.
Click on the hyperlinks for more details of the test.

Alert level C

```
PLAT342_ALERT_3_C Low Bond Precision on  C-C Bonds ..............     0.0098 Ang.
```

Alert level G

```
PLAT007_ALERT_5_G Number of Unrefined Donor-H Atoms .............          7 Report
PLAT790_ALERT_4_G Centre of Gravity not Within Unit Cell: Resd.  #         3 Note
              H2 O
PLAT912_ALERT_4_G Missing # of FCF Reflections Above STh/L=  0.600        45 Note
PLAT941_ALERT_3_G Average HKL Measurement Multiplicity ...........       4.6 Low
PLAT961_ALERT_5_G Dataset Contains no Negative Intensities .......    Please Check
PLAT978_ALERT_2_G Number C-C Bonds with Positive Residual Density.         0 Info
```

0 **ALERT level A** = Most likely a serious problem - resolve or explain
0 **ALERT level B** = A potentially serious problem, consider carefully
1 **ALERT level C** = Check. Ensure it is not caused by an omission or oversight
6 **ALERT level G** = General information/check it is not something unexpected

0 ALERT type 1 CIF construction/syntax error, inconsistent or missing data
1 ALERT type 2 Indicator that the structure model may be wrong or deficient
2 ALERT type 3 Indicator that the structure quality may be low
2 ALERT type 4 Improvement, methodology, query or suggestion
2 ALERT type 5 Informative message, check

It is advisable to attempt to resolve as many as possible of the alerts in all categories. Often the minor alerts point to easily fixed oversights, errors and omissions in your CIF or refinement strategy, so attention to these fine details can be worthwhile. In order to resolve some of the more serious problems it may be necessary to carry out additional measurements or structure refinements. However, the purpose of your study may justify the reported deviations and the more serious of these should normally be commented upon in the discussion or experimental section of a paper or in the "special_details" fields of the CIF. checkCIF was carefully designed to identify outliers and unusual parameters, but every test has its limitations and alerts that are not important in a particular case may appear. Conversely, the absence of alerts does not guarantee there are no aspects of the results needing attention. It is up to the individual to critically assess their own results and, if necessary, seek expert advice.

Publication of your CIF in IUCr journals

A basic structural check has been run on your CIF. These basic checks will be run on all CIFs submitted for publication in IUCr journals (*Acta Crystallographica*, *Journal of Applied Crystallography*, *Journal of Synchrotron Radiation*); however, if you intend to submit to *Acta Crystallographica Section C* or *E* or *IUCrData*, you should make sure that full publication checks are run on the final version of your CIF prior to submission.

Publication of your CIF in other journals

Please refer to the *Notes for Authors* of the relevant journal for any special instructions relating to CIF submission.

PLATON version of 06/07/2023; check.def file version of 30/06/2023

Datablock 20230817-s8 - ellipsoid plot

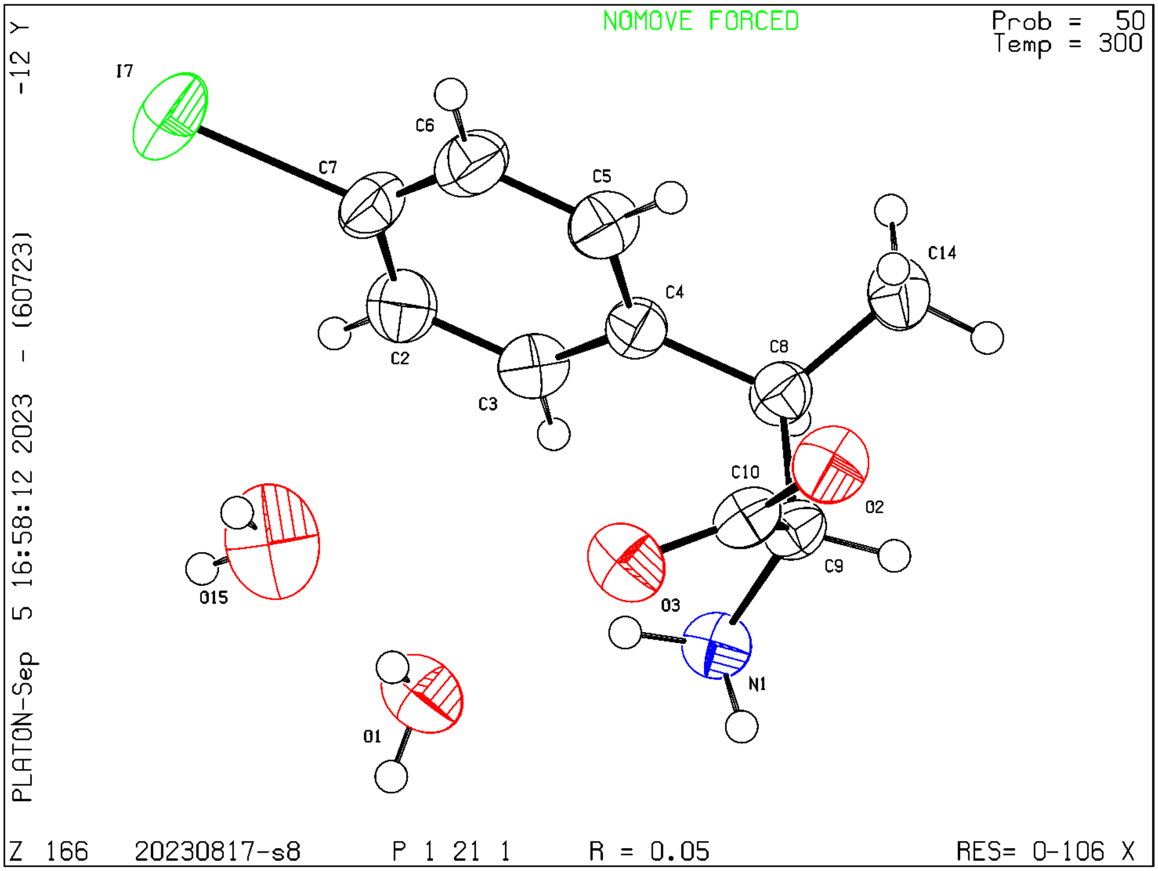

Supplement: Supplementary file 5 — Source data 2 [file 41467_2024_52613_MOESM5_ESM.zip › CCDC 2293061_compound p4-checkcif.pdf]
