# Supplementary material for: Direct asymmetric synthesis of β-branched aromatic α-amino acids using engineered phenylalanine ammonia lyases
Source: Nat Commun. 2024 Sep 26;15:8264. doi: 10.1038/s41467-024-52613-x (PMC11427684; doi:10.1038/s41467-024-52613-x)

checkCIF/PLATON report

Structure factors have been supplied for datablock(s) 20230817-s15_auto

THIS REPORT IS FOR GUIDANCE ONLY. IF USED AS PART OF A REVIEW PROCEDURE FOR PUBLICATION, IT SHOULD NOT REPLACE THE EXPERTISE OF AN EXPERIENCED CRYSTALLOGRAPHIC REFEREE.

No syntax errors found. CIF dictionary Interpreting this report

Datablock: 20230817-s15_auto

Bond precision: C-C = 0.0033 A Wavelength=1.54184

Cell:	a=13.7630(4)	b=5.5065(2)	c=14.2159(4)
	alpha=90	beta=102.629(3)	gamma=90
Temperature:	300 K		

	Calculated	Reported
Volume	1051.30(6)	1051.30(6)
Space group	P 21	P 1 21 1
Hall group	P 2yb	P 2yb
Moiety formula	C10 H12 F N O2, H2 O	C10 H12 F N O2, H2 O
Sum formula	C10 H14 F N O3	C10 H14 F N O3
Mr	215.22	215.22
Dx,g cm-3	1.360	1.360
Z	4	4
Mu (mm-1)	0.948	0.948
F000	456.0	456.0
F000’	457.66	
h,k,lmax	17,6,17	17,6,17
Nref	4378[2427]	3738
Tmin,Tmax	0.903,0.927	0.665,1.000
Tmin’	0.892	

Correction method= # Reported T Limits: Tmin=0.665 Tmax=1.000
AbsCorr = MULTI-SCAN

Data completeness= 1.54/0.85 Theta(max)= 76.074

R(reflections)= 0.0329(3513) wR2(reflections)= 0.0876(3738)

S = 1.055 Npar= 281

The following ALERTS were generated. Each ALERT has the format
test-name_ALERT_alert-type_alert-level.
Click on the hyperlinks for more details of the test.

Alert level C

PLAT260_ALERT_2_C Large Average Ueq of Residue Including O30 0.101 Check
PLAT911_ALERT_3_C Missing FCF Refl Between Thmin & STh/L= 0.600 18 Report
PLAT915_ALERT_3_C No Flack x Check Done: Low Friedel Pair Coverage 74 %

Alert level G

PLAT007_ALERT_5_G Number of Unrefined Donor-H Atoms .... 10 Report
PLAT790_ALERT_4_G Centre of Gravity not Within Unit Cell: Resd. # 3 Note
H2 O
PLAT912_ALERT_4_G Missing # of FCF Reflections Above STh/L= 0.600 114 Note
PLAT978_ALERT_2_G Number C-C Bonds with Positive Residual Density. 0 Info

0 **ALERT level A** = Most likely a serious problem - resolve or explain
0 **ALERT level B** = A potentially serious problem, consider carefully
3 **ALERT level C** = Check. Ensure it is not caused by an omission or oversight
4 **ALERT level G** = General information/check it is not something unexpected

0 ALERT type 1 CIF construction/syntax error, inconsistent or missing data
2 ALERT type 2 Indicator that the structure model may be wrong or deficient
2 ALERT type 3 Indicator that the structure quality may be low
2 ALERT type 4 Improvement, methodology, query or suggestion
1 ALERT type 5 Informative message, check

It is advisable to attempt to resolve as many as possible of the alerts in all categories. Often the minor alerts point to easily fixed oversights, errors and omissions in your CIF or refinement strategy, so attention to these fine details can be worthwhile. In order to resolve some of the more serious problems it may be necessary to carry out additional measurements or structure refinements. However, the purpose of your study may justify the reported deviations and the more serious of these should normally be commented upon in the discussion or experimental section of a paper or in the "special_details" fields of the CIF. checkCIF was carefully designed to identify outliers and unusual parameters, but every test has its limitations and alerts that are not important in a particular case may appear. Conversely, the absence of alerts does not guarantee there are no aspects of the results needing attention. It is up to the individual to critically assess their own results and, if necessary, seek expert advice.

Publication of your CIF in IUCr journals

A basic structural check has been run on your CIF. These basic checks will be run on all CIFs submitted for publication in IUCr journals (*Acta Crystallographica*, *Journal of Applied Crystallography*, *Journal of Synchrotron Radiation*); however, if you intend to submit to *Acta Crystallographica Section C* or *E* or *IUCrData*, you should make sure that full publication checks are run on the final version of your CIF prior to submission.

Publication of your CIF in other journals

Please refer to the *Notes for Authors* of the relevant journal for any special instructions relating to CIF submission.

PLATON version of 06/07/2023; check.def file version of 30/06/2023

Datablock 20230817-s15_auto - ellipsoid plot

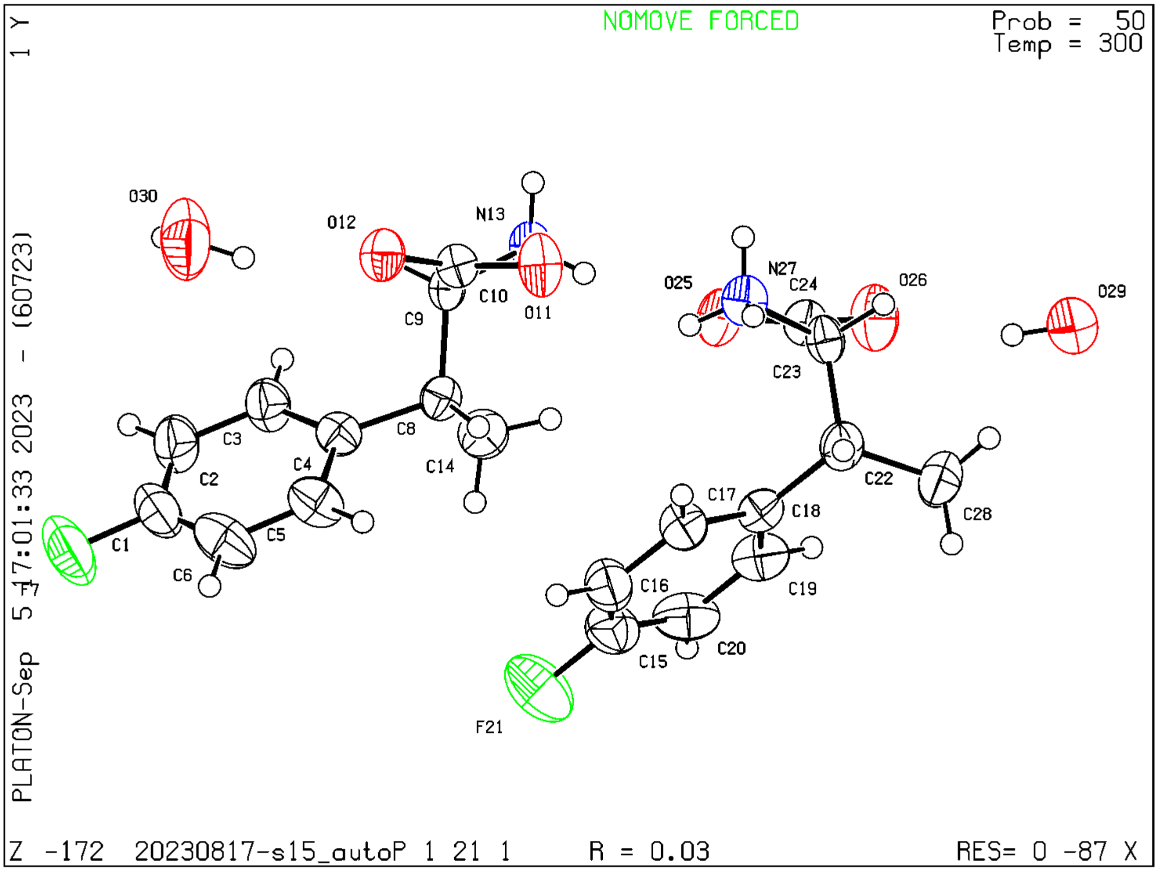

Supplement: Supplementary file 5 — Source data 2 [file 41467_2024_52613_MOESM5_ESM.zip › CDCC 2293062_compound p8-checkcif.pdf]
