# Supplementary material for: Direct asymmetric synthesis of β-branched aromatic α-amino acids using engineered phenylalanine ammonia lyases
Source: Nat Commun. 2024 Sep 26;15:8264. doi: 10.1038/s41467-024-52613-x (PMC11427684; doi:10.1038/s41467-024-52613-x)

checkCIF/PLATON report

Structure factors have been supplied for datablock(s) 20231114_1

THIS REPORT IS FOR GUIDANCE ONLY. IF USED AS PART OF A REVIEW PROCEDURE FOR PUBLICATION, IT SHOULD NOT REPLACE THE EXPERTISE OF AN EXPERIENCED CRYSTALLOGRAPHIC REFEREE.

No syntax errors found. CIF dictionary Interpreting this report

Datablock: 20231114_1

Bond precision:	C-C = 0.0027 A		Wavelength=1.54184
Cell:	a=5.8720(1)	b=6.4774(1)	c=16.5959(2)
	alpha=90	beta=90.612(1)	gamma=90
Temperature:	100 K		

	Calculated	Reported
Volume	631.194(16)	631.194(16)
Space group	P 21	P 1 21 1
Hall group	P 2yb	P 2yb
Moiety formula	C14 H15 N O2, H2 O	H2 O, C14 H15 N O2
Sum formula	C14 H17 N O3	C14 H17 N O3
Mr	247.29	247.28
Dx,g cm-3	1.301	1.301
Z	2	2
Mu (mm-1)	0.746	0.746
F000	264.0	264.0
F000'	264.83	
h,k,lmax		7,7,20
Nref		2428
Tmin,Tmax	0.851,0.956	0.805,1.000
Tmin'	0.742	

Correction method= # Reported T Limits: Tmin=0.805 Tmax=1.000
AbsCorr = MULTI-SCAN

Data completeness= Theta(max)= 74.301

R(reflections)= 0.0272(2391) wR2(reflections)= 0.0755(2428)

S = 1.040 Npar= 169

The following ALERTS were generated. Each ALERT has the format
test-name_ALERT_alert-type_alert-level.
Click on the hyperlinks for more details of the test.

Alert level C

```
PLAT042_ALERT_1_C Calc. and Reported MoietyFormula Strings  Differ     Please Check
              Calc: C14 H15 N O2, H2 O
              Rep.: H2 O, C14 H15 N O2
PLAT911_ALERT_3_C Missing FCF Refl Between Thmin & STh/L=    0.600           2 Report
                7  0  2,   2  0 19,
```

Alert level G

```
PLAT007_ALERT_5_G Number of Unrefined Donor-H Atoms .............           5 Report
              H00A  H00B  H00C  H00O  H00P
PLAT720_ALERT_4_G Number of Unusual/Non-Standard Labels ..........         35 Note
              O001    H00O    H00P    O002    O003    N004    H00A    H00B
              H00C    C005    C006    C007    H007    C008    C009    C00A
              H00D    C00B    H00E    C00C    H00F    C00D    H00G    C00E
              H00H    C00F    H00I    C00G    H00J    C00H    H00K    H00L
              H00M    C00I    H00N
PLAT912_ALERT_4_G Missing # of FCF Reflections Above STh/L=  0.600         25 Note
PLAT978_ALERT_2_G Number C-C Bonds with Positive Residual Density.          3 Info
```

0 **ALERT level A** = Most likely a serious problem - resolve or explain
0 **ALERT level B** = A potentially serious problem, consider carefully
2 **ALERT level C** = Check. Ensure it is not caused by an omission or oversight
4 **ALERT level G** = General information/check it is not something unexpected

1 ALERT type 1 CIF construction/syntax error, inconsistent or missing data
1 ALERT type 2 Indicator that the structure model may be wrong or deficient
1 ALERT type 3 Indicator that the structure quality may be low
2 ALERT type 4 Improvement, methodology, query or suggestion
1 ALERT type 5 Informative message, check

It is advisable to attempt to resolve as many as possible of the alerts in all categories. Often the minor alerts point to easily fixed oversights, errors and omissions in your CIF or refinement strategy, so attention to these fine details can be worthwhile. In order to resolve some of the more serious problems it may be necessary to carry out additional measurements or structure refinements. However, the purpose of your study may justify the reported deviations and the more serious of these should normally be commented upon in the discussion or experimental section of a paper or in the "special_details" fields of the CIF. checkCIF was carefully designed to identify outliers and unusual parameters, but every test has its limitations and alerts that are not important in a particular case may appear. Conversely, the absence of alerts does not guarantee there are no aspects of the results needing attention. It is up to the individual to critically assess their own results and, if necessary, seek expert advice.

Publication of your CIF in IUCr journals

A basic structural check has been run on your CIF. These basic checks will be run on all CIFs submitted for publication in IUCr journals (*Acta Crystallographica*, *Journal of Applied Crystallography*, *Journal of Synchrotron Radiation*); however, if you intend to submit to *Acta Crystallographica Section C* or *E* or *IUCrData*, you should make sure that full publication checks are run on the final version of your CIF prior to submission.

Publication of your CIF in other journals

Please refer to the *Notes for Authors* of the relevant journal for any special instructions relating to CIF submission.

PLATON version of 29/11/2023; check.def file version of 14/09/2023

Datablock 20231114_1 - ellipsoid plot

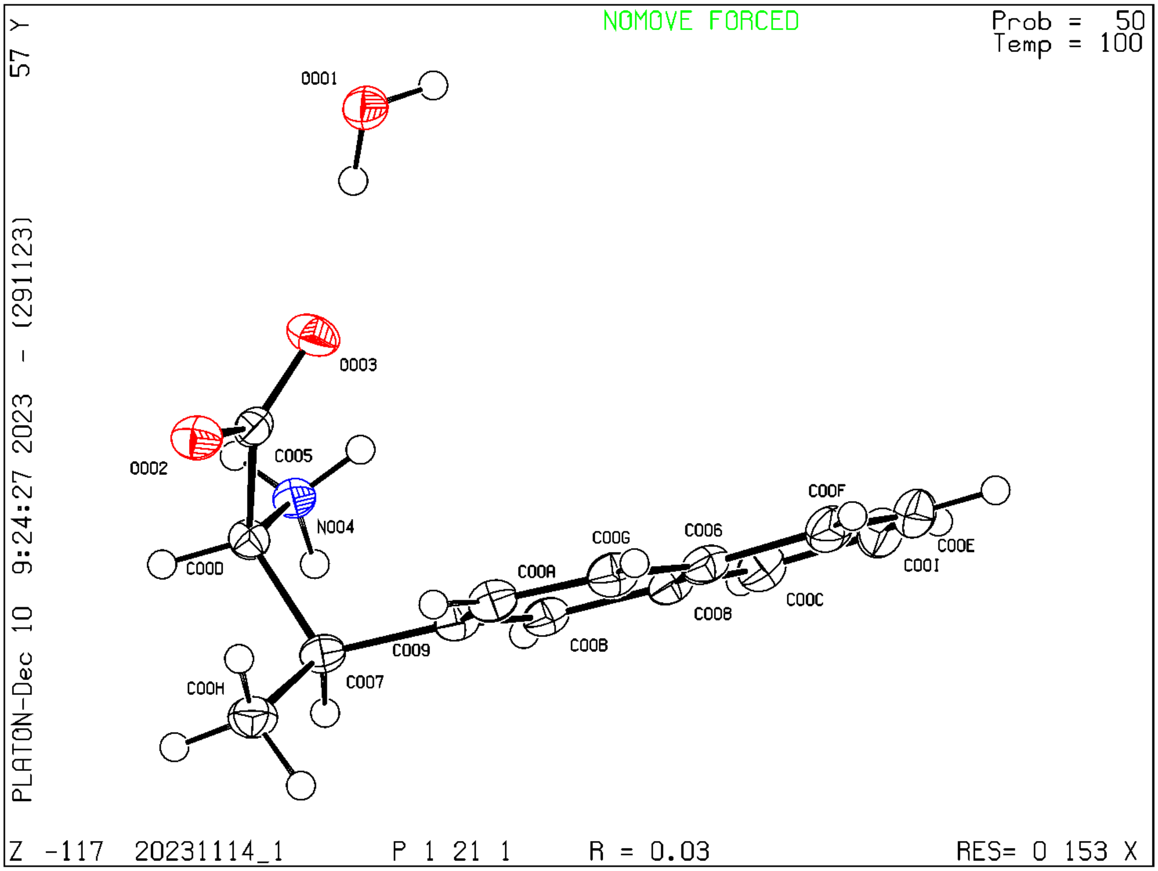

Supplement: Supplementary file 5 — Source data 2 [file 41467_2024_52613_MOESM5_ESM.zip › CDCC 2313111_compound p9-checkcif.pdf]
