# Supplementary figures and images for: Direct asymmetric synthesis of β-branched aromatic α-amino acids using engineered phenylalanine ammonia lyases
Source: Nat Commun. 2024 Sep 26;15:8264. doi: 10.1038/s41467-024-52613-x (PMC11427684; doi:10.1038/s41467-024-52613-x)

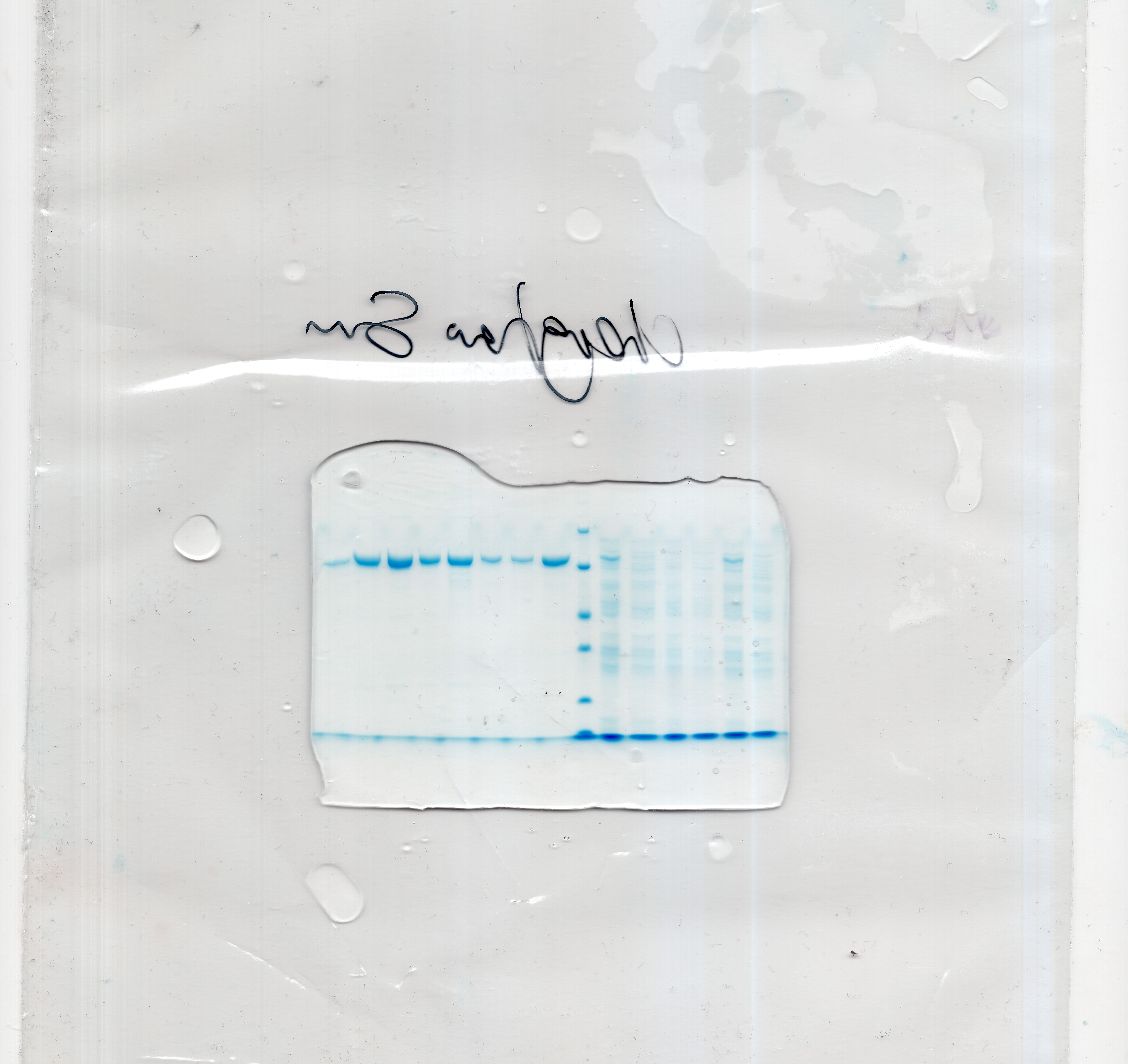

Supplement: Supplementary file 9 — Source data 6 [file 41467_2024_52613_MOESM9_ESM.zip › uncropped SDS page results.tif.jpg]
